# Synthesis and Evaluation of MGB Polyamide-Oligonucleotide Conjugates as Gene Expression Control Compounds

**DOI:** 10.1155/2023/2447998

**Published:** 2023-03-14

**Authors:** Kazuo Kamaike, Mutsumi Sano, Daisuke Sakata, Yu Nishihara, Hiroaki Amino, Akihiro Ohtsuki, Yui Okada, Takafumi Miyakawa, Makoto Kogawara, Mai Tsutsumi, Misato Takahashi, Etsuko Kawashima, Koichiro Ota, Hiroaki Miyaoka

**Affiliations:** School of Pharmacy, Tokyo University of Pharmacy and Life Sciences, 1432-1 Horinouchi, Hachioji, Tokyo 192-0392, Japan

## Abstract

MGB polyamide-oligonucleotide conjugates **ON 1**-**4** with linked MGB polyamides at the 2-exocyclic amino group of a guanine base using aminoalkyl linkers were synthesized and evaluated in terms of binding affinity for complementary DNA containing the MGB polyamide binding sequence using *T*_m_ and CD analyses. The MGB polyamides comprised pyrrole polyamides (Py_4_- and Py_3_-), which possess binding affinity for A-T base pairs, and imidazole (Im_3_-) and pyrrole-*γ*-imidazole (Py_3_-*γ*-Im_3_-) polyamide hairpin motifs, which possess binding affinity for C-G base pairs. It was found that the stability of modified dsDNA was greatly influenced by the linker length. Py_4_- and Py_3_-oligonucleotide conjugates (**ON 1** (*n* = 4) and **ON 2** (*n* = 4)) containing the 4-aminobutyl linker formed stable dsDNA with complementary DNA. Although Im_3_-oligonucleotide conjugate **ON 3** (*n* = 4) containing the 4-aminobutyl linker formed stable dsDNA with complementary DNA, stabilization of dsDNA by the imidazole amide moiety of **ON 3** (*n* = 4) was lower compared with the pyrrole amide moiety of **ON 2** (*n* = 4). The Py_3_-*γ*-Im_3_-oligonucleotide conjugate **ON 4** (*n* = 2), which possesses binding affinity for C-G base pairs via a pyrrole/imidazole combination and contains a 2-aminoethyl linker, showed high binding ability for complementary DNA. Furthermore, the DNA sequence recognition of MGB polyamide-oligonucleotide conjugates was investigated using single-base mismatch DNAs, which possess a mismatch base in the MGB polyamide binding sequence. The Py_3_-*γ*-Im_3_-oligonucleotide conjugate **ON 4** (*n* = 2) showed high sequence recognition ability for complementary DNA.

## 1. Introduction

**Figure 1:**
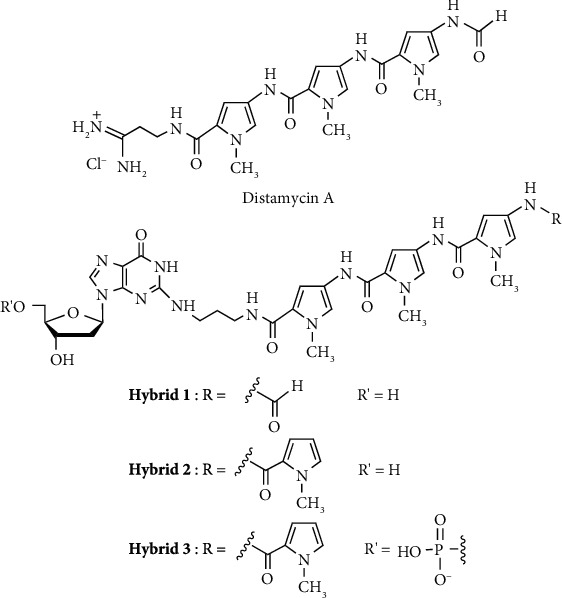
Structures of distamycin A, hybrids **1**, **2**, and **3**.

**Figure 2:**
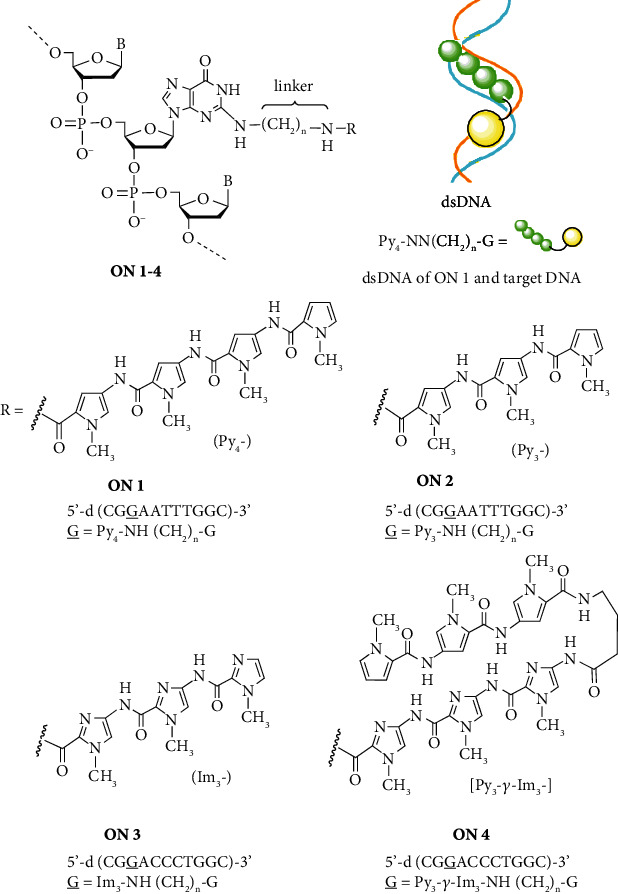
MGB polyamide-oligonucleotide conjugates.

Numerous nucleic acid analogues have been synthesized and characterized as potential gene therapy agents [1, 2]. We previously designed and synthesized nucleoside (**Hybrid 1**) linked to pyrrole polyamide minor groove binder (MGB) comprising modified distamycin A, which possesses a high affinity for the 5′-d(AATTT)-3′/3′-(TTAAA)-5′ sequence of double-stranded DNA (dsDNA) [3–8], as a lead compound for the development of potential gene therapy agents [9–12]. When the MGB polyamide-nucleoside hybrid interacts with dsDNA, it is expected that complex formation would involve high affinity and sequence selectivity. If the hybrid is incorporated into DNA during DNA biosynthesis, it is expected that DNA replication and transcription would be obstructed through minor groove binding of the hybrid polyamide moiety. The dsDNA binding ability of **Hybrid 1** was investigated via melting temperature (*T*_m_) and circular dichroism (CD) analyses (Figure 1) [5, 6]. It was shown that **Hybrid 1** possessed greater binding specificity compared with distamycin A [12]. Then, in an effort to examine the development of potential antisense drugs, we synthesized oligonucleotide **ON 1** (*n* = 3) conjugated to **Hybrid 2***in lieu of ***Hybrid 1** containing the formyl group which is unstable under the basic conditions of deprotection during oligonucleotide solid-phase synthesis, and subsequently examined the binding ability of **ON 1** (*n* = 3) to complementary DNA (Figure 2) [13]. Dervan et al. [14], Zamecnik et al. [15], Novopashina et al. and Boutorine et al. [16–21] have reported the synthesis and evaluation of oligonucleotides conjugated with one or two MGB polyamides to either the 5′- or 3′-ends. Sequence-specific stabilization of DNA duplexes and DNA triplexes by MGB polyamides conjugated to one DNA strand was shown. It was expected that oligonucleotides conjugated with the MGB polyamide to the 2-exocyclic amino group of a guanine base, which is positioned above the floor of the minor groove of the DNA duplex, would possess high DNA binding ability. **ON 1** (*n* = 3), which includes a modified guanosine (**G**) in the 5′ direction in the oligonucleotide chain given the preferred orientation of the polyamide in the minor groove of dsDNA (*C*-(pyrrole polyamide)-*N*/3′-(TTAAA)-5′ of the complementary target DNA) [22–24], was synthesized and evaluated as a model oligonucleotide [13]. From the *T*_m_ and CD analyses, it was found that **ON 1** (*n* = 3) formed stable dsDNA with complementary DNA via action of the pyrrole amide moiety. From this result, it is expected that MGB polyamide-oligonucleotide conjugates could be effective gene expression control compounds and that MGB polyamide-2′-deoxyguanosine hybrid might be of potential use as a sequence-specific gene therapy agent based on potential obstruction of DNA replication and transcription. The inhibition of mouse mammary carcinoma FM3A cell growth by pyrrole polyamide compounds (Hybrids and distamycin A) has been evaluated (Figure 1) [25]. It was found that hybrids induce dose-dependent inhibition of cell growth. In particular, **Hybrid 3** bearing a 5′-phosphate group, which is a suitable substrate for biosynthesis, exhibited the highest inhibition.

The binding ability of pyrrole polyamide-oligonucleotide conjugates to target DNA, and the inhibition of cell growth by pyrrole polyamide-2′-deoxyguanosine hybrids should be greatly influenced by the chain length of the pyrrole polyamide moiety and the length of the linker between the pyrrole polyamide moiety and the guanine base. Although we previously reported the synthesis of MGB polyamide-oligonucleotide conjugate **ON 1** (*n* = 3), with linked pyrrole amide tetramer (Py_4_-) at the 2-exocyclic amino group of a guanine base using 3-aminopropyl linker and evaluated the stability of modified dsDNA as described above [13], an examination of the length of the chain or linker connecting the pyrrole polyamide moiety to the guanine base and the DNA sequence recognition ability had not been investigated. In an effort to improve the activity of pyrrole polyamide-oligonucleotide conjugates, we performed the synthesis and evaluation of **ON 1** and **ON 2** with linked pyrrole polyamides (Py_4_- and Py_3_-) using various aminoalkyl linkers in terms of binding affinity for complementary DNA (Figure 2).

In addition to pyrrole polyamides, which possess high affinity for A-T base pairs of dsDNA, pyrrole-imidazole polyamides, which possess high affinity for C-G base pairs of dsDNA, were reported by Dervan et al. [14, 22–24, 26–31]. Furthermore, the synthesis and evaluation of MGB polyamide-oligonucleotide conjugates which possess binding affinity for C-G base pairs are an important aspect of the study. We performed the synthesis and evaluation of **ON 3** and **ON 4** with conjugated MGB polyamides (imidazole polyamide (Im_3_-) and pyrrole-*γ*-imidazole polyamide hairpin motif (Py_3_-*γ*-Im_3_-)) at the 2-exocyclic amino group of a guanine base using various aminoalkyl linkers (Figure 2).

Herein, we report on the synthesis and evaluation of MGB polyamide-oligonucleotide conjugates **ON 1**-**4** using various aminoalkyl linkers.

## 2. Materials and Methods

Column chromatography was performed on silica gel (Kanto Chemical Silica gel N60, spherical, natural, 40-50 *μ*m). Precoated silica gel plates with a fluorescent indicator (Merck 60 F254) were used for analytical TLC. HPLC was performed on a Waters liquid chromatograph (600E system) equipped with a UV-VIS detector (2487 Dual), data module (741 type), and fraction collector. A *μ*Bondasphare C18 5 *μ*m 100A (3.9 mm ID × 150 mm L) column with gradients of 5-50% CH_3_CN in water (0.01 M TEAA, pH 7) was used. ^1^H-NMR and ^13^C-NMR spectra were recorded using Bruker DRX 400 and a Bruker Biospin AVANCE III HD 400 instruments. Mass spectra were recorded on a Micromass Q-Tof Ultima API and a Micromass LCT spectrometer with a time-of-flight analyser. Elemental analyses were performed using an Elemental Vavio EL apparatus. Circular Dichroism (CD) spectra were recorded on a JASCO J-720 spectropolarimeter. UV melting curves were measured using a Shimadzu TMSPC-8/UV-1600 apparatus. UV spectra were recorded using a Shimadzu UV-1200 apparatus. DNA oligonucleotides were purchased from Hokkaido System Science Co., Ltd. Compounds **2**, **3**, **4**, **5**, **7**, **9,** and **12** were prepared as previously described [9–13]. The CPG support-bound 2′-deoxynucleoside **16** (B = C^Bz^) and 2′-deoxynucleoside 3′-phosphoramidites **17** (B = T, C^Bz^, A^Bz^, and G^iBu^) were purchased from Glen Research Corporation. 2′-Deoxy-2-fluoroinosine 3′-phosphoramidite **17** (B = I^F,NPE^) was prepared from 2′-deoxyguanosine as previously described [32–35]. Ethyl 1-methylimidazole-2-carboxylate (**20**) was prepared according to the procedure described by Baird and Dervan. [30].

### 2.1. Methyl 1-Methyl-4-[1-Methyl-4-(1-Methyl-1*H*-Pyrrole-2-Carbonyl)Amino-1*H*-Pyrrole-2-Carbonyl]Amino-1*H*-Pyrrole-2-Carboxylate (6)

Compound **2** [10–13] (2.83 g, 9.06 mmol) and 1-methyl-1*H*-pyrrole-2-carboxylic acid (**5**) [13] (1.26 g, 9.97 mmol) were dissolved in dichloromethane (45 mL), and then 1-ethyl-3-(3-dimethylaminopropyl)carbodiimide hydrochloride (EDCI) (2.61 g, 13.6 mmol) and 4-(*N*,*N*-dimethylamino)pyridine (DMAP) (1.66 g, 13.6 mmol) were added to the solution. After stirring at room temperature for 14 h, the solution was diluted with chloroform (300 mL) and washed with 2 M HCl aq. (150 mL x 3), H_2_O (150 mL x3), 5% NaHCO_3_ aq. (150 mL × 3), and H_2_O (150 mL). The organic layer was dried over anhydrous magnesium sulfate and evaporated to dryness. The residue was subjected to chromatographic separation on a column of silica gel using a 0-5% methanol/chloroform solvent system to give **6** (2.79 g, 80% yield) as a slightly brown glass. ^1^H-NMR (DMSO-*d*_6_): *δ* 9.92 (s, 1H, CONH), 9.82 (s, 1H, CONH), 7.46 (d, 1H, *J* = 1.9 Hz, Py-H), 7.23 (d, 1H, *J* = 1.8 Hz, Py-H), 7.05 (d, 1H, *J* = 1.9 Hz, Py-H), 6.94-6.90 (m, 3H, $\text{Py}-\underset{¯}{\text{H}}\times 3$), 6.06 (dd, 1H, *J* = 2.6, 3.9 Hz, Py-H), 3.88 (s, 3H, NCH_3_), 3.85 (s, 3H, NCH_3_), 3.84 (s, 3H, NCH_3_), 3.74 (s, 3H, OCH_3_); ^13^C-NMR (DMSO-*d*_6_): *δ* 160.8, 158.6, 158.5, 128.1, 125.5, 123.0, 122.5, 122.2, 120.7, 118.5, 112.6, 108.4, 106.6, 104.8, 50.9, 36.2, and 36.1, 36.0; HRMS (ESI-TOF) *m*/*z*: calcd for C_19_H_22_N_5_O_4_(M + H)^+^ 384.1672, found 384.1667; UV (CH_3_OH): *λ*_max_ 299, 238 nm, *λ*_min_ 260, and 222 nm; and *ε*_260_ 1.8 × 10^4^.

### 2.2. 2-[(9*H*-Fluoren-9-yl)Methoxycarbonylamino]Ethanaminium Chloride (8), 4-[(9*H*-Fluoren-9-yl)Methoxycarbonylamino]Butanaminium Chloride (10), and 5-[(9*H*-Fluoren-9-yl)Methoxycarbonylamino]Pentanaminium Chloride (11)

Compounds **8**, **10,** and **11** were prepared according to the synthetic procedure of 3-[(9*H*-fluoren-9-yl)methoxycarbonylamino]propanaminium chloride (**9**) [10–13].

**Compound 8**: (9*H*-Fluoren-9-yl)methyl phenyl carbonate (**7**) (0.95 g, 3.0 mmol) was suspended in methanol (12.5 mL), and then ethylenediamine (0.20 mL, 3.0 mmol) was added to the solution. After stirring for 4 h at room temperature, pyridinium hydrochloride (0.75 g, 6.5 mmol) was added, and the solution stirred for 10 min. The solution was concentrated *in vacuo*, and the residue was subjected to silica gel column chromatography using a methanol/chloroform (1 : 4 *v*/*v*) solvent system to give **8** (0.19 g, 20% yield) as a white powder. ^1^H-NMR (CD_3_OD): *δ* 7.80 (d, 2H, *J* = 7.5 Hz, Ar-H of the Fmoc group), 7.65 (d, 2H, *J* = 7.5 Hz, Ar-H of the Fmoc group), 7.40 (t, 2H, *J* = 7.4 Hz, Ar-H of the Fmoc group), 7.31 (t, 2H, *J* = 7.4 Hz, Ar-H of the Fmoc group), 4.42 (d, 2H, *J* = 6.6 Hz, CHCH_2_ of the Fmoc group), 4.22 (t, 1H, *J* = 6.6 Hz, CHCH_2_ of the Fmoc group), 3.32-3.30 (m, 2H, NCH_2_), and 2.92-2.90 (t, 2H, *J* = 6.0 Hz, NCH_2_); ^13^C-NMR (CD_3_OD): *δ* 157.9, 143.8, 141.2, 127.4, 126.8, 124.8, 119.6, 66.7, 47.1, 39.8, and 38.3; HRMS (ESI-TOF) *m*/*z*: calcd for C_17_H_19_N_2_O_2_(M + H)^+^ 283.1447, found 283.1455

**Compound 10**: (9*H*-Fluoren-9-yl)methyl phenyl carbonate (**7**) (3.16 g, 9.99 mmol) was suspended in methanol (42 mL), and then butane-1,4-diamine (1.0 mL, 9.95 mmol) was added to the solution. After stirring for 4 h at room temperature, pyridinium hydrochloride (2.51 g, 21.7 mmol) was added, and the solution stirred for 10 min. The solution was concentrated *in vacuo*, and the residue was subjected to silica gel column chromatography using a methanol/chloroform (1 : 4 *v*/*v*) solvent system to give **10** (2.11 g, 61% yield) as a white powder. ^1^H-NMR (CD_3_OD): *δ* 7.79 (d, 2H, *J* = 7.5 Hz, Ar-H of the Fmoc group), 7.64 (d, 2H, *J* = 7.4 Hz, Ar-H of the Fmoc group), 7.39 (t, 2H, *J* = 7.4 Hz, Ar-H of the Fmoc group), 7.31 (t, 2H, *J* = 7.3 Hz, Ar-H of the Fmoc group), 4.37 (d, 2H, *J* = 6.8 Hz, CHCH_2_ of the Fmoc group), 4.20 (t, 1H, *J* = 6.8 Hz, CHCH_2_ of the Fmoc group), 3.15 (t, 2H, *J* = 6.6 Hz, NCH_2_), 2.93 (t, 2H, *J* = 7.4 Hz, NCH_2_), and 1.66-1.55 (m, 4H, $\text{C}{\underset{¯}{\text{H}}}_{2}\times 2$); ^13^C-NMR (DMSO-*d*_6_): *δ* 156.4, 144.1, 140.9, 127.8, 127.3, 125.4, 120.3, 65.5, 46.9, 39.8, 38.6, 26.46, and 24.39; HRMS (ESI-TOF) *m*/*z*: calcd for C_19_H_23_N_2_O_2_(M + H)^+^ 311.1760, found 311.1752

**Compound 11**: (9*H*-Fluoren-9-yl)methyl phenyl carbonate (**7**) (3.16 g, 9.99 mmol) was suspended in methanol (42 mL), and then pentane-1,5-diamine (1.17 mL, 10.0 mmol) was added to the solution. After stirring for 4 h at room temperature, pyridinium hydrochloride (2.51 g, 21.7 mmol) was added and the solution stirred for 10 min. The solution was concentrated *in vacuo*, and the residue was subjected to silica gel column chromatography using a methanol/chloroform (1 : 4 *v*/*v*) solvent system to give **11** (1.64 g, 45% yield) as a white powder. ^1^H-NMR (CD_3_OD): *δ* 7.76 (d, 2H, *J* = 7.5 Hz, Ar-H of the Fmoc group), 7.60 (d, 2H, *J* = 7.4 Hz, Ar-H of the Fmoc group), 7.36 (t, 2H, *J* = 7.4 Hz, Ar-H of the Fmoc group), 7.26 (t, 2H, *J* = 7.4 Hz, Ar-H of the Fmoc group), 4.33 (d, 2H, *J* = 6.8 Hz, CHCH_2_ of the Fmoc group), 4.17 (t, 1H, *J* = 6.8 Hz, CHCH_2_ of the Fmoc group), 3.08 (t, 2H, *J* = 6.9 Hz, NCH_2_), 2.87 (t, 2H, *J* = 7.6 Hz, NCH_2_), 1.64-1.60 (m, 2H, CH_2_), 1.51-1.47 (m, 2H, CH_2_), and 1.38-1.34 (m, 2H, CH_2_); ^13^C-NMR (CD_3_OD): *δ* 158.9, 145.3, 142.6, 128.7, 128.1, 126.1, 120.1, 67.5, 48.4, 41.2, 40.6, 30.3, 28.2, and 24.5; HRMS (ESI-TOF) *m*/*z*: calcd for C_20_H_25_N_2_O_2_(M + H)^+^ 325.1916, found 325.1925

### 2.3. (9*H*-Fluoren-9-yl)Methyl 4-{1-Methyl-4-[1-Methyl-4-(1-Methyl-4-(1-Methyl-1*H*-Pyrrole-2-Carbonyl)Amino-1*H*-Pyrrole-2-Carbonyl)Amino-1H-Pyrrole-2-Carbonyl]Amino-1*H*-Pyrrole-2-Carbonyl}Aminobutylcarbamate (13)

Compounds **13**, **14,** and **15** were prepared according to the synthetic procedure of **12** [10–13].

Pyrrole amide tetramer **4** was prepared as previously described [13]. ^1^H-NMR (DMSO-*d*_6_): *δ* 9.96 (s, 1H, and CONH), 9.95 (s, 1H, and CONH), 9.85 (s, 1H, and CONH), 7.48 (d, 1H, *J* = 1.8 Hz, and Py-H), 7.25-7.24 (m, 2H, and Py-H × 2), 7.08 (d, 1H, *J* = 1.8 Hz, and Py-H), 7.06 (d, ^1^H, *J* = 1.8 Hz, and Py-H), 6.95-6.91, (m, 3H, and Py-H × 3), 6.06 (dd, ^1^H, *J* = 2.6 Hz, *J* = 3.8 Hz, and Py-H), 3.88 (s, 3H, and NCH_3_), 3.86 (s, 3H,and NCH_3_), 3.85 (s, 3H, and NCH_3_), 3.84 (s, 3H, and NCH_3_), and 3.74 (s, 3H, and OCH_3_); ^13^C-NMR (DMSO-*d*_6_): *δ* 161.0, 158.8, 158.7, 128.3, 125.6, 123.2, 122.9, 122.7, 122.5, 122.3, 121.0, 118.8, 118.7, 112.9, 108.6, 106.9, 105.0, 104.9, 51.2, 36.44, 36.38, 36.32, and 36.28; HRMS (ESI-TOF) *m*/*z*: calcd for C_25_H_28_N_7_O_5_(M + H)^+^ 506.2152, found 506.2151; UV (CH_3_OH): *λ*_max_ 306, 238 nm; *λ*_min_ 262, 223 nm; and *ε*_260_ 2.6 × 10^4^.

Pyrrole amide tetramer **4** (881 mg, 1.74 mmol) was dissolved in methanol (8.7 mL), and then 2 M NaOH aq. (8.7 mL) was added to the solution. After stirring at 60°C for 3 h, Dowex 50WX8 (H^+^-form) was added. Dowex 50WX8 was removed by filtration and the solution evaporated to give Py_4_-carboxylic acid (856 mg, quantitative yield), which was subsequently used without purification. ^1^H-NMR (DMSO-*d*_6_): *δ*12.12(s, 1H, COOH), 9.95 (s, 1H, and CONH), 9.91 (s, 1H, and CONH), 9.84 (s, 1H, and CONH), 7.43 (d, 1H, *J* = 1.9 Hz, and Py-H), 7.25-7.24 (m, 2H, and Py-H × 2), 7.07 (d, 1H, *J* = 1.8 Hz, and Py-H), 7.05 (d, 1H, *J* = 1.8 Hz, and Py-H), 6.95 (d, 1H, *J* = 2.2 Hz, and Py-H), 6.93-6.91 (m, 1H, and Py-H), 6.85 (d, 1H, *J* = 1.9 Hz, and Py-H), 6.06 (dd, 1H, *J* = 2.6 Hz, *J* =3.8 Hz, and Py-H), 3.88 (s, 3H, and NCH_3_), 3.86 (s, 3H, and NCH_3_), 3.85 (s, 3H, and NCH_3_), and 3.82 (s, 3H, and NCH_3_); ^13^C-NMR (DMSO-*d*_6_): *δ* 162.0, 158.6, 158.51, 158.47, 128.5, 125.5, 122.77, 122.71, 122.6, 122.3, 122.1, 120.3, 119.5, 118.5, 112.7, 108.4, 106.7, 104.77, 104.72, 36.24, 36.14, 36.12, and 36.08; HRMS (ESI-TOF) *m*/*z*: calculated for C_24_H_26_N_7_O_5_(M + H)^+^ 492.1995, found 492.1993.

Py_4_-carboxylic acid (492 mg, 1.00 mmol), **10** (520 mg, 1.50 mmol), and 1-hydroxybenzotriazole (HOBt) (270 mg, 2.00 mmol) were dissolved in DMF (5 mL), and then *N,N*′-dicyclohexylcarbodiimide (DCC) (310 mg, 1.50 mmol) and *N*-ethyldiisopropylamine (*N,N*-diisopropylethylamine: DIEA) (240 *μ*L, 1.38 mmol) were added to the solution. After stirring for 16 h, the precipitate was removed by filtration. The filtrate was diluted with chloroform (200 mL) and washed with 2 M HCl aq. (100 mL × 3), H_2_O (100 mL), 5% NaHCO_3_ aq. (100 mL × 3), and H_2_O (75 mL). The organic layer was dried over anhydrous magnesium sulfate and evaporated to dryness. The residue was subjected to chromatographic separation on a column of silica gel using a 0-5% methanol/chloroform solvent system to give **13** (555 mg, 71% yield) as a slightly brown glass. ^1^H-NMR (DMSO-*d*_6_): *δ* 9.93 (s, 1H, CONH), 9.88 (s, 1H, and CONH), 9.82 (s, 1H, and CONH), 8.00-7.97 (m, 1H, and CONH), 7.88 (d, 2H, *J* = 7.4 Hz, and Ar-H of the Fmoc group), 7.69 (d, 2H, *J* = 7.4 Hz, and Ar-H of the Fmoc group), 7.40 (t, 2H, *J* = 7.4 Hz, and Ar-H of the Fmoc group), 7.32 (t, 2H, *J* = 7.4 Hz, and Ar-H of the Fmoc group), 7.30-7.23 (m, 3H, CONHCH_2_, and Py-H × 2), 7.18 (d, 1H, *J* = 1.8 Hz, and Py-H), 7.06-7.04 (m, 2H, and Py-H x 2), 6.95-6.95 (m, 1H, and Py-H), 6.93-6.92 (m, 1H, and Py-H), 6.88-6.87 (m, 1H, and Py-H), 6.06 (dd, 1H, *J* = 2.5 Hz, *J* =3.9 Hz, and Py-H), 4.29 (d, 2H, *J* = 6.9 Hz, and CHCH_2_ of the Fmoc group), 4.22-4.19 (t, 1H, *J* = 6.9 Hz, and CHCH_2_ of the Fmoc group), 3.89 (s, 3H, and NCH_3_), 3.86 (s, 3H, and NCH_3_), 3.85 (s, 3H, and NCH_3_), 3.80 (s, 3H, and NCH_3_), 3.19-3.14 (m, 2H, and NHCH_2_), 3.03-2.98 (m, 2H, and NHCH_2_), and 1.52-1.39 (m, 4H, and CH_2_ × 2); ^13^C-NMR (DMSO-*d*_6_): *δ* 161.2, 158.6, 158.50, 158.46, 156.1, 143.9, 140.7, 128.1, 127.5, 127.0, 125.5, 125.1, 123.0, 122.8, 122.2, 122.11, 122.09, 120.1, 118.4, 117.7, 112.6, 106.6, 104.72, 104,69, 104.2, 65.2, 46.8, 38.1, 36.2, 36.1, 35.9, 27.0, and 26.7; HRMS (ESI-TOF) *m*/*z*: calculated for C_43_H_46_N_9_O_6_(M + H)^+^ 784.3571, found 784.3578.

### 2.4. (9*H*-Fluoren-9-yl)Methyl 5-{1-Methyl-4-[1-Methyl-4-(1-Methyl-4-(1-Methyl-1*H*-Pyrrole-2-Carbonyl)Amino-1*H*-Pyrrole-2-Carbonyl)Amino-1*H*-Pyrrole-2-Carbonyl-Amino-1*H*-Pyrrole-2-Carbonyl]Aminopentylcarbamate (14)

Py_4_-carboxylic acid (390 mg, 0.79 mmol), **11** (430 mg, 1.19 mmol), and 1-hydroxybenzotriazole (161 g, 1.19 mmol) were dissolved in DMF (4 mL), and then DCC (246 mg, 1.19 mmol) and *N*-ethyldiisopropylamine (190 *μ*L, 1.09 mmol) were added. After stirring for 18 h, the precipitate was removed by filtration. The filtrate was diluted with chloroform (200 mL) and washed with 2 M HCl aq. (100 mL × 3), H_2_O (100 mL), 5% NaHCO_3_ aq. (100 mL × 3), and H_2_O (75 mL). The organic layer was dried over anhydrous magnesium sulfate and evaporated to dryness. The residue was subjected to chromatographic separation on a column of silica gel using a 0-5% methanol/chloroform solvent system to give **14** (530 mg, 84% yield) as a slightly brown glass. ^1^H-NMR (DMSO-*d*_6_): *δ* 9.93 (s, 1H, and CONH), 9.88 (s, 1H, and CONH), 9.82 (s, 1H, and CONH), 7.98-7.95 (m, 1H, and CONH), 7.88 (d, 2H, *J* = 7.5 Hz, and Ar-H of the Fmoc group), 7.68 (d, 2H, *J* = 7.5 Hz, and Ar-H of the Fmoc group), 7.41 (t, 2H, *J* = 7.4 Hz, and Ar-H of the Fmoc group), 7.32 (t, 2H, *J* = 7.4 Hz, and Ar-H of the Fmoc group), 7.30-7.24 (m, 3H, CONH, and Py-H ×2), 7.18 (s, 1H, and Py-H), 7.05-7.04 (m, 2H, and Py-H × 2), 6.95-6.94 (m, 1H, and Py-H), 6.93-6.91 (m, 1H, and Py-H), 6.87 (s, 1H, and Py-H), 6.06 (dd, 1H, *J* = 2.6 Hz, *J* = 3.9 Hz, and Py-H), 4.29 (d, 2H, *J* = 6.9 Hz, and CHCH_2_ of the Fmoc group), 4.21 (t, 1H, *J* = 6.9 Hz, and CHCH_2_ of the Fmoc group), 3.89 (s, 3H, and NCH_3_), 3.86 (s, 3H, and NCH_3_), 3.85 (s, 3H, and NCH_3_), 3.80 (s, 3H, and NCH_3_), 3.18-3.13 (m, 2H, and NHCH_2_), 3.01-2.96 (m, 2H, and NHCH_2_), 1.52-1.39 (m, 4H, and CH_2_ × 2), and 1.31-1.24 (m, 2H, and CH_2_); ^13^C-NMR (DMSO-*d*_6_): *δ* 161.2, 158.6, 158.50, 158.46, 156.1, 143.9, 140.7, 128.9, 128.1, 127.6, 127.3, 127.0, 125.5, 125.1, 123.1, 122.8, 122.2, 122.1, 121.4, 120.1, 118.4, 117.7, 112.6, 106.6, 104.72, 104.68, 104.1, 65.1, 46.8, 38.3, 36.2, 36.1, 35.9, 29.11, 29.05, and 23.7; HRMS (ESI-TOF) *m*/*z*: calculated for C_44_H_48_N_9_O_6_(M + H)^+^ 798.3728, found 798.3733.

### 2.5. (9*H*-Fluoren-9-yl)Methyl 4-{1-Methyl-4-[1-Methyl-4-(1-Methyl-1*H*-Pyrrole-2-Carbonyl)Amino-1*H*-Pyrrole-2-Carbonyl]Amino-1*H*-Pyrrole-2-Carbonyl}Aminobutylcarbamate (15)

Pyrrole amide trimer **6** (515 mg, 1.34 mmol) was dissolved in methanol (6.7 mL), and then 2 M NaOH aq. (6.7 mL) was added to the solution. After stirring for 3 h at 60°C, Dowex 50WX8 (H^+^-form) was added. Dowex 50WX8 was removed by filtration and the solution evaporated to give the Py_3_-carboxylic acid (495 mg, quantitative yield), which was subsequently used without purification. ^1^H-NMR (DMSO-*d*_6_): *δ*12.16 (s, 1H, COOH), 9.93 (s, 1H, and CONH), 9.86 (s, 1H, and CONH), 7.46 (d, 1H, *J* = 1.9 Hz, and Py-H), 7.26 (d, 1H, *J* = 1.8 Hz, and Py-H), 7.08 (d, 1H, *J* = 1.8 Hz, and Py-H), 6.95-6.94 (m, 1H, and Py-H), 6.92 (dd, 1H, *J* = 1.7, and 3.9 Hz Py-H), 6.85 (d, 1H, *J* = 1.9 Hz, and Py-H), 6.06 (dd, 1H, *J* = 2.5, and 3.9 Hz Py-H), 3.92 (s, 3H, and NCH_3_), 3.89 (s, 3H, and NCH_3_), and 3.86 (s, 3H, and NCH_3_); ^13^C-NMR (DMSO-*d*_6_): *δ* 162.4, 159.1, 158.9, 128.6, 125.9, 123.2, 123.1, 122.6, 120.7, 120.0, 119.0, 113.1, 108.9, 107.1, 105.2, 36.7, 36.6, and 36.5; HRMS (ESI-TOF) *m*/*z*: calculated for C_18_H_20_N_5_O_4_(M + H)^+^ 370.1515, found 370.1509.

Py_3_-carboxylic acid (495 mg, 1.34 mmol), **10** (697 mg, 2.01 mmol), and 1-hydroxybenzotriazole (362 mg, 2.68 mmol) were dissolved in DMF (13.4 mL), and then DCC (553 mg, 2.68 mmol) and *N*-ethyldiisopropylamine (300 *μ*L, 1.74 mmol) were added. After stirring for 16 h, the precipitate was removed by filtration. The filtrate was diluted with chloroform (200 mL) and washed with 2 M HCl aq. (100 mL × 3), H_2_O (100 mL), 5% NaHCO_3_ aq. (100 mL × 3), and H_2_O (100 mL). The organic layer was dried over anhydrous magnesium sulfate and evaporated to dryness. The residue was subjected to chromatographic separation on a column of silica gel using a 0-5% methanol/chloroform solvent system to give **15** (538 mg, 61% yield) as a slightly brown glass. ^1^H-NMR (DMSO-*d*_6_): *δ* 9.87 (s, 1H, and CONH), 9.81 (s, 1H, and CONH), 7.99-7.97 (m, 1H, and CONH), 7.88 (d, 2H, *J* = 7.4 Hz, and Ar-H of the Fmoc group), 7.68 (d, 2H, *J* = 7.4 Hz, and Ar-H of the Fmoc group), 7.40 (t, 2H, *J* = 7.4 Hz, and Ar-H of the Fmoc group), 7.32 (t, 2H, *J* = 7.4 Hz, and Ar-H of the Fmoc group), 7.30-7.28 (m, 1H, and CONH), 7.23 (d, 1H, *J* = 1.8 Hz, and Py-H), 7.17 (d, 1H, *J* = 1.8 Hz, and Py-H), 7.03 (d, 1H, *J* = 1.8 Hz, and Py-H), 6.95-6.94 (m, 1H, and Py-H), 6.92-6.91 (m, 1H, and Py-H), 6.87 (d, 1H, *J* = 1.6 Hz, and Py-H), 6.06 (dd, 1H, *J* = 2.5, 3.8 Hz, and Py-H), 4.29 (d, 2H, *J* = 6.8 Hz, and CHCH_2_ of the Fmoc group), 4.20 (t, 1H, *J* = 6.8 Hz, and CHCH_2_ of the Fmoc group), 3.88 (s, 3H, and NCH_3_), 3.85 (s, 3H, and NCH_3_), 3.80 (s, 3H, and NCH_3_), 3.19-3.14 (m, 2H, and NHCH_2_), 3.02-2.98 (m, 2H, and NHCH_2_), and 1.53-1.37 (m, 4H, and CH_2_ × 2); ^13^C-NMR (DMSO-*d*_6_): *δ* 161.2, 158.6, 158.4, 156.1, 143.9, 140.7, 128.1, 127.6, 127.0, 125.5, 125.1, 123.0, 122.8, 122.09, 122.06, 120.1, 118.4, 117.7, 112.6, 106.6, 104.6, 104.1, 65.2, 46.8, 38.1, 36.2, 36.1, 35.9, 27.0, and 26.7; HRMS (ESI-TOF) *m*/*z*: calcd for C_37_H_40_N_7_O_5_(M + H)^+^ 662.3091, found 662.3054.

### 2.6. Ethyl 1-Methyl-4-Nitroimidazole-2-Carboxylate (21)

Ethyl 1-methylimidazole-2-carboxylate (**20**) [30] (2.97 g, 19.3 mmol) was dissolved in chloroform (19 mL), and then tetramethylammonium nitrate (5.26 g, 38.6 mmol) and trifluoroacetic anhydride (10.7 mL, 77.2 mmol) were added to the solution at 0°C. After stirring for 2.5 h at room temperature, 5% NaHCO_3_ aq. (300 mL) was added. Products were extracted with chloroform (900 mL) from the resulting solution. The organic layer was washed with 5% NaHCO_3_ aq. (200 mL × 2) and H_2_O (100 mL), dried over anhydrous magnesium sulfate, and evaporated to dryness. The residue was subjected to silica gel column chromatography using an ethyl acetate/hexane (3 : 5~1 : 1 *v*/*v*) solvent system to give **21** (2.28 g, 59% yield) and ethyl 1-methyl-5-nitroimidazole-2-carboxylate (**22**) (920 mg, 24% yield).

Compound **20**: Rf 0.20 (ethyl acetate/hexane (2 : 1 *v*/*v*) solvent system)

Compound **21** (white powder): Rf 0.42 (ethyl acetate/hexane (2 : 1 *v*/*v*) solvent system), ^1^H-NMR (DMSO-*d*_6_): *δ* 8.63 (s, 1H, and Im-H), 4.35 (q, 2H, *J* = 7.1 Hz, and CH_2_CH_3_), 3.99 (s, 3H, and NCH_3_), and 1.33 (t, 3H, *J* = 7.1 Hz, and CH_2_CH_3_); ^13^C-NMR (DMSO-*d*_6_): *δ* 157.7, 144.9, 134.8, 126.8, 61.7, 36.7, and 13.9; HRMS (ESI-TOF) *m*/*z*: calculated for C_7_H_10_N_3_O_4_(M + H)^+^ 200.0671, found 200.0664. Anal. Calcd for C_7_H_9_N_3_O_4_: C, 42.21; H, 4.55; N, 21.10, found. C, 42.23; H, 4.59; and N, 21.09

Compound **22** (white powder): Rf 0.49 (ethyl acetate/hexane (2 : 1 *v*/*v*) solvent system), ^1^H-NMR (DMSO-*d*_6_): *δ* 8.15 (s, 1H, and Im-H), 4.38 (q, 2H, *J* = 7.1 Hz, and OCH_2_CH_3_), 4.19 (s, 3H, and NCH_3_), and 1.33 (t, 3H, *J* = 7.1 Hz, and OCH_2_CH_3_); ^13^C-NMR (DMSO-*d*_6_): *δ* 158.0, 140.7, 138.9, 131.4, 62.1, 35.0, and 13.9; HRMS (ESI-TOF) *m*/*z*: calcd for C_7_H_10_N_3_O_4_(M + H)^+^ 200.0671, found 200.0669. Anal. Calcd for C_7_H_9_N_3_O_4_: C, 42.21; H, 4.55; N, 21.10, found. C, 42.13; H, 4.50; and N, 21.01

### 2.7. 1-Methylimidazole-2-Carboxylic Acid (23)

Ethyl 1-methyl-1*H*-imidazole-2-carboxylate (**20**) (3.08 g, 20.0 mmol) was dissolved in ethanol (50 mL)/pyridine (50 mL), and then 2 M NaOH aq. (100 mL) was added to the solution. After stirring at room temperature for 1 h, Dowex 50WX8 (H^+^-form) was added. Dowex 50WX8 was removed by filtration, and the solution evaporated to give **23** (2.52 g, quantitative yield) as a white powder, which was subsequently used without purification. ^1^H-NMR (CD_3_OD): *δ* 7.49 (s, 1H, and Im-H), 7.39 (s, 1H, and Im-H), and 4.15 (s, 3H, and NCH_3_); ^13^C-NMR (CD_3_OD): *δ* 158.0, 141.9, 126.6, 119.6, and 37.1; HRMS (ESI-TOF) *m*/*z*: calcd for C_5_H_7_N_2_O_2_(M + H)^+^ 127.0508, found 127.0512.

### 2.8. Ethyl 4-Amino-1-Methylimidazole-2-Carboxylate (24)

Ethyl 4-nitro-1*H*-imidazole-2-carboxylate (**21**) (2.54 g, 12.7 mmol) was dissolved in ethanol (64 mL)/ethyl acetate (64 mL), and then 10% Pd/C (0.49 g) was added. The mixture was stirred under a slight positive pressure of hydrogen at room temperature for 3 h. Pd/C was removed by filtration through celite and washed with ethyl acetate (50 mL). The filtrate was evaporated to dryness to give **24** (2.14 g, quantitative yield) as a white powder, which was subsequently used without purification. ^1^H-NMR (CDCl_3_): *δ* 6.37 (s, 1H, and Im-H), 4.38 (2H, q, *J* = 7.1 Hz, and OCH_2_CH_3_), 3.92 (3H, s, and NCH_3_), 2.96 (brs, NH_2_, and 2H), and 1.40 (3H, t, *J* = 7.1 Hz, and OCH_2_CH_3_); ^13^C-NMR (CDCl_3_): *δ* 159.0, 145.6, 131.6, 109.5, 61.3, 35.7, and 14.5; HRMS (ESI-TOF) *m*/*z*: calcd for C_7_H_12_N_3_O_2_(M + H)^+^ 170.0930, found 170.0938.

### 2.9. Ethyl 4-(*tert*-Butoxycarbonylamino)-1-Methyl-1*H*-Imidazole-2-Carboxylate (25)

Compound **24** (2.09 g, 12.4 mmol) was dissolved in DMF (15 mL), and then a solution of di-*tert*-butyldicarbonate (5.42 g, 24.8 mmol) in DMF (10 mL) was added. After stirring for 19 h at room temperature, H_2_O (50 mL) was added to the solution. The solution was diluted with chloroform (300 mL) and washed with H_2_O (100 mL × 3). The organic layer was dried over anhydrous magnesium sulfate and evaporated to dryness. The residue was subjected to silica gel column chromatography using an ethyl acetate/hexane (2 : 3 *v*/*v*) solvent system to give **25** (3.34 g, quantitative yield) as a white powder. ^1^H-NMR (DMSO-*d*_6_): *δ* 9.68 (s, 1H, and CONH), 7.30 (s, 1H, and Im-H), 4.25 (q, 2H, *J* = 7.2 Hz, and OCH_2_CH_3_), 3.88 (s, 3H, and NCH_3_), 1.44 (s, 9H, and OC(CH_3_)_3_), and 1.28 (t, 3H, *J* = 7.2 Hz, and OCH_2_CH_3_); ^13^C-NMR (DMSO-*d*_6_): *δ* 158.4, 152.7, 138.1, 130.9, 113.8, 78.9, 60.42, 35.4, 28.0, and 14.1; HRMS (ESI-TOF) *m*/*z*; calcd for C_12_H_20_N_3_O_4_(M + H)^+^ 270.1454, found 270.1439; Anal. Calcd for C_12_H_19_N_3_O_4_: C, 53.52; H, and 7.11; N, 15.60, found. C, 53.22; H, 7.05; and N, 15.55.

### 2.10. 4-(*tert*-Butoxycarbonylamino)-1-Methyl-1*H*-Imidazole-2-Carboxylic Acid (26)

Compound **25** (2.63 g, 9.80 mmol) was dissolved in ethanol (24.5 mL)/pyridine (24.5 mL), and then 2 M NaOH aq. (49 mL) was added to the solution. After stirring at room temperature for 1 h, Dowex 50WX8 (H^+^-form) was added. Dowex 50WX8 was removed by filtration, and the solution evaporated to give **26** (2.36 g, quantitative yield) as a white powder, which was subsequently used without purification. ^1^H-NMR (DMSO-*d*_6_): *δ* 9.52 (s, 1H, and CONH), 7.16 (s, 1H, and Im-H), 3.88 (s, 3H, and NCH_3_), and 1.44 (s, 9H, and OC(CH_3_)_3_); ^13^C-NMR (DMSO-*d*_6_): *δ* 160.4, 152.5, 137.1, 133.7, 112.2, 79.0, 35.3, and 28.1; HRMS (ESI-TOF) *m*/*z*: calcd for C_10_H_15_N_3_O_4_Na (M + Na)^+^ 264.0960, found 264.0950.

### 2.11. Ethyl 4-(4-(*tert*-Butoxycarbonylamino)-1-Methyl-1*H*-Imidazole-2-Carboxamido)-1-Methyl-1*H*-Imidazole-2-Carboxylate (27)

Compounds **24** (660 mg, 3.90 mmol) and **26** (1.13 g, 4.70 mmol) were dissolved in DMF (39 mL), and then *N*-ethyldiisopropylamine (1.36 mL, 7.80 mmol), 1-hydroxybenzotriazole (1.05 g, 7.80 mmol), and *N,N*′-diisopropylcarbodiimide (DCI) (3.60 mL, 23.3 mmol) were added to the solution. After stirring for 18 h, the reaction solution was diluted with chloroform (200 mL) and washed with 5% NaHCO_3_ aq. (100 mL × 2) and H_2_O (100 mL). The organic layer was dried over anhydrous magnesium sulfate and evaporated to dryness. The residue was subjected to chromatographic separation on a column of silica gel using an ethyl acetate/hexane (2 : 1 *v*/*v*) solvent system to give **27** (1.34 g, 88% yield) as a slightly brown glass. ^1^H-NMR (CDCl_3_): *δ* 9.45 (s, 1H, and CONH), 7.54 (s, 1H, and Im-H), 7.25 (s, 1H, and Im-H), 6.71 (s, 1H, and CONH), 4.43 (q, 2H, *J* = 7.2 Hz, and OCH_2_CH_3_), 4.025 (s, 3H, and NCH_3_), 4.016 (s, 3H, and NCH_3_), 1.52 (s, 9H, and OC(CH_3_)_3_), 1.44 (t, 3H, *J* = 7.2 Hz, and OCH_2_CH_3_); ^13^C-NMR (CDCl_3_): *δ* 159.0, 156.2, 152.6, 137.2, 136.8, 133.2, 132.0, 114.9, 112.7, 81.1, 61.7, 36.2, 35.7, 28.5, and 14.6; HRMS (ESI-TOF) *m*/*z*: calcd for C_17_H_25_N_6_O_5_(M + H)^+^ 393.1886, found 393.1902.

### 2.12. Ethyl 1-Methyl-4-(1-Methyl-4-(1-Methyl-1*H*-Imidazole-2-Carboxamido)-1*H*-Imidazole-2-Carboxamido)-1*H*-Imidazole-2-Carboxylate (28)

Compound **27** (510 mg, 1.30 mmol) was dissolved in ethanol (19.5 mL)/chloroform (6.5 mL), and then acetyl chloride (2.80 mL, 39.0 mmol) was added to the solution at room temperature. After stirring for 2 h at 40°C, the reaction solution was concentrated *in vacuo* to give Im_2_-amine compound, which was subsequently used without purification. ^1^H-NMR (DMSO-*d*_6_): *δ* 10.45 (1H, s, and CONH), 7.71 (s, 1H, and Im-H), 7.47 (s, and 1H, Im-H), 4.28 (q, 2H, *J* = 7.1 Hz, and OCH_2_CH_3_), 3.99 (s, 3H, and NCH_3_), 3.94 (s, 3H, and NCH_3_), and 1.29 (t, 3H, *J* = 7.1 Hz, and OCH_2_CH_3_); ^13^C-NMR (DMSO-*d*_6_): *δ* 158.1, 154.9, 135.7, 135.0, 131.4, 129.9, 117.6, 115.9, 60.8, 48.6, 35.75, 35.66, and 14.1; HRMS (ESI-TOF) *m*/*z*: calcd for C_12_H_17_N_6_O_3_(M + H)^+^ 293.1362, found 293.1365.

Im_2_-amine compound (1.30 mmol) and **23** (214 mg, 1.70 mmol) were dissolved in DMF (13 mL), and then EDCI (1.02 g, 5.30 mmol) and DMAP (490 mg, 4.00 mmol) were added to the solution. After stirring at room temperature for 18 h, the solution was diluted with chloroform (200 mL) and washed with H_2_O (10 mL), 5% NaHCO_3_ aq. (100 mL × 2) and H_2_O (100 mL). The organic layer was dried over anhydrous magnesium sulfate and evaporated to dryness. The residue was subjected to chromatographic separation on a column of silica gel using an ethyl acetate/hexane (2 : 1 *v*/*v*) solvent system to give **28** (440 mg, 84% yield) as a slightly brown glass. ^1^H-NMR (CDCl_3_): *δ* 9.48 (s, 1H, and CONH), 9.40 (s, 1H, and CONH), 7.56 (s, 1H, and Im-H), 7.50 (s, 1H, and Im-H), 7.11 (s, 1H, and Im-H), 7.02 (s, 1H, and Im-H), 4.44 (q, 2H, *J* = 7.1 Hz, and OCH_2_CH_3_), 4.10 (s, 3H, and NCH_3_), 4.08 (s, 3H, and NCH_3_), 4.03 (s, 3H, and NCH_3_), and 1.45 (t, 3H, and *J* = 7.1 Hz, and OCH_2_CH_3_); ^13^C-NMR (CDCl_3_): *δ* 158.1, 156.5, 156.2, 138.5, 136.8, 136.0, 133.6, 132.1, 128.4, 126.4, 115.1, 114.6, 61.8, 36.2, 35.90, 35.85, and 14.6; HRMS (ESI-TOF) *m*/*z*: calcd for C_17_H_21_N_8_O_4_(M + H)^+^ 401.1686, found 401.1682; UV (CH_3_OH): *λ*_max_ 311 nm, *λ*_min_ 236 nm, *ε*_260_ 1.2 × 10^4^.

### 2.13. (9*H*-Fluoren-9-yl)Methyl 3-(1-Methyl-4-(1-Methyl-4-(1-Methyl-1*H*-Imidazole-2-Carboxamido)-1*H*-Imidazole-2-Carboxamido)-1*H*-Imidazole-2-Carboxamido)Propylcarbamate (29)

Compound **28** (500 mg, 1.20 mmol) was dissolved in ethanol (6 mL)/pyridine (6 mL) and then 2 M NaOH aq. (12 mL) was added to the solution. After stirring at room temperature for 3 h, Dowex 50WX8 (H^+^-form) was added. Dowex 50WX8 was removed by filtration, and the solution evaporated to give Im_3_-carboxylic acid (450 mg, quantitative yield) as a white powder, which was subsequently used without purification. ^1^H-NMR (DMSO-*d*_6_): *δ* 10.09 (s, 1H, and CONH), 9.90 (s, 1H, and CONH), 7.61 (s, 1H, and Im-H), 7.55 (s, 1H, and Im-H), 7.44 (s, 1H, and Im-H), 7.07 (s, 1H, and Im-H), 4.01 (s, 3H, and NCH_3_), 4.00 (s, 3H, and NCH_3_), and 3.94 (s, 3H, and NCH_3_); ^13^C-NMR (DMSO-*d*_6_): *δ* 160.2, 155.9, 155.4, 137.8, 135.3, 135.0, 134.3, 133.4, 127.7, 127.0, 114.6, 113.8, 35.4, 35.2, and 35.1; HRMS (ESI-TOF) *m*/*z*: calcd for C_15_H_17_N_8_O_4_(M + H)^+^ 373.1373, found 373.1366.

Im_3_-carboxylic acid (330 mg, 0.90 mmol) and **9** (440 mg, 1.30 mmol) were dissolved in DMF (9 mL), and then *N*-ethyldiisopropylamine (210 *μ*L, 1.20 mmol), 1-hydroxybenzotriazole (360 mg, 2.70 mmol), and *N*,*N*′-diisopropylcarbodiimide (800 *μ*L, 5.40 mmol) were added to the solution. After stirring for 19 h, the solution was diluted with chloroform (100 mL) and washed with H_2_O (50 mL × 3). The organic layer was dried over anhydrous magnesium sulfate and evaporated to dryness. The residue was subjected to chromatographic separation on a column of silica gel using a 0-5% methanol/chloroform solvent system to give **29** (290 mg, 50% yield) as a slightly brown glass. ^1^H-NMR (DMSO-*d*_6_): *δ* 10.05 (s, 1H, and CONH), 9.56 (s, 1H, and CONH), 8.31-8.29 (m, and 1H, CONH), 7.88 (d, 2H, *J* = 7.4 Hz, and Ar − H × 2 of the Fmoc group), 7.69 (d, 2H, *J* = 7.4 Hz, Ar − H × 2 of the Fmoc group), 7.64 (s, 1H, and Im-H), 7.51 (s, 1H, and Im-H), 7.45 (s, 1H, and Im-H), 7.42 (t, 2H, *J* = 7.4 Hz, and Ar-H × 2 of the Fmoc group), 7.34 (t, 2H, *J* = 7.4 Hz, and Ar − H × 2 of the Fmoc group), 7.30-7.27 (m, 1H, and CONH), 7.08 (s, 1H, and Im-H), 4.32 (d, 2H, *J* = 6.9 Hz, and CHCH_2_ of the Fmoc group), 4.22 (t, 1H, *J* = 6.9 Hz, and CHCH_2_ of the Fmoc group), 4.02 (s, 3H, and NCH_3_), 4.00 (s, 3H, and NCH_3_), 3.96 (s, 3H, and NCH_3_), 3.24-3.20 (m, 2H, and NHCH_2_), 3.05-3.01 (m, 2H, and NHCH_2_), and 1.66-1.59 (m, 2H, and CH_2_); ^13^C-NMR (CDCl_3_): *δ* 159.7, 156.9, 156.5, 156.0, 144.2, 141.4, 138.5, 135.9, 135.5, 134.4, 133.8, 128.3, 127.7, 127.1, 126.3, 125.2, 120.1, 114.6, 113.9, 66.7, 47.5, 38.0, 36.0, 35.81, 35.78, 35.76, and 30.2; HRMS (ESI-TOF) *m*/*z*: calcd for C_33_H_35_N_10_O_5_(M + H)^+^ 651.2792, found 651.2820.

### 2.14. (9*H*-Fluoren-9-yl)Methyl 4-(1-Methyl-4-(1-Methyl-4-(1-Methyl-1*H*-Imidazole-2-Carboxamido)-1*H*-Imidazole-2-Carboxamido)-1*H*-Imidazole-2-Carboxamido)Butylcarbamate (30)

Im_3_-carboxylic acid (740 mg, 2.00 mmol) and **10** (1.04 g, 3.00 mmol) were dissolved in DMF (20 mL), and then *N*-ethyldiisopropylamine (400 *μ*L, 2.60 mmol), 1-hydroxybenzotriazole (810 mg, 6.00 mmol), and *N*,*N*′-diisopropylcarbodiimide (1.80 mL, 12.0 mmol) were added to the solution. After stirring for 19 h, the solution was diluted with chloroform (200 mL) and washed with H_2_O (100 *mL* × 3). The organic layer was dried over anhydrous magnesium sulfate and evaporated to dryness. The residue was subjected to chromatographic separation on a column of silica gel using a 0-5% methanol/chloroform solvent system to give **30** (430 mg, 33% yield) as a slightly brown glass. ^1^H-NMR (DMSO-*d*_6_): *δ* 9.68 (s, 1H, and CONH), 9.56 (s, 1H, and CONH), 8.28-8.26 (m, 1H, and CONH), 7.88 (d, 2H, *J* = 7.5 Hz, and Ar-H of the Fmoc group), 7.68 (d, 2H, *J* = 7.5 Hz, and Ar-H of the Fmoc group), 7.64 (s, 1H, and Im-H), 7.51 (s, 1H, and Im-H), 7.45 (s, 1H, and Im-H), 7.39 (t, 2H, *J* = 7.5 Hz, and Ar-H of the Fmoc group), 7.33 (t, 2H, *J* = 7.5 Hz, and Ar-H of the Fmoc group), 7.29-7.297 (m, 1H, and CONH), 7.08 (s, 1H, and Im-H), 4.29 (d, 2H, *J* = 6.8 Hz, and CHCH*_2_* of the Fmoc group), 4.20 (t, 1H, *J* = 6.8 Hz, and CHCH_2_ of the Fmoc group), 4.02 (s, 3H, and NCH_3_), 4.00 (s, 3H, and NCH_3_), 3.95 (s, 3H, and NCH_3_), 3.24-3.19 (m, 2H, and NHCH_2_), 3.02-2.98 (m, 2H, and NHCH_2_), and 1.49-1.41 (m, 4H, and CH_2_ × 2); ^13^C-NMR (CDCl_3_): *δ* 159.3, 156.7, 156.5, 156.0, 144.2, 141.5, 138.5, 135.9, 135.4, 134.6, 133.7, 128.4, 127.8, 127.2, 126.3, 125.2, 120.1, 114.5, 113.8, 66.7, 47.5, 40.9, 38.8, 35.86, 35.83, 35.80, 27.5, and 27.2; HRMS (ESI-TOF) *m*/*z*: calcd for C_34_H_37_N_10_O_5_(M + H)^+^ 665.2948, found 665.2973.

### 2.15. (9*H*-Fluoren-9-yl)Methyl 5-(1-Methyl-4-(1-Methyl-4-(1-Methyl-1*H*-Imidazole-2-Carboxamido)-1*H*-Imidazole-2-Carboxamido)-1*H*-Imidazole-2-Carboxamido)Pentylcarbamate (31)

Im_3_-carboxylic acid (450 mg, 1.20 mmol) and **11** (650 mg, 1.80 mmol) were dissolved in DMF (12 mL), and then *N*-ethyldiisopropylamine (300 *μ*L, 1.60 mmol), 1-hydroxybenzotriazole (490 mg, 3.60 mmol) and *N*,*N*′-diisopropylcarbodiimide (1.10 mL, 7.20 mmol) were added to the solution. After stirring for 19 h, the solution was diluted with chloroform (100 mL) and washed with H_2_O (50 mL × 3). The organic layer was dried over anhydrous magnesium sulfate and evaporated to dryness. The residue was subjected to chromatographic separation on a column of silica gel using a 0-5% methanol/chloroform solvent system to give **31** (580 mg, 71% yield) as a slightly brown glass. ^1^H-NMR (DMSO-*d*_6_): *δ* 10.03 (s, 1H, and CONH), 9.56 (s, 1H, and CONH), 8.26-8.24 (m, 1H, and CONH), 7.87 (d, 2H, *J* = 7.4 Hz, and Ar-H of the Fmoc group), 7.67 (d, 2H, *J* = 7.4 Hz, and Ar-H of the Fmoc group), 7.64 (s, 1H, and Im-H), 7.50 (s, 1H, and Im-H), 7.45 (s, 1H, and Im-H), 7.39 (t, 2H, *J* = 7.5 Hz, and Ar-H of the Fmoc group), 7.33 (t, 2H, *J* = 7.5 Hz, and Ar-H of the Fmoc group), 7.26-7.24 (m, 1H, and CONH), 7.08 (s, 1H, and Im-H), 4.29 (d, 2H, *J* = 6.9 Hz, and CHCH_2_ of the Fmoc group), 4.19 (t, 1H, *J* = 6.9 Hz, and CHCH_2_ of the Fmoc group), 4.01 (s, 3H, and NCH_3_), 4.00 (s, 3H, and NCH_3_), 3.95 (s, 3H, and NCH_3_), 3.23-3.18 (m, 2H, and NHCH_2_), 3.00-2.95 (m, 2H, and NHCH_2_), 1.54-1.39 (m, 4H, and CH_2_ × 2), and 1.30-1.24 (m, 2H, and CH_2_); ^13^C-NMR(CDCl_3_): *δ* 159.2, 156.6, 156.3, 155.9, 144.1, 141.3, 138.4, 135.8, 135.3, 134.6, 133.7, 128.2, 127.6, 127.0, 126.1, 125.1, 120.0, 114.4, 113.7, 66.5, 47.4, 40.9, 38.8, 35.69, 35.66, 35.64, 29.6, 29.4, and 24.1; HRMS (ESI-TOF) *m*/*z*: calcd for C_35_H_39_N_10_O_5_(M + H)^+^ 679.3105, found 679.3085.

### 2.16. Ethyl 4-(1-Methyl-4-(1-Methyl-4-(1-Methyl-1*H*-Pyrrole-2-Carboxamido)-1*H*-Pyrrole-2-Carboxamido)-1*H*-Pyrrole-2-Carboxamido)Butanoate (32)

Pyrrole amide trimer **6** (1.51 g, 3.93 mmol) was dissolved in ethanol (17 mL)/pyridine (17 mL), and then 2 M NaOH aq. (34 mL) was added to the solution. After stirring at room temperature for 3 h, Dowex 50WX8 (H^+^-form) was added. Dowex 50WX8 was removed by filtration, and the solution evaporated to give Py_3_-carboxylic acid (1.45 g, quantitative yield), which was subsequently used without purification.

Py_3_-carboxylic acid (1.45 g, 3.93 mmol) and ethyl 4-aminobutanoate (0.774 g, 5.90 mmol) were dissolved in dichloromethane (20 mL), and then EDCI (1.50 g, 7.86 mmol) and DMAP (960 mg, 7.86 mmol) were added to the solution. After stirring at room temperature for 10 h, the solution was diluted with chloroform (300 mL) and washed with H_2_O (80 mL), 5% NaHCO_3_ aq. (80 mL × 2), and H_2_O (80 mL). The organic layer was dried over anhydrous magnesium sulfate and evaporated to dryness. The residue was subjected to chromatographic separation on a column of silica gel using an ethyl acetate/hexane (2 : 1~8 : 1 *v*/*v*) solvent system to give **32** (1.56 g, 82% yield) as a slightly brown glass. ^1^H-NMR(CDCl_3_): *δ* 7.60 (s, 1H, and CONH), 7.50 (s, 1H, and CONH), 7.15 (d, 1H, *J* = 1.8 Hz, and Py-H), 7.12 (d, 1H, *J* = 1.8 Hz, and Py-H), 6.78-6.77 (m, 1H, and Py-H), 6.71 (d, 1H, *J* = 1.8 Hz, Py-H), 6.68-6.66 (m, 1H, and Py-H), 6.52 (d, 1H, *J* = 1.9 Hz, and Py-H), 6.24-6,22 (m, 1H, and CONH), 6.13 (dd, 1H, *J* = 2.6 Hz, *J* =4.0 Hz, and Py-H), 4.15 (q, 2H, *J* = 7.2 Hz, and OCH_2_CH_3_), 3.98 (s, 3H, and NCH_3_), 3.93 (s, 3H, and NCH_3_), 3.90 (s, 3H, and NCH_3_), 3.44-3.39 (m, 2H, and NHCH_2_CH_2_-), 2.41 (t, 2H, *J* = 7.1 Hz, and COCH_2_CH_2_-), 1.95-1.88 (m, 2H, and -CH_2_CH_2_CH_2_-), and 1.25 (t, 3H, *J* = 7.2 Hz, and OCH_2_CH_3_); ^13^C-NMR(CDCl_3_): *δ* 173.8, 161.8, 159.5, 1589.0, 128.5, 125.4, 123.4, 123.2, 121.5, 121.2, 119.3, 118.9, 112.0, 107.4, 103.8, 103.2, 60.7, 38.9, 36.8, 36.65, 36.57, 31.9, 24.7, and 14.2; HRMS (ESI-TOF) *m*/*z*: calcd for C_24_H_31_N_6_O_5_ (M + H)^+^ 483.2356, found 483.2354.

### 2.17. Ethyl 1-Methyl-4-(1-Methyl-4-(1-Methyl-4-(*tert*-Butoxycarbonylamino)-1*H*-Imidazole-2-Carboxamido)-1*H*-Imidazole-2-Carboxamido)-1*H*-Imidazole-2-Carboxylate (33)

Compound **27** (2.22 g, 5.65 mmol) was dissolved in ethanol (19.5 mL)/chloroform (6.5 mL), and then acetyl chloride (2.80 mL, 39.0 mmol) was added to the solution at room temperature. After stirring for 2 h at 40°C, the reaction solution was concentrated *in vacuo* to give Im_2_-amine compound, which was subsequently used without purification.

Im_2_-amine compound (5.65 mmol) and **26** (2.27 g, 8.48 mmol) were dissolved in dichloromethane (56.5 mL), and then EDCI (3.25 g, 17.0 mmol) and DMAP (2.07 g, 17.0 mmol) were added to the solution. After stirring at room temperature for 19 h, the solution was diluted with chloroform (500 mL) and washed with H_2_O (100 mL), 5% NaHCO_3_ aq. (100 mL × 2) and H_2_O (100 mL). The organic layer was dried over anhydrous magnesium sulfate and evaporated to dryness. The residue was subjected to chromatographic separation on a column of silica gel using an ethyl acetate/hexane (2 : 1 *v*/*v*) solvent system to give **33** (2.05 g, 70% yield) as a slightly brown glass. ^1^H-NMR (CDCl_3_): *δ* 9.53 (s, 1H, and CONH), 9.17 (s, 1H, and CONH), 7.57 (s, 1H, and Im-H), 7.49 (s, 1H, and Im-H), 7.19 (s, 1H, and Im-H), 6.92 (s, 1H, and CONH), 4.44 (q, 2H, *J* = 7.1 Hz, and OCH_2_CH_3_), 4.07 (s, 3H, and NCH_3_), 4.05 (s, 3H, and NCH_3_), 4.03 (s, 3H, and NCH_3_), 1.53 (s, 9H, and OC(CH_3_)_3_), 1.45 (t, 3H, *J* = 7.1 Hz, and OCH_2_CH_3_); ^13^C-NMR (CDCl_3_): *δ* 158.8, 155.9, 155.8, 152.5, 137.0, 136.6, 135.7, 133.3, 133.0, 131.8, 114.8, 114.3, 112.7, 80.8, 61.5, 36.0, 35.7, 35.6, 28.3, and 14.3; HRMS (ESI-TOF) *m*/*z*: calcd for C_22_H_30_N_9_O_6_(M + H)^+^ 516.2319, found 516.2318.

### 2.18. Ethyl 1-Methyl-4-(1-Methyl-4-(1-Methyl-4-(4-(1-Methyl-4-(1-Methyl-4-(1-Methyl-1*H*-Pyrrole-2-Carboxamido)-1*H*-Pyrrole-2-Carboxamido)-1*H*-Pyrrole-2-Carboxamido)Butanamido)-1*H*-Imidazole-2-Carboxamido)-1*H*-Imidazole-2-Carboxamido)-1*H*-Imidazole-2-Carboxylate (34)

Compound **32** (1.44 g, 2.99 mmol) was dissolved in ethanol (7.5 mL)/pyridine (7.5 mL), and then 2 M NaOH aq. (15 mL) was added to the solution. After stirring at room temperature for 3 h, Dowex 50WX8 (H^+^-form) was added. Dowex 50WX8 was removed by filtration and the solution evaporated to give Py_3_-NH(CH_2_)_3_CO_2_H (1.36 g, quantitative yield), which was subsequently used without purification. ^1^H-NMR (DMSO-*d*_6_): *δ* 9.89 (s, 1H, and CONH), 9.83 (s, 1H, and CONH), 8.06-8.03 (m, 1H, and CONH), 7.23 (d, 1H, *J* = 1.8 Hz, and Py-H), 7.18 (d, 1H, *J* = 1.8 Hz, and Py-H), 7.02 (d, 1H, *J* = 1.9 Hz, and Py-H), 6.95-6.93 (m, 1H, and Py-H), 6.92-6.91 (m, 2H, Py − H × 2), 6.87 (d, 1H, *J* = 1.9 Hz, and Py-H), 6.06 (dd, 1H, *J* = 2.6 Hz, *J* = 3.9 Hz, and Py-H), 3.88 (s, 3H, and NCH_3_), 3.85 (s, 3H, and NCH_3_), 3.79 (s, 3H, and NCH_3_), 3.19-3.16 (m, 2H, and NHCH_2_CH_2_-), 2.24 (t, 2H, *J* = 7.4 Hz, and COCH_2_CH_2_-), and 1.72-1.69 (m, 2H, and -CH_2_CH_2_CH_2_-); ^13^C-NMR(DMSO-*d*_6_): *δ* 174.5, 161.4, 158.6, 158.5, 128.2, 125.5, 123.0, 122.8, 122.15, 122.12, 118.5, 117.8, 112.7, 106.7, 104.7, 104.2, 37.9, 36.3, 36.1, 36.0, 31.3, and 24.8; HRMS (ESI-TOF) *m*/*z*: calcd for C_22_H_27_N_6_O_5_(M + H)^+^ 455.2043, found 455.2039.

Compound **33** (54 mg, 0.104 mmol) was dissolved in ethanol (1.56 mL)/chloroform (0.52 mL), and then acetyl chloride (220 *μ*L, 3.14 mmol) was added to the solution at room temperature. After stirring for 3 h at 40°C, the reaction solution was concentrated *in vacuo* to give Im_3_-amine compound, which was subsequently used without purification. ^1^H-NMR (CD_3_OD): *δ* 7.81 (s, 1H, and Im-H), 7.65 (s, 1H, and Im-H), 7.35 (s, 1H, and Im-H), 4.49 (q, 2H, *J* = 7.1 Hz, and OCH_2_CH_3_), 4.14 (s, 3H, and NCH_3_), 4.11 (s, 3H, and NCH_3_), 4.10 (s, 3H, and NCH_3_), and 1.45 (t, 3H, *J* = 7.1 Hz, and OCH_2_CH_3_); ^13^C-NMR (DMSO-*d*_6_): *δ* 158.2, 155.6, 154.7, 135.9, 135.2, 134.8, 133.5, 131.5, 131.4, 115.9, 115.5, 115.1, 60.8, 35.7, 35.7, 35.2, and 14.1; HRMS (ESI-TOF) *m*/*z*: calcd for C_17_H_21_N_9_O_4_(M + H)^+^ 416.1795, found 416.1788.

Im_3_-amine compound (0.104 mmol) and Py_3_-NH(CH_2_)_3_CO_2_H (71 mg, 0.156 mmol) were dissolved in dichloromethane (2.1 mL), and then EDCI (60 mg, 0.312 mmol) and DMAP (38 mg, 0.312 mmol) were added to the solution. After stirring at room temperature for 14 h, the solution was diluted with chloroform (50 mL) and washed with H_2_O (20 mL), 5% NaHCO_3_ aq. (20 mL × 2) and H_2_O (20 mL). The organic layer was dried over anhydrous magnesium sulfate and evaporated to dryness. The residue was subjected to chromatographic separation on a column of silica gel using a 2% methanol/chloroform solvent system to give **34** (50 mg, 57% yield) as a slightly brown glass. ^1^H-NMR(CDCl_3_): *δ* 9.51 (s, 1H, and CONH), 9.24 (s, 1H, and CONH), 8.87 (s, 1H, and CONH), 7.93 (s, 1H, and CONH), 7.58-7.54 (m, 2H, Im-H,and CONH), 7.46 (s, 1H, and Im-H), 7.44 (s, 1H, and Im-H), 7.20 (d, 1H, *J* = 1.5 Hz, and Py-H), 7.18 (d, 1H, *J* = 1.5 Hz, and Py-H), 6.75-6.74 (m, 2H, and Py − H × 2), 6.60-6.59 (m, 1H, and Py-H), 6.52-6.44 (m, 2H, Py-H, and CONH), 6.10-6.09 (m, 1H, and Py-H), 4.39 (q, 2H, *J* = 7.1 Hz, and OCH_2_CH_3_), 4.02 (s, 3H, and NCH_3_), 4.01 (s, 3H, and NCH_3_), 4.00 (s, 3H, and NCH_3_), 3.97 (s, 3H, and NCH_3_), 3.93 (s, 3H, and NCH_3_), 3.92 (s, 3H, and NCH_3_), 3.52-3.48 (m, 2H, and NHCH_2_CH_2_-), 2.52 (t, 2H, *J* = 6.7 Hz, and COCH_2_CH_2_-), 2.07-2.04 (m, 2H, -CH_2_CH_2_CH_2_-), and 1.40 (t, 3H, *J* = 7.1 Hz, and OCH_2_CH_3_); ^13^C-NMR(CDCl_3_): *δ* 171.2, 162.6, 159.8, 159.3, 158.7, 155.9, 155.8, 136.5, 136.4, 135.8, 135.7, 133.3, 133.1, 131.9, 128.4, 125.5, 123.09, 123.05, 121.9, 121.7, 119.4, 119.0, 115.0, 114.6, 112.5, 107.3, 104.2, 103.9, 61.6, 38.9, 36.8, 36.6, 36.5, 36.1, 35.63, 35.56, 33.8, 25.5, and 14.2; HRMS (ESI-TOF) *m*/*z*: calcd for C_39_H_46_N_15_O_8_(M + H)^+^ 852.3654, found 852.3651; UV (CH_3_OH): *λ*_max_ 300 nm, *λ*_min_ 257 nm, *ε*_260_ 3.0 × 10^4^.

### 2.19. (9*H*-Fluoren-9-yl)Methyl-(3-(1-Methyl-4-(1-Methyl-4-(1-Methyl-4-(4-(1-Methyl-4-(1-Methyl-4-(1-Methyl-1*H*-Pyrrole-2-Carboxamido)-1*H*-Pyrrole-2-Carboxamido)-1*H*-Pyrrole-2-Carboxamido)Butanamido)-1*H*-Imidazole-2-Carboxamido)-1*H*-Imidazole-2-Carboxamido)-1*H*-Imidazole-2-Carboxamido)Ethyl)Carbamate (35)

Compound **34** (350 mg, 0.41 mmol) was dissolved in ethanol (2 mL)/pyridine (2 mL), and then 2 M NaOH aq. (2 mL) was added to the solution. After stirring at room temperature for 6 h, Dowex 50WX8 (H^+^-form) was added. Dowex 50WX8 was removed by filtration, and the solution evaporated to give Py_3_-*γ*-Im_3_-carboxylic acid (338 mg, quantitative yield) as a white powder, which was subsequently used without purification. ^1^H-NMR(DMSO-*d*_6_): *δ* 10.48 (s, 1H, and CONH), 10.22 (s, 1H, and CONH), 9.93 (s, 1H, and CONH), 9.89 (s, 1H, and CONH), 9.47 (s, 1H, and CONH), 8.09-8.06 (m, 1H, and CONH), 7.62 (s, 1H, and Im-H), 7.60 (s, 1H, and Im-H), 7.54 (s, 1H, and Im-H), 7.24 (d, 1H, *J* = 1.5 Hz, and Py-H), 7.19 (d, 1H, *J* = 1.5 Hz, and Py-H), 7.06 (d, 1H, *J* = 1.7 Hz, and Py-H), 6.97-6.95 (m, 1H, and Py-H), 6.94-6.93 (m, 1H, and Py-H), 6.91 (d, 1H, *J* = 1.7 Hz, and Py-H), 6.05-6.04 (m, 1H, and Py-H), 4.01 (s, 3H, and NCH_3_), 3.98 (s, 3H, and NCH_3_), 3.94 (s, 3H, and NCH_3_), 3.88 (s, 3H, and NCH_3_), 3.84 (s, 3H, and NCH_3_), 3.80 (s, 3H, and NCH_3_), 3.22-3.16 (m, 2H, and NHCH_2_CH_2_-), 2.39 (t, 2H, *J* = 7.2 Hz, and COCH_2_CH_2_-), and 1.82-1.79 (m, 2H, and -CH_2_CH_2_CH_2_-); ^13^C-NMR(DMSO-*d*_6_): *δ* 169.6, 160.8, 158.1, 158.0, 155.0, 154.9, 149.1, 136.0, 135.1, 134.5, 133.0, 132.9, 132.1, 127.6, 125.0, 123.4, 122.5, 122.3, 121.7, 121.6, 117.9, 117.3, 114.3, 114.0, 112.3, 106.1, 104.3, 103.8, 37.6, 35.8, 35.6, 35.5, 35.1, 34.8, 34.6, 32.4, and 25.0; HRMS (ESI-TOF) *m*/*z*: calcd for C_37_H_42_N_15_O_8_(M + H)^+^ 824.3341, found 824.3337.

Py_3_-*γ*-Im_3_-carboxylic acid (330 mg, 0.40 mmol) and **8** (190 mg, 0.60 mmol) were dissolved in dichloromethane (6 mL), and then EDCI (230 mg, 1.20 mmol) and DMAP (70 mg, 0.60 mmol) were added to the solution. After stirring at room temperature for 17 h, the solution was diluted with chloroform (50 mL) and washed with H_2_O (20 mL), 5% NaHCO_3_ aq. (20 mL × 2), and H_2_O (20 mL). The organic layer was dried over anhydrous magnesium sulfate and evaporated to dryness. The residue was subjected to chromatographic separation on a column of silica gel using a 0-5% methanol/chloroform solvent system to give **35** (240 mg, 55% yield) as a slightly brown glass. ^1^H-NMR (DMSO-*d*_6_): *δ* 10.39 (s, 1H, and CONH), 9.89 (s, 1H, and CONH), 9.81 (s, 1H, and CONH), 9.64 (s, 1H, and CONH), 9.60 (s, 1H, and CONH), 8.26-8.24 (m, 1H, and CONH), 8.03-8.01 (m, 1H, and CONH), 7.87 (d, 2H, *J* = 7.4 Hz, and Ar-H of the Fmoc group), 7.66 (d, 2H, *J* = 7.4 Hz, and Ar-H of the Fmoc group), 7.64 (s, 1H, and Im-H), 7.53 (s, 1H, and Im-H), 7.50 (s, 1H, and Im-H), 7.41-3.9 (m, 1H, and CONH), 7.39 (t, 2H, *J* = 7.4 Hz, and Ar-H of the Fmoc group), 7.29 (t, 2H, *J* = 7.4 Hz, and Ar-H of the Fmoc group), 7.22 (d, 1H, *J* = 1.7 Hz, and Py-H), 7.18 (d, 1H, *J* = 1.6 Hz, and Py-H), 7.04 (d, 1H, *J* = 1.6 Hz, and Py-H), 6.94 (s, 1H, and Py-H), 6.92-6.91 (m, 1H, and Py-H), 6.89 (d, 1H, *J* = 1.9 Hz, and Py-H), 6.05 (dd, *J* = 2.6 Hz, *J* = 3.8 Hz, 1H, and Py-H), 4.28 (d, 2H, *J* = 7.0 Hz, and CHCH_2_ of the Fmoc group), 4.20 (t, 1H, *J* =7.0 Hz, and CHCH_2_ of the Fmoc group), 4.01 (s, 3H, and NCH_3_), 3.97 (s, 3H, and NCH_3_), 3.93 (s, 3H, and NCH_3_), 3.88 (s, 3H, and NCH_3_), 3.85 (s, 3H, and NCH_3_), 3.80 (s, 3H, and NCH_3_), 3.30-3.15 (m, 6H, and NHCH_2_CH_2_- × 3), 2.36 (t, 2H, *J* = 6.5 Hz, and COCH_2_CH_2_-), and 1.82-1.78 (m, 2H, and -CH_2_CH_2_CH_2_-); ^13^C-NMR (CDCl_3_): *δ* 171.1, 162.3, 159.7, 159.6, 159.1, 157.2, 155.7, 155.6, 143.7, 141.1, 136.4, 135.5, 135.3, 134.1, 133.5, 133.1, 128.4, 127.6, 126.9, 125.4, 124.9, 123.1, 122.8, 121.8, 121.5, 119.9, 119.5, 119.1, 115.0, 114.4, 113.9, 112.5, 107.3, 104.0, 103.9, 66.8, 47.0, 39.2, 38.92, 38.85, 38.79, 36.8, 36.6, 36.4, 35.5, 33.7, 29.7, and 25.3; HRMS (ESI-TOF) *m*/*z*: calcd for C_54_H_58_N_17_O_9_(M + H)^+^ 1088.4603, found 1088.4604.

### 2.20. (9*H*-Fluoren-9-yl)Methyl (3-(1-Methyl-4-(1-Methyl-4-(1-Methyl-4-(4-(1-Methyl-4-(1-Methyl-4-(1-Methyl-1*H*-Pyrrole-2-Carboxamido)-1*H*-Pyrrole-2-Carboxamido)-1*H*-Pyrrole-2-Carboxamido)Butanamido)-1*H*-Imidazole-2-Carboxamido)-1*H*-Imidazole-2-Carboxamido)-1*H*-Imidazole-2-Carboxamido)Propyl)Carbamate (36)

Py_3_-*γ*-Im_3_-carboxylic acid (338 mg, 0.41 mmol) and **9** (205 mg, 0.62 mmol) were dissolved in dichloromethane (10 mL), and then EDCI (236 mg, 1.23 mmol) and DMAP (90 mg, 0.74 mmol) were added to the solution. After stirring at room temperature for 17 h, the solution was diluted with chloroform (50 mL) and washed with H_2_O (20 mL), 5% NaHCO_3_ aq. (20 mL × 2), and H_2_O (20 mL). The organic layer was dried over anhydrous magnesium sulfate and evaporated to dryness. The residue was subjected to chromatographic separation on a column of silica gel using a 0-5% methanol/chloroform solvent system to give **36** (240 mg, 53% yield) as a slightly brown glass. ^1^H-NMR((DMSO-*d*_6_): *δ* 10.40 (s, 1H, and CONH), 9.88 (s, 1H, and CONH), 9.81 (s, 1H, and CONH), 9.63-9.58 (m, 2H, and CONH × 2), 8.26-8.24 (m, 1H, and CONH), 8.03-8.01 (m, 1H, and CONH), 7.87 (d, 2H, *J* = 7.4 Hz, and Ar-H of the Fmoc group), 7.68 (d, 2H, *J* = 7.4 Hz, and Ar-H of the Fmoc group), 7.64 (s, 1H, and Im-H), 7.54 (s, 1H, and Im-H), 7.51 (s, 1H, and Im-H), 7.40 (t, 2H, *J* = 7.4 Hz, and Ar-H of the Fmoc group), 7.32 (t, 2H, *J* = 7.4 Hz, and Ar-H of the Fmoc group), 7.30-7.27 (1H, m, and CONH), 7.22 (d, 1H, *J* = 1.7 Hz, and Py-H), 7.17 (d, 1H, *J* = 1.7 Hz, and Py-H), 7.04 (d, 1H, *J* = 1.7 Hz, and Py-H), 6.95-6.94 (m, 1H, and Py-H), 6.92-6.90 (m, 1H, and Py-H), 6.89 (d, 1H, *J* = 1.7 Hz, and Py-H), 6.05 (dd, *J* = 2.5 Hz, *J* = 3.9 Hz, 1H, and Py-H), 4.31 (d, 2H, *J* = 6.8 Hz, and CHCH_2_ of the Fmoc group), 4.21(t, 1H, *J* = 6.8 Hz, and CHCH_2_ of the Fmoc group), 4.01 (s, 3H, and NCH_3_), 3.97 (s, 3H, and NCH_3_), 3.95 (s, 3H, and NCH_3_), 3.88 (s, 3H, and NCH_3_), 3.84 (s, 3H, and NCH_3_), 3.80 (s, 3H, and NCH_3_), 3.22-3.20 (m, 4H, and NHCH_2_CH_2_- × 2), 3.03-3.01 (m, 2H, and NHCH_2_CH_2-_), 2.39-2.35 (m, 2H, and COCH_2_CH_2_-), 1.82-1.78 (m, 2H, and -CH_2_CH_2_CH_2_-), and 1.64-1.62 (m, 2H, and -CH_2_CH_2_CH_2_-); ^13^C-NMR (DMSO-*d*_6_): *δ* 170.0, 161.4, 158.7, 158.5, 158.5, 156.2, 155.6, 155.4, 143.9, 140.8, 136.5, 135.2, 134.6, 134.4, 133.3, 132.8, 128.1, 127.6, 127.1, 125.5, 125.1, 123.0, 122.8, 122.2, 122.1, 120.1, 118.4, 117.8, 114.8, 114.6, 114.0, 112.7, 106.7, 104.7, 104.3, 65.3, 46.8, 38.2, 37.9, 36.2, 36.1, 36.01, 35.97, 35.3, 35.1, 35.0, 32.9, 29.6, and 25.5; HRMS (ESI-TOF) *m*/*z*: calcd for C_55_H_60_N_17_O_9_ (M + H)^+^ 1102.4760, found 1102.4775.

### 2.21. (9*H*-Fluoren-9-yl)Methyl (4-(1-Methyl-4-(1-Methyl-4-(1-Methyl-4-(4-(1-Methyl-4-(1-Methyl-4-(1-Methyl-1*H*-Pyrrole-2-Carboxamido)-1*H*-Pyrrole-2-Carboxamido)-1*H*-Pyrrole-2-Carboxamido)Butanamido)-1*H*-Imidazole-2-Carboxamido)-1*H*-Imidazole-2-Carboxamido)-1*H*-Imidazole-2-Carboxamido)Butyl)Carbamate (37)

Py_3_-*γ*-Im_3_-carboxylic acid (41 mg, 0.048 mmol) and **10** (28 mg, 0.080 mmol) were dissolved in dichloromethane (3 mL), and then EDCI (28 mg, 0.14 mmol) and DMAP (12 mg, 0.096 mmol) were added to the solution. After stirring at room temperature for 14 h, the solution was diluted with chloroform (50 mL) and washed with H_2_O (20 mL), 5% NaHCO_3_ aq. (20 mL × 2) and H_2_O (20 mL). The organic layer was dried over anhydrous magnesium sulfate and evaporated to dryness. The residue was subjected to chromatographic separation on a column of silica gel using a 0-5% methanol/chloroform solvent system to give **37** (14 mg, 27% yield) as a slightly brown glass. ^1^H-NMR (DMSO-*d*_6_): *δ* 10.40 (s, 1H, and CONH), 9.88 (s, 1H, and CONH), 9.81 (s, 1H, and CONH), 9.63-9.58 (m, 2H, and CONH × 2), 8.25-8.23 (m, 1H, and CONH), 8.04-8.02 (m, 1H, and CONH), 7.87 (d, 2H, *J* = 7.4 Hz, and Ar-H of the Fmoc group), 7.68 (d, 2H, *J* = 7.4 Hz, and Ar-H of the Fmoc group), 7.65 (s, 1H, and Im-H), 7.54 (s, 1H, and Im-H), 7.52 (s, 1H, and Im-H), 7.40 (t, 2H, *J* = 7.1 Hz, and Ar-H of the Fmoc group), 7.32 (t, 2H, *J* = 7.1 Hz, and Ar-H of the Fmoc group), 7.29-7.27 (1H, m, and CONH), 7.23 (d, 1H, *J* = 1.8 Hz, and Py-H), 7.18 (d, 1H, *J* = 1.8 Hz, and Py-H), 7.04 (d, 1H, *J* = 1.8 Hz, and Py-H), 6.95-6.94 (m, 1H, and Py-H), 6.93-6.91 (m, 1H, and Py-H), 6.90 (d, 1H, *J* = 1.8 Hz, and Py-H), 6.05 (dd, 1H, *J* = 2.6 Hz, *J* = 3.9 Hz, and Py-H), 4.29 (d, 2H, *J* = 6.8 Hz, CHCH_2_ of the Fmoc group), 4.19 (t, 1H, *J* = 6.8 Hz, CHCH_2_ of the Fmoc group), 4.01 (s, 3H, and NCH_3_), 3.97 (s, 3H, and NCH_3_), 3.95 (s, 3H, and NCH_3_), 3.88 (s, 3H, and NCH_3_), 3.85 (s, 3H, and NCH_3_), 3.80 (s, 3H, and NCH_3_), 3.22-3.20 (m, 4H, NHCH_2_CH_2_- × 2), 3.00-2.99 (m, 2H, and NHCH_2_CH_2_-), 2.38-2.35 (m, 2H, and COCH_2_CH_2_-), 1.81-1.78 (m, 2H, and -CH_2_CH_2_CH_2_-), 1.46-1.30 (m, 4H, and -CH_2_CH_2_CH_2_- × 2); ^13^C-NMR (DMSO-*d*_6_): *δ* 170.0, 161.3, 158.6, 158.5, 158.4, 156.1, 155.6, 155.4, 143.9, 140.7, 136.5, 135.2, 134.6, 134.5, 133.3, 132.8, 128.1, 127.6, 127.0, 125.5, 125.1, 123.0, 122.8, 122.2, 122.1, 120.1, 118.4, 117.8, 114.8, 114.6, 114.0, 112.7, 106.7, 104.7, 104.2, 65.2, 46.8, 38.2, 38.1, 36.2, 36.1, 36.0, 35.3, 35.1, 35.0, 32.9, 26.9, 26.6, 25.5, and 24.0; HRMS (ESI-TOF) *m*/*z*: calcd for C_56_H_62_N_17_O_9_ (M + H)^+^ 1116.1926, found 1116.4918.

### 2.22. Synthesis of MGB Polyamide-Oligonucleotide Conjugates ON 1-4

Conjugates **ON 1**-**4** were synthesized by the postsynthetic modification method as previously described for the synthesis of **ON 1** (*n* = 3) [13].

CPG support-bound oligonucleotide **18** (11-mer: 5′-d(CGI^F,NPE^AATTTGGC)-3′ or 5′-d(CGI^F,NPE^ACCCTGGC)-3′) was synthesized using a syringe-based system. CPG support-bound 2′-deoxynucleoside **16** (B = C^Bz^, 1000 Å, purchased from Applied Biosystems Pty Ltd.) (2 *μ*mol) was treated with 3% *w*/*v* Cl_3_CCO_2_H in dichloromethane (1.0 mL × 2) for 1 min, followed by washing with acetonitrile (2.0 mL × 2). A 0.1 M solution of 2′-deoxynucleoside 3′-phosphoramidite **17** (B = G^iBu^) in acetonitrile (0.5 mL) and 0.5 M 1*H*-tetrazole in acetonitrile (0.5 mL) were then delivered to the column. Following 10 min, coupling agents were ejected from the column, and the CPG support was washed with acetonitrile (2.0 mL × 2) to give the phosphite dimer. Following this, 1 : 1 : 8 acetic anhydride/2,6-lutidine/THF (1.0 mL) and 16% 1-methylimidazole/THF (1.0 mL) were delivered to the column, coupling agents were ejected from the column, and the CPG support was washed with acetonitrile (2.0 mL × 2). The resultant CPG support-bound phosphite dimer was treated with 0.02 M I_2_ in 1 : 2 : 7 H_2_O/pyridine/THF (1.0 mL) for 1 min and washed with acetonitrile (2.0 mL × 2) to give the CPG support-bound phosphorotriester dimer. Following chain elongation using 2′-deoxynucleoside 3′-phosphoramidites **17** (B = T, C^Bz^, A^Bz^, G^iBu^, and I^F,NPE^), as described above, the terminal DMTr protecting group of the oligonucleotide was removed by treatment with 3% *w*/*v* Cl_3_CCO_2_H in dichloromethane (1.0 mL × 2) for 1 min, and the CPG support was washed with acetonitrile (2.0 mL × 2). Resultant CPG support-bound oligonucleotide **18** was treated with 0.1 M Fmoc-NH(CH_2_)_n_NH-MGB polyamide (**12**, **13**, **14**, **15**, **29**, **30**, **31**, **35**, **36**, or **37**) in 1 : 5 Et_3_N/1,4-dioxane (1.0 mL) at 60°C for 24 h and then washed with acetonitrile (2.0 mL × 2). Following this, the CPG support-bound oligomer was treated with 0.5 M DBU in pyridine (2.0 mL) at room temperature for 12 h and washed with acetonitrile (2.0 mL × 2). The generated oligomer was then cleaved from the CPG support by treatment with conc. NH_4_OH (1.5 mL × 2) for 2 h at room temperature. The resulting solution was then heated in a sealed vial at 55°C for 6 h. Following evaporation, the residue was dissolved in H_2_O (5.0 mL) and washed with ethyl acetate (5.0 mL × 3), and the aqueous layer was evaporated. The MGB polyamide-oligonucleotide conjugates **ON 1-4** were then purified by reversed-phase HPLC.

**ON 1** (5′-d(CGGAATTTGGC)-3′, G = Py_4_-NH(CH_2_)_n_-G (*n* = 3 − 5)).

**ON 1** (*n* = 3) yields 48.8 A_260_ units from **16** (B = C^Bz^) (2 *μ*mol). HRMS (ESI-TOF) *m*/*z* calcd for C_135_H_168_N_50_O_69_P_10_(M +2H)^2+^ 1951.4276, found 1951.4158. **ON 1** (*n* = 4): yields 34.0 A_260_ units from **16** (B = C^Bz^) (2 *μ*mol). HRMS (ESI-TOF) *m*/*z* calcd for C_136_H_170_N_50_O_69_P_10_(M +2H)^2+^ 1958.4353, found 1958.4075. **ON 1** (*n* = 5): yields 31.4 A_260_ units from **16** (B = C^Bz^) (2 *μ*mol). HRMS (ESI-TOF) *m*/*z* calcd for C_137_H_173_N_50_O_69_P_10_(M +3H)^3+^ 1310.6314, found 1310.5460.

**ON 2** (5′-d(CGGAATTTGGC)-3′, G = Py_3_-NH(CH_2_)_n_-G (*n* = 4).

**ON 2** (*n* = 4) yields 22.5 A_260_ units from **16** (B = C^Bz^) (2 *μ*mol). HRMS (ESI-TOF) *m*/*z* calcd for C_130_H_165_N_48_O_68_P_10_ (M +3H)^3+^ 1265.2768, found 1265.2252.

**ON 3** (5′-d(CGGACCCTGGC)-3′, G = Im_3_-NH(CH_2_)_n_-G (*n* = 3 − 5)).

**ON 3** (n =3) yields 21.8 A_260_ units from **16** (B = C^Bz^) (2 *μ*mol). HRMS (ESI-TOF) *m*/*z* calcd for C_123_H_156_N_51_O_67_P_10_ (M + H)^+^ 3728.7744, found 3728.7866. **ON 3** (*n* = 4) yields 20.7 A_260_ units from **16** (B = C^Bz^) (2 *μ*mol). HRMS (ESI-TOF) *m*/*z* calcd for C_124_H_158_N_51_O_67_P_10_ (M + H)^+^ 3742.7900, found 3742.7827. **ON 3** (*n* = 5) yields 28.7 A_260_ units from **16** (B = C^Bz^) (2 *μ*mol). HRMS (ESI-TOF) *m*/*z* calcd for C_125_H_160_N_51_O_67_P_10_ (M + H)^+^ 3756.8057, found 3756.8152.

**ON 4** (5′-d(CGGACCCTGGC)-3′: G = Py_3_-*γ*-Im_3_-NH(CH_2_)_n_-G (*n* = 2 − 4)).

**ON 4** (*n* = 2) yields 8.4 A_260_ units from **16** (B = C^Bz^) (1 *μ*mol). HRMS (ESI-TOF) *m*/*z* calcd for C_144_H_179_N_58_O_71_P_10_ (M + H)^+^ 4165.9555, found 4165.9580. **ON 4** (*n* = 3) yields 11.0 A_260_ units from **16** (B = C^Bz^) (1 *μ*mol). HRMS (ESI-TOF) *m*/*z* calcd for C_145_H_181_N_58_O_71_P_10_ (M + H)^+^ 4179.9712, found 4179.9858. **ON 4** (*n* = 4) yields 11.3 A_260_ units from **16** (B = C^Bz^) (1 *μ*mol). HRMS (ESI-TOF) *m*/*z* calcd for C_146_H_183_N_58_O_71_P_10_ (M + H)^+^ 4193.9868, found 4194.0024.

### 2.23. Melting Temperature Experiments

Absorbance versus temperature profiles of duplexes in 10 mM sodium phosphate buffer (pH 7.0) containing 10 mM NaCl and 0.1 mM Na_2_EDTA were measured using a TMSPC-8/UV1600 (Shimadzu Co., Ltd.) instrument equipped with a thermoelectrically controlled cell holder at 260 nm and a heating rate of 1.0°C/min. The concentration of each duplex was 4.3 *μ*M [5, 36]. From these melting curves, *T*_m_ values were obtained using a TMSPC-8 system with *T*_m_ analysis software.

### 2.24. Circular Dichroism (CD) Spectropolarimetry

CD spectra of duplexes in 10 mM sodium phosphate buffer (pH 7.0) containing 10 mM NaCl and 0.1 mM Na_2_EDTA were measured using a JASCO J-720 spectropolarimeter equipped with a thermoelectrically controlled cell holder (at 20°C) and a cuvette with a path length of 10 mm. The concentration of each duplex was 5.8 *μ*M [5].

## 3. Results and Discussion

In an effort to examine the effect of linker length or the distance between the guanine base and pyrrole polyamide on the stability of the modified dsDNA (**ON 1**/complementary DNA), we synthesized **ON 1 (***n* = 3, 4, and 5) (5′-d(CG**G**AATTTGGC)-3′: **G** = Py_4_-NH(CH_2_)_n_-G) using 3-aminopropyl [13], 4-aminobutyl, and 5-aminopentyl linkers.

**Scheme 1:**
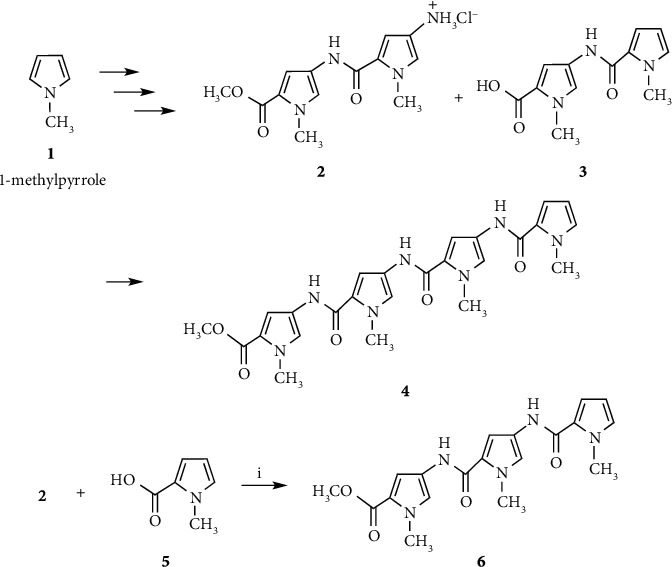
Synthesis of pyrrole polyamides. Reagents and conditions: i EDCI, DMAP, CH_2_Cl_2_, rt, **6** (80%). Compounds **2**, **3**, **4**, and **5** were prepared as previously described [10–13].

**Scheme 2:**
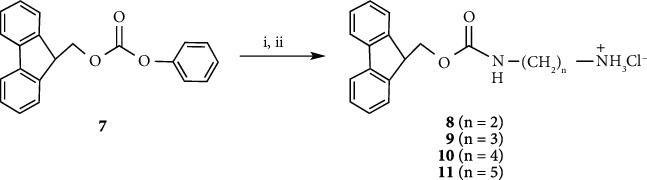
Synthesis of aminoalkyl linker reagents. Reagents and conditions: i H_2_N(CH_2_)_n_NH_2_, MeOH, rt; (ii) pyridinium hydrochloride, **8** (20%); **10** (61%); **11** (45%). Compound **9** was prepared as previously described [10–13].

**Scheme 3:**
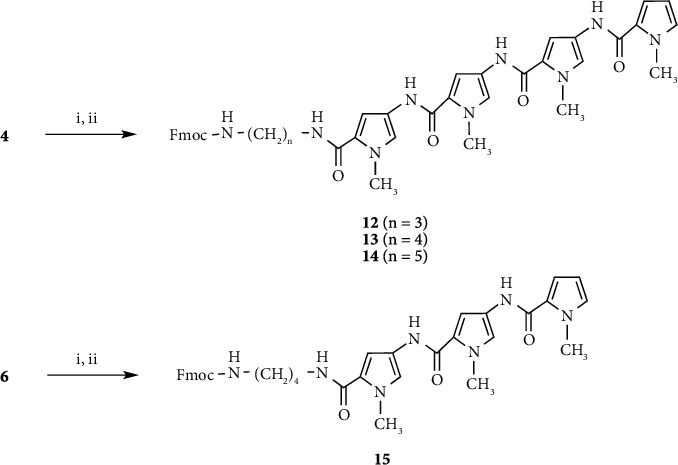
Synthesis of pyrrole polyamides bearing aminoalkyl linker. Reagents and conditions: i 1 M NaOH aq./MeOH, 60°C; Dowex 50WX8 (H^+^-form); ii **9**, **10**, or **11**, DCC, HOBt, DIEA, DMF, and rt; **13** (71%); **14** (84%); **15** (61%). Compound **12** was prepared as previously described [10–13].

**Scheme 4:**
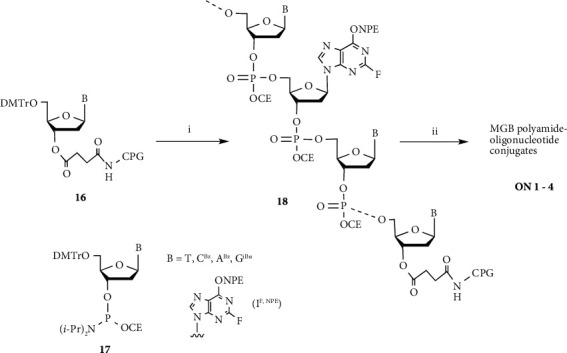
Synthesis of MGB polyamide-oligonucleotide conjugates. Reagents and conditions: i Oligonucleotide assembly on CPG support by the phosphoramidite method, 3% Cl_3_CCO_2_H, CH_2_Cl_2_; 0.05 M phosphoramidite **17**, 0.25 M 1*H*-tetrazole, CH_3_CN, or CH_2_Cl_2_; Ac_2_O, 2,6-lutidine, 1-methylimidazole, THF; and 0.02 M I_2_, H_2_O/pyridine/THF. (ii) 3% Cl_3_CCO_2_H, CH_2_Cl_2_; 0.1 M FmocNH-(CH_2_)_n_NH-MGB polyamide **12**, **13**, **14**, **15**, **29**, **30**, **31**, **35**, **36**, or **37**, 1 : 5 Et_3_N/1,4-dioxane, 60°C, 24 h; 0.5 M DBU, pyridine, rt, 12 h. Conc. NH_4_OH, rt., 2 h–55°C, and 6 h.

Pyrrole amide tetramer **4** and the linker reagent **9** were prepared as previously described (Schemes 1 and 2) [10–13]. Linker reagents **8**, **10,** and **11** were prepared according to the synthetic procedure of **9**. Pyrrole amide tetramers **12**, **13,** and **14** bearing 3-aminopropyl, 4-aminobutyl, and 5-aminopentyl linkers were synthesized via hydrolysis of the ester moiety of **4** and coupling with linker reagents **9**, **10,** and **11**, respectively, as shown in Scheme 3. **ON 1** (*n* = 3, 4, and 5) were synthesized by a postsynthetic modification method using 2′-deoxy-2-fluoroinosine 3′-phosphoramidite **17** (B = I^F,NPE^) [32–35] and pyrrole amide tetramers **12**, **13**, and **14** as previously described (Scheme 4) [13]. 2′-Deoxy-2-fluoroinosine (I^F,NPE^) was incorporated into CPG support-bound oligonucleotide **18** (5′-d(CGI^F,NPE^AATTTGGC)-3′) using a standard procedure [37]. Resultant CPG support-bound oligonucleotide **18** was treated with 0.1 M **12**, **13**, or **14** in 1 : 5 Et_3_N/1,4-dioxane at 60°C for 24 h. The generated CPG support-bound oligonucleotide was then treated with 0.5 M DBU in pyridine to remove the NPE and CE protecting groups, and then treated with concentrated NH_4_OH to cleave the oligomer from the CPG support and remove the Bz and iBu protecting groups. Conjugates **ON 1** (*n* = 3, 4, and 5) were purified by reversed-phase HPLC to yield 48.8, 34.0, and 31.4 A_260_ units, respectively, from **16** (B = C^Bz^) (2 *μ*mol).

**Figure 3:**
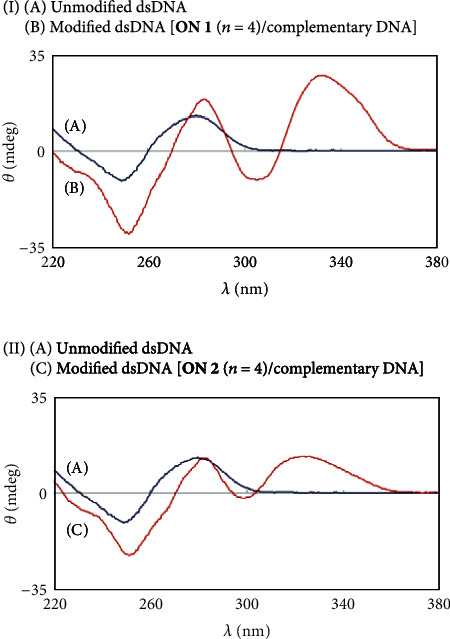
CD spectra of unmodified and modified dsDNAs. modified DNA: 5′-d(CG**G**AATTTGGC)-3′, **ON 1** (**G** = Py_4_-NH(CH_2_)_n_-G) and **ON 2** (**G** = Py_3_-NH(CH_2_)_n_-G).

Conjugates **ON 1** (*n* = 3, 4, and 5) were converted to modified dsDNAs (**ON 1**/complementary DNA) by annealing with complementary DNA. The stability of modified dsDNAs was investigated by *T*_m_ and CD analyses. From the *T*_m_ values, it was found that the stability of modified dsDNA was greatly influenced by the linker length (**ON 1** (*n* = 3, *T*_m_ = 59.5°C, Δ*T*_m_ = +25.4°C), (*n* = 4, *T*_m_ = 60.2°C, Δ*T*_m_ = +26.1°C), and (*n* = 5, *T*_m_ = 52.9°C, Δ*T*_m_ = +18.8°C)) (Table 1). **ON 1** (*n* = 4) showed high binding ability for complementary DNA, similar to **ON 1** (*n* = 3) previously reported [13]. In the CD spectrum for dsDNA [**ON 1** (*n* = 4)/complementary DNA], a strong additional CD band centered at 331 nm resulting from an induced Cotton effect of the bound pyrrole amide moiety was observed (Figure 3(I)) [5, 6, 13].

Using single-mismatch DNA (3′-d(GCCTT**c**AACCG)-5′), which contains a mismatch base in the recognition sequence (5′-d(AATTT)-3′/3′-(TT**A**AA)-5′) of the pyrrole amide moiety, and 2-base mismatch DNA (3′-d(GC**a**TT**c**AACCG)-5′) which does not form dsDNA with the unmodified DNA, the DNA sequence recognition ability of **ON 1** (*n* = 4) was investigated by *T*_m_ analysis (Table 1). **ON 1** (*n* = 4) formed dsDNA with single-base mismatch DNA and displayed stabilization of the dsDNA (*T*_m_ = 45.6°C, Δ*T*_m_ = +23.5°C,) by the pyrrole amide moiety. On the other hand, **ON 1** (*n* = 4) did not form dsDNA with 2-base mismatch DNA and the pyrrole amide moiety did not show any activity.

We surmised that shortening the pyrrole amide chain of the modified DNA would be effective in reducing activity and increasing recognition of the target DNA sequence. **ON 2** (*n* = 4) (5′-d(CG**G**AATTTGGC)-5′: **G** = Py_3_-NH(CH_2_)_n_-G) was synthesized by a postsynthetic modification method using pyrrole amide trimer **15**, which was prepared via coupling of pyrrole amide dimer **2** and pyrrole-2-carboxylic acid **5**, hydrolysis of ester product **6**, and condensation with linker reagent **10** (Schemes 1 and 3). **ON 2** (*n* = 4) was purified by reversed-phase HPLC and yielded 22.5 A_260_ units from **16** (B = C^Bz^) (2 *μ*mol) (Scheme 4).

The stability of the modified dsDNA of **ON 2** (*n* = 4) and complementary DNA was investigated (Table 1 and Figure 3(II)). The stability of the modified dsDNA (**ON 2** (*n* = 4)/complementary DNA: *T*_m_ = 50.8°C, Δ*T*_m_ = +16.7°C) was lower compared with modified dsDNA (**ON 1** (*n* = 4)/complementary DNA: *T*_m_ = 60.2°C, Δ*T*_m_ = +26.1°C). The DNA sequence recognition ability of **ON 2** (*n* = 4) was investigated using single- and 2-base mismatch DNAs (Table 1). **ON 2** (*n* = 4) formed dsDNA with single-base mismatch DNA and displayed stabilization of the dsDNA (*T*_m_ = 39.1°C, Δ*T*_m_ = +17.0°C). On the other hand, **ON 2** (*n* = 4) did not form dsDNA with 2-base mismatch DNA. The DNA sequence recognition ability of pyrrole polyamide-oligonucleotide conjugates was not improved. However, from the result of 2-base mismatch DNA, it was thought that conjugates (modified DNAs **ON 1** (*n* = 4) and **ON 2** (*n* = 4)) did not act on single-base mismatch DNA under conditions where dsDNA (unmodified DNA/single-base mismatch DNA) did not form (e.g., processing temperature > *T*_m_ (unmodified DNA/single-base mismatch DNA)).

Polyamides containing 1-methylpyrrole (Py) and 1-methylimidazole (Im) can be combined in antiparallel side-by-side dimeric complexes with the minor groove of dsDNA [22–24]. An imidazole ring on one ligand complemented by a pyrrole ring on a second ligand (Im/Py combination) recognizes G-C base pairs, while a Py/Im combination targets C-G base pairs. A Py/Py combination is partially degenerate and binds either A-T or T-A base pairs. Based on the results of **ON 1** and **ON 2** described above, it was expected that imidazole polyamide-oligonucleotide conjugates should possess high binding ability for DNA that includes a guanine (G) base. Next, we synthesized and evaluated imidazole polyamide-oligonucleotide conjugates **ON 3** (*n* = 3, 4, and 5) (5′-d(CG**G**ACCCTGGC)-3′: **G** = Im_3_-NH(CH_2_)_n_-G) as model modified oligonucleotides, which form dsDNA with complementary DNA (3′-d(GCCTGGGACCG)-5′) that includes the imidazole polyamide binding sequence (Figure 2).

**Scheme 5:**
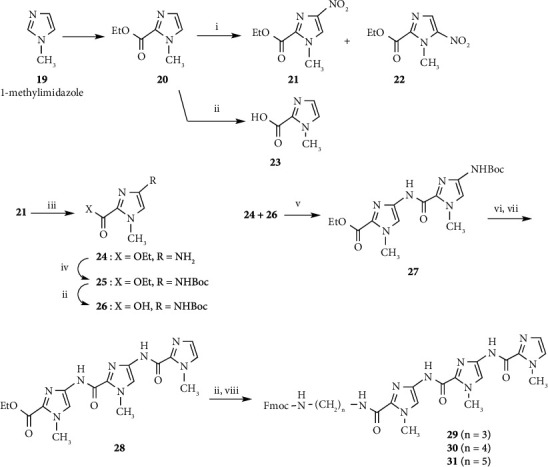
Synthesis of imidazole polyamide derivatives. Reagents and conditions: i (CH_3_)_4_N^+^ NO_3_^−^, TFAA, CHCl_3_, 0°C-rt, **21** (59%), and **22** (24%). ii 1 M NaOH aq./EtOH/pyridine, rt; Dowex 50WX8 (H^+^-form), **23** (quant.); **26** (quant.). iii H_2_, 10% Pd/C, 1 : 1 AcOEt/EtOH, rt, **24** (quant.). iv (Boc)_2_O, DMF, rt, **25** (quant.); (v) DCI, HOBt, DIEA, DMF, rt, **27** (88%). vi AcCl, EtOH/CHCl_3_, rt-40°C. vii **23**, EDCI, DMAP, DMF, rt, **28** (84%). viii **9**, **10,** or **11**, DCI, HOBt, DIEA, DMF, rt, **29** (50%); **30** (33%); **31** (71%). Compound **20** was prepared according to the procedure described by Baird and Dervan [30].

Conjugates **ON 3** (*n* = 3, 4, and 5) were synthesized by a postsynthetic modification method using imidazole amide trimers (**29**, **30** and **31)** as described above (Scheme 4). Imidazole amide trimers (**29**, **30**, and **31**) bearing 3-aminopropyl, 4-aminobutyl, and 5-aminopentyl linkers, respectively, were synthesized as shown in Scheme 5.

Baird and Dervan have reported the nitration of ethyl 1-methylimidazole-2-carboxylate (**20**) by treatment with concentrated sulfuric acid/90% nitric acid [30]. The reaction mixture was refluxed for 50 min and then quenched by pouring on ice. Ethyl 1-methyl-4-nitroimidazole-2-carboxylate (**21**) was extracted with dichloromethane and recrystallized from 21 : 1 CCl_4_/ethanol in 22% yield. We attempted an improvement of the nitration method of **20** using tetramethylammonium nitrate/trifluoroacetic anhydride as a nitrating agent [38]. The reaction was performed at room temperature for 2.5 h. Following the extraction process, the reaction mixture was subjected to silica gel column chromatography using an ethyl acetate/hexane solvent system. Compound **21** and ethyl 1-methyl-5-nitroimidazole-2-carboxylate (**22**) were readily isolated in 59% and 24% yields, respectively.

Compounds **24** and **26** were prepared from **21** according to the procedure described by Baird and Dervan [30]. Imidazole amide trimers (**29**, **30**, and **31**) bearing 3-aminopropyl, 4-aminobutyl, and 5-aminopentyl linkers, respectively, were synthesized via coupling of **24** and **26** to give imidazole amide dimer **27**, deprotection of the Boc group of **27**, coupling with **23** to give imidazole amide trimer **28**, hydrolysis of the ester moiety of **28**, and condensation with 3-aminopropyl, 4-aminobutyl, and 5-aminopentyl linker reagents (**9**, **10** and **11**), respectively. Conjugates **ON 3** (*n* = 3, 4, and 5) were synthesized using imidazole amide trimers (**29**, **30,** and **31**) to yield 21.8, 20.7, and 28.7 A_260_ units, respectively, from **16** (B = C^Bz^) (2 *μ*mol).

**Figure 4:**
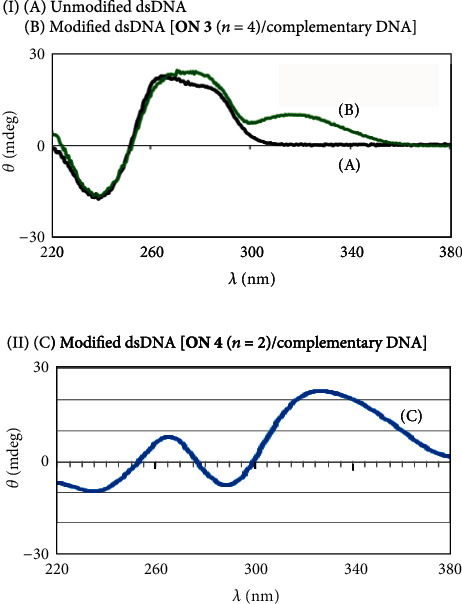
CD spectra of unmodified and modified dsDNAs. modified DNA: 5′-d(CG**G**ACCCTGGC)-3′, **ON 3** (**G** = Im_3_-NH(CH_2_)_n_-G) and **ON 4** (**G** = Py_3_-*γ*-Im_3_-NH(CH_2_)_n_-G).

Conjugates **ON 3** (*n* = 3, 4, and 5) were converted into modified dsDNAs by annealing with complementary DNA.The stability of modified dsDNAs was investigated by *T*_m_ and CD analyses as described above. **ON 3** (*n* = 4) formed more stable dsDNA with complementary DNA (*T*_m_ = 46.5°C, Δ*T*_m_ = +5.1°C) compared with **ON 3** ((*n* = 3, *T*_m_ = 43.7°C, Δ*T*_m_ = +2.3°C) and (*n* = 5, *T*_m_ = 45.6°C, Δ*T*_m_ = +2.2°C)) (Table 2). Moreover, it was determined that the imidazole amide moiety of **ON 3** (*n* = 4) was bound in the minor groove of dsDNA, since an induced CD band of the imidazole amide moiety centered at 314 nm was observed (Figure 4(I)) [5, 6, 13]. Although **ON 3** (*n* = 4) formed stable dsDNA with complementary DNA, stabilization of dsDNA by the imidazole amide moiety of **ON 3** (*n* = 4) was lower compared with the pyrrole amide moiety of **ON 2** (*n* = 4) (Δ*T*_m_ = +16.7°C, Table 1)]. The DNA sequence recognition ability of **ON 3** (*n* = 4) was investigated using two single-base mismatch DNAs (3′-d(GCCTG**a**GACCG)-5′ (mismatch base, **a**: adenine), which have a mismatch base in the sequence recognized by the imidazole amide moiety, and 3′-d(GCCTGGGAC**t**G)-5′ (mismatch base, **t**: thymine)) (Table 2). **ON 3** (*n* = 4) formed modified dsDNA with two single-base mismatch DNAs and showed the same stabilization (*T*_m_ = 35.4°C, Δ*T*_m_ = +1.5°C, and *T*_m_ = 37.1°C, Δ*T*_m_ = +1.6°C) given the low DNA sequence recognition ability of the imidazole amide moiety.

The MGB polyamide hairpin motifs that link the side-by-side MGB polyamides using the *γ*-aminobutyric acid (GABA) linker to favor the heterodimeric binding site have been reported by Dervan et al. [29–31]. A code for the binding of MGB polyamide hairpin motifs has been proposed wherein Py/Im, Im/Py, Hp (3-hydroxy-1-methylpyrrole)/Py and Py/Hp combinations recognize C-G, G-C, T-A, and A-T base pairs, respectively [39–43]. MGB polyamide hairpin motifs can recognize many different sequences of dsDNA and bind in the minor groove of dsDNA according to a set of pairing rules. Novopashina et al. and Boutorine et al. have reported that oligonucleotides conjugated with MGB polyamide hairpin motifs to either the 5′- or 3′-end formed stable dsDNA with target DNA by sequence-specific dsDNA stabilization of MGB polyamide hairpin motifs [16–21].

**Scheme 6:**
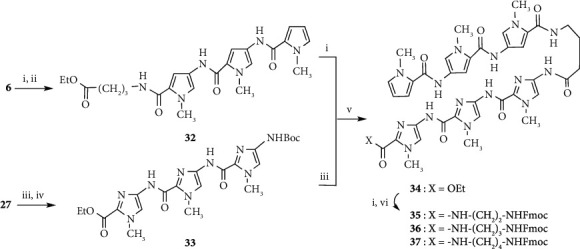
Synthesis of pyrrole-*γ*-imidazole polyamide derivatives. Reagents and conditions: i 1 M NaOH aq./EtOH/pyridine, rt; Dowex 50 WX8 (H^+^-form). ii H_2_N(CH_2_)_3_CO_2_Et, EDCI, DMAP, CH_2_Cl_2_, rt, **32** (82%). iii AcCl, CH_3_OH, rt–40°C. iv **26**, EDCI, DMAP, CH_2_Cl_2_, rt, **33** (70%). v EDCI, DMAP, CH_2_Cl_2_, rt, **34** (57%). vi **8**, **9** or **10**, EDCI, DMAP, CH_2_Cl_2_, rt, **35** (55%); **36** (53%); **37** (27%).

As a further study examining stabilization and recognition abilities, modified DNAs **ON 4** (*n* = 2, 3, and 4) (5′-d(CG**G**ACCCTGGC)-3′: **G** = Py_3_-*γ*-Im_3_-NH(CH_2_)_n_-G) with conjugated pyrrole-imidazole polyamide hairpin motifs, which recognize C-G base pairs via a pyrrole/imidazole combination, at the 2-exocyclic amino group of a guanine base were synthesized and evaluated (Figure 2). Pyrrole-*γ*-imidazole polyamide derivatives (**35**, **36,** and **37**) were synthesized as shown in Scheme 6. Pyrrole trimer **32** was synthesized via hydrolysis of the ester moiety of pyrrole trimer **6** and coupling with ethyl 4-aminobutanoate. Pyrrole trimer **32** was converted into the carboxylic acid compound. Imidazole trimer **33** was synthesized via removal of the Boc group of imidazole dimer **27** and coupling of imidazole monomer **26**. The Boc group of imidazole trimer **33** was removed and then coupled with the carboxylic acid compound to give pyrrole-imidazole amide **34**. Following hydrolysis of the ester moiety of **34**, 2-aminoethyl, 3-aminopropyl, and 4-aminobutyl linker reagents (**8**, **9,** and **10**) were coupled to give pyrrole-*γ*-imidazole amide derivatives (**35**, **36,** and **37**), respectively. Using pyrrole-*γ*-imidazole amide derivatives (**35**, **36,** and **37**), conjugates **ON 4** (*n* = 2, 3, and 4) were synthesized by a postsynthetic modification method to yield 8.4, 11.0, and 11.3 A_260_ units, respectively, from **16** (B = C^B^^z^) (1 *μ*mol) (Scheme 4).

The DNA binding ability of conjugates **ON 4** (*n* = 2, 3, and 4) were investigated by *T*_m_ analysis (Table 2). It was found that modified dsDNAs comprising **ON 4** (*n* = 2, 3, and 4)/complementary DNA possessed higher stability compared with modified dsDNA comprising **ON 3** (*n* = 4)/complementary DNA (*T*_m_ = 46.5°C), and that **ON 4** (*n* = 2) formed the most stable dsDNA with complementary DNA (*T*_m_ = 63.3 °C, Δ*T*_m_ = +21.9°C). Furthermore, we attempted an examination of a modified oligonucleotide using the aminomethyl linker, although the modified oligonucleotide was not synthesized by the same synthetic procedure. From the CD spectra, it was determined that the pyrrole-*γ*-imadazole amide moiety of **ON 4** (*n* = 2) was bound in the minor groove of dsDNA (Figure 4(II)) [5, 6, 13].

The DNA sequence recognition ability of **ON 4** (n =2) was investigated using two single-base mismatch DNAs [3′-d(GCCTG**a**GACCG)-5′ and 3′-d(GCCTGGGAC**t**G)-5′] (Table 2). It was found that **ON 4** (*n* = 2) possessed higher DNA sequence recognition ability, since the mismatch dsDNA [**ON 4** (*n* = 2)/3′-d(GCCTGGGAC**t**G)-5′, *T*_m_ = 55.8°C, Δ*T*_m_ = +20.3°C] possessed higher stability compared with the mismatch dsDNA (**ON 4** (*n* = 2)/3′-d(GCCTG**a**GACCG)-5′, *T*_m_ = 41.1°C. Δ*T*_m_ = +7.2°C].

## 4. Conclusions

We synthesized MGB polyamide-oligonucleotide conjugates with linked MGB polyamides at the 2-exocyclic amino group of a guanine base using various aminoalkyl linkers by a postsynthetic modification method and evaluated the binding affinity for complementary DNA that included the MGB polyamide binding sequence by *T*_m_ and CD analyses. The MGB polyamides comprised pyrrole polyamides (Py_4_- and Py_3_-), which possess binding affinity for A-T base pairs, and imidazole (Im_3_-) and pyrrole-*γ*-imidazole (Py_3_-*γ*-Im_3_-) polyamide hairpin motifs, which possess binding affinity for C-G base pairs. It was found that the stability of the modified dsDNA was greatly influenced by the linker length. Py_4_- and Py_3_-oligonucleotide conjugates (**ON 1** (*n* = 4) and **ON 2** (*n* = 4)] containing the 4-aminobutyl linker formed stable dsDNA with complementary DNA via binding of the MGB polyamide moiety. Although Im_3_-oligonucleotide conjugate **ON 3** (*n* = 4) containing the 4-aminobutyl linker formed stable dsDNA with complementary DNA, stabilization of dsDNA by the imidazole amide moiety of **ON 3** (*n* = 4) was lower compared with the pyrrole amide moiety of **ON 2** (*n* = 4). The Py_3_-*γ*-Im_3_-oligonucleotide conjugates **ON 4** (*n* = 2), which possesses binding affinity for C-G base pairs via a pyrrole/imidazole combination, and contains a 2-aminoethyl linker, showed high binding ability for complementary DNA.

Furthermore, using single-base mismatch DNA, which possess a mismatch base in the pyrrole polyamide binding sequence, and 2-base mismatch DNA, which does not form dsDNA with unmodified DNA, the DNA sequence recognition of conjugates **ON 1** (*n* = 4) and **ON 2** (*n* = 4) was investigated by *T*_m_ analysis. **ON 1** (*n* = 4) formed dsDNA with single-base mismatch DNA and resulted in stabilization of the dsDNA. In the case of 2-base mismatch DNA, **ON 1** (*n* = 4) did not form dsDNA and the pyrrole amide moiety displayed no activity. Examination of **ON 2** (*n* = 4), containing a pyrrole amide moiety with short chain length, showed the same results as **ON 1** (*n* = 4). However, from the result of 2-base mismatch DNA, it was thought that modified DNA conjugates did not act on single-base mismatch DNA under conditions where dsDNA (unmodified DNA/single-base mismatch DNA) does not form. On the other hand, the DNA sequence recognition of conjugates **ON 3** (*n* = 4) and **ON 4** (*n* = 2) was investigated by *T*_m_ analysis using two single-base mismatch DNAs *in lieu* of complementary DNA. Stabilization of the duplex was observed in dsDNAs comprising **ON 3** (*n* = 4) and single-base mismatch DNA, which possess a mismatch base in the imidazole polyamide binding sequence. **ON 4** (*n* = 2) showed high sequence recognition ability for DNA that included the binding sequence of the pyrrole-*γ*-imidazole polyamide hairpin motif. A binding code has been proposed for MGB polyamide hairpin motifs whereby Py/Im, Im/Py, Hp/Py, and Py/Hp combinations recognize C-G, G-C, T-A, and A-T base pairs, respectively [39–43]. MGB polyamide hairpin motif-oligonucleotide conjugates may be utilized to act on dsDNA of various sequences.

It is expected that these results could lead to the development of effective gene expression control compounds and novel anticancer and/or antiviral nucleoside drugs.

## Figures and Tables

**Table 1:** *T*_m_ values of modified dsDNAs and respective Δ*T*_m_ values.

dsDNAs	Complementary DNA		Mismatch DNA ^d)^			
	3′-d(GCCTTAAACCG)-5′		3′-d(GCCTT**c**AACCG)-5′		3′-d(GC**a**TT**c**AACCG)-5′	
	*T*_m_(°C)^b)^	Δ*T*_m_(°C)^c)^	*T*_m_(°C)^b)^	Δ*T*_m_(°C)^c)^	*T*_m_(°C)^b)^	Δ*T*_m_(°C)^c)^
Unmodified DNA	34.1	—	22.1	—	n.d.^e)^	—
Modified DNA ^a)^						
**ON 1** (*n* = 3)	59.5	+25.4^f)^	45.6	+23.5	n.d.^e)^	—
**ON 1** (*n* = 4)	60.2	+26.1				
**ON 1** (*n* = 5)	52.9	+18.8				
**ON 2** (*n* = 4)	50.8	+16.7	39.1	+17.0	n.d.^e)^	—

^a)^modified DNA: 5′-d(CG**G**AATTTGGC)-3′, **ON 1** (**G** = Py_4_-NH(CH_2_)_n_-G); **ON 2** (**G** = Py_3_-NH(CH_2_)_n_-G). ^b)^dsDNA (4.3 *μ*M) in 10 mM sodium phosphate buffer (pH 7.0) containing 10 mM NaCl and 0.1 mM Na_2_EDTA. ^c)^*ΔT*_m_ (°C) = *T*_m_ [modified dsDNA]-*T*_m_ (unmodified dsDNA). ^d)^mismatch base: **a**: adenine; **c**: cytosine. ^e)^ n.d.: not detected. ^f)^it was confirmed that **ON 1** (*n* = 3) formed stable dsDNA with complementary DNA [13].

**Table 2:** *T*_m_ values of modified dsDNAs and respective Δ*T*_m_ values.

dsDNAs	Complementary DNA		Mismatch DNA^d)^			
	3′-d(GCCTGGGACCG)-5′		3′-d(GCCTG**a**GACCG)-5′		3′-d(GCCTGGGAC**t**G)-5′	
	*T*_m_(°C) ^b)^	Δ*T*_m_(°C)^c)^	*T*_m_(°C)^b)^	Δ*T*_m_(°C)^c)^	*T*_m_(°C)^b)^	Δ*T*_m_(°C)^c)^
Unmodified DNA	41.4	—	33.9	—	35.5	—
Modified DNA^a)^						
**ON 3** (*n* = 3)	43.7	+2.3	35.4	+1.5	37.1	+1.6
**ON 3** (*n* = 4)	46.5	+5.1				
**ON 3** (*n* = 5)	43.6	+2.2				
**ON 4** (*n* = 2)	63.3	+21.9	41.1	+7.2	55.8	+20.3
**ON 4** (*n* = 3)	53.6	+12.2				
**ON 4** (*n* = 4)	49.8	+8.4				

a) modified DNA: 5′-d(CG**G**ACCCTGGC)-3′, **ON 3** (**G** = Im_3_-NH(CH_2_)_n_-G); **ON 4** (**G** = Py_3_-*γ*-Im_3_-NH(CH_2_)_n_-G). b) dsDNA (4.3 *μ*M) in 10 mM sodium phosphate buffer (pH 7.0) containing 10 mM NaCl and 0.1 mM Na_2_EDTA. c) *ΔT*_m_ (°C) = *T*_m_ (modified dsDNA)-*T*_m_ (unmodified dsDNA). d) mismatch base, **a**: adenine, **t**: thymine.

## Data Availability

Supporting data for the results of this report are available in the provided supplementary materials.

## Supplementary Materials

Supplementary Materials Figure S1: mass, ^1^H-NMR, and ^13^C-NMR spectra of the synthesized compounds. Figure S2: UV spectra of MGB amide compounds **4**, **6**, **28,** and **34**. Figure S3: HPLC charts of MGB polyamide-oligonucleotide conjugates. Figure S4: mass spectra of MGB polyamide-oligonucleotide conjugates. Figure S5: UV melting curves of modified dsDNAs. Figure S6: CD spectra of modified dsDNAs.
