# Supplementary material for: Synthesis and Evaluation of MGB Polyamide-Oligonucleotide Conjugates as Gene Expression Control Compounds
Source: J Nucleic Acids. 2023 Mar 14;2023:2447998. doi: 10.1155/2023/2447998 (PMC10030224; doi:10.1155/2023/2447998)
Supplement: Supplementary Materials — Figure S1: mass, 1H-NMR, and 13C-NMR spectra of the synthesized compounds. Figure S2: UV spectra of MGB amide compounds 4, 6, 28, and 34. Figure S3: HPLC charts of MGB polyamide-oligonucleotide conjugates. Figure S4: mass spectra of MGB polyamide-oligonucleotide conjugates. Figure S5: UV melting curves of modified dsDNAs. Figure S6: CD spectra of modified dsDNAs. [file 2447998.f1.pdf]

Journal of Nucleic Acids

Supporting Information

Synthesis and evaluation of MGB polyamide-oligonucleotide conjugates as gene expression control compounds

Kazuo Kamaike*, Mutsumi Sano, Daisuke Sakata, Yu Nishihara, Hiroaki Amino, Akihiro Ohtsuki, Yui Okada, Takafumi Miyakawa, Makoto Kogawara, Mai Tsutsumi, Misato Takahashi, Etsuko Kawashima, Koichiro Ota, and Hiroaki Miyaoka

School of Pharmacy, Tokyo University of Pharmacy and Life Sciences, 1432-1 Horinouchi, Hachioji, Tokyo 192-0392, Japan

Correspondence should be addressed to Kazuo Kamaike; kamaikek@toyaku.ac.jp

Contents:

Figure S1: Mass, ^{1}H-NMR and ^{13}C-NMR spectra of the synthesized compounds. S2-S65

Figure S2: UV spectra of MGB amide compounds **4**, **6**, **28** and **34**. S66

Figure S3: HPLC charts of MGB polyamide-oligonucleotide conjugates. S67-S68

Figure S4: Mass spectra of MGB polyamide-oligonucleotide conjugates. S69-S78

Figure S5: UV melting curves of modified dsDNAs. S79-S82

Figure S6: CD spectra of modified dsDNAs. S83

Figure S1: Mass, ^{1}H-NMR and ^{13}C-NMR spectra of the synthesized compounds.

Py$_4$-OMe (**4**)

Elemental Composition Report

Single Mass Analysis
Tolerance = 10.0 mDa / DBE: min = -1.5, max = 300.0
Element prediction: Off
Number of isotope peaks used for i-FIT = 3

Monoisotopic Mass, Even Electron Ions
5 formula(e) evaluated with 1 results within limits (up to 50 closest results for each mass)
Elements Used:
C: 0-300 H: 0-3000 N: 7-7 O: 5-5
mt1-P55
M-18745 297 (2.999) AM2 (Ar,22000.0,0.00,0.00); ABS; Cm (275:305)

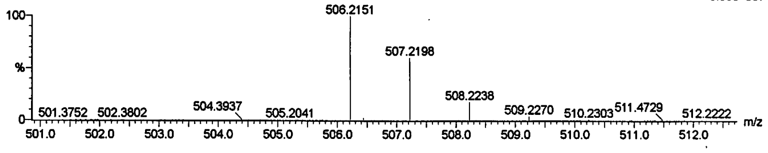

Minimum:				-1.5				
Maximum:		10.0	5.0	300.0				
Mass	Calc. Mass	mDa	PPM	DBE	i-FIT	Norm	Conf(%)	Formula
506.2151	506.2152	-0.1	-0.2	15.5	671.3	n/a	n/a	C25 H28 N7 O5

Elemental Composition Report

Single Mass Analysis
Tolerance = 10.0 mDa / DBE: min = -1.5, max = 300.0
Element prediction: Off
Number of isotope peaks used for i-FIT = 3

Monoisotopic Mass, Even Electron Ions
5 formula(e) evaluated with 1 results within limits (up to 50 closest results for each mass)
Elements Used:
C: 0-300 H: 0-3000 N: 7-7 O: 5-5 Na: 1-1
mt1-P55
M-18745 297 (2.999) AM2 (Ar,22000.0,0.00,0.00); ABS; Cm (275:305)

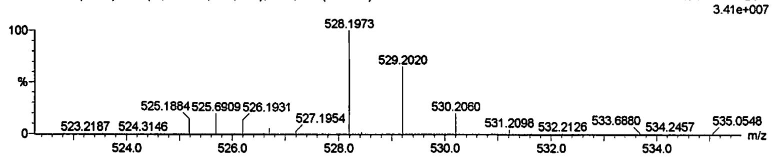

Minimum:				-1.5
Maximum:		10.0	5.0	300.0

[illegible]

Py$_4$-OMe (**4**)

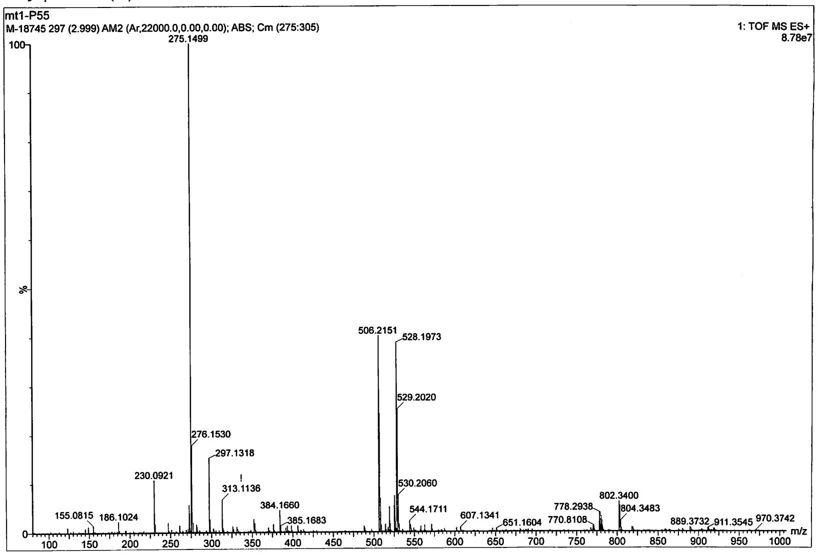

Py$_4$-OMe (**4**) ^{1}H-NMR (DMSO-d$_6$)

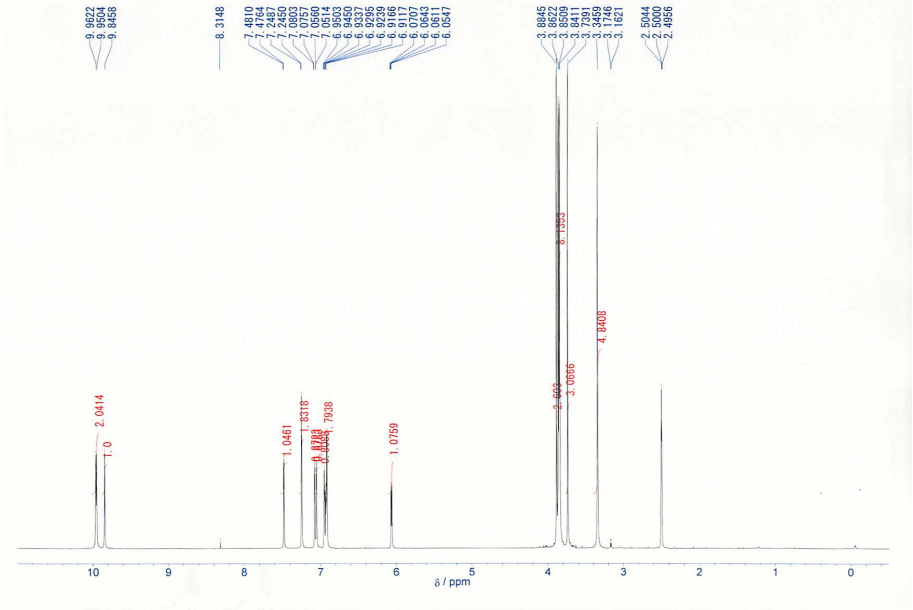

Py$_4$-OMe (**4**) ^{13}C-NMR (DMSO-d$_6$)

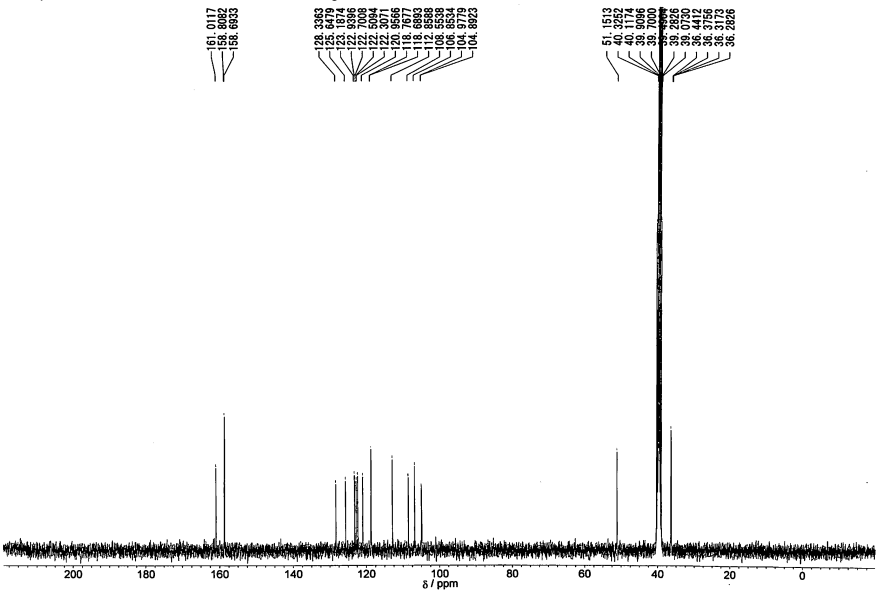

Py$_4$-OH (carboxylic acid)

Elemental Composition Report

Single Mass Analysis

Tolerance = 10.0 mDa / DBE: min = -1.5, max = 300.0

Element prediction: Off

Number of isotope peaks used for i-FIT = 3

Monoisotopic Mass, Even Electron Ions

5 formula(e) evaluated with 1 results within limits (up to 50 closest results for each mass)

Elements Used:

C: 1-300 H: 1-1000 N: 7-7 O: 5-5

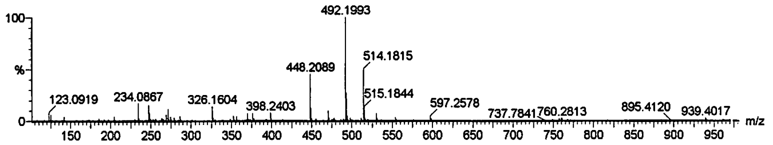

Minimum:				-1.5				
Maximum:		10.0	20.0	300.0				
Mass	Calc. Mass	mDa	PPM	DBE	i-FIT	Norm	Conf(%)	Formula
492.1993	492.1995	-0.2	-0.4	15.5	553.4	n/a	n/a	C24 H26 N7 O5

Py$_4$-OH (carboxylic acid)

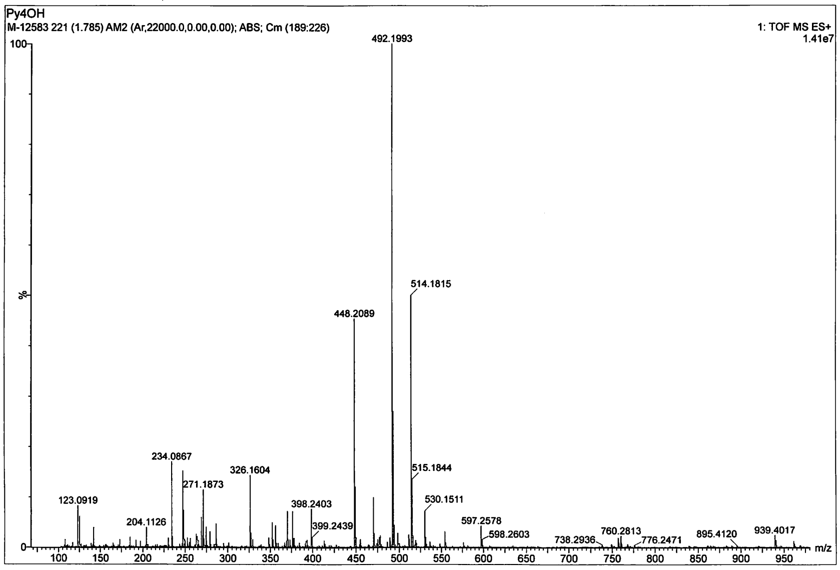

Py$_4$-OH (carboxylic acid) ^{1}H-NMR (DMSO-d_6)

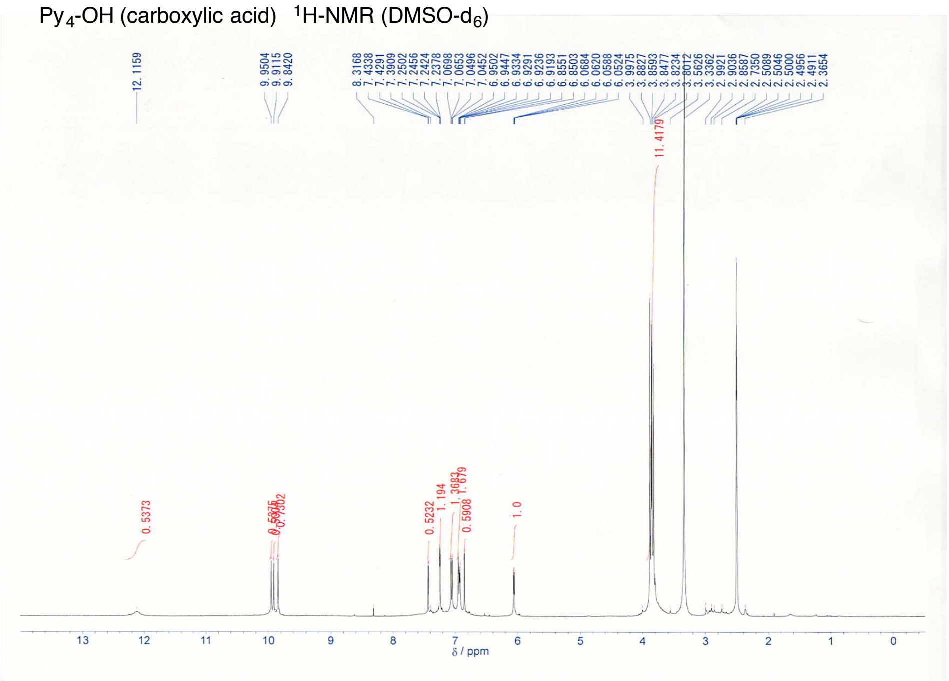

Py$_4$-OH (carboxylic acid) ^{13}C-NMR (DMSO-d_6)

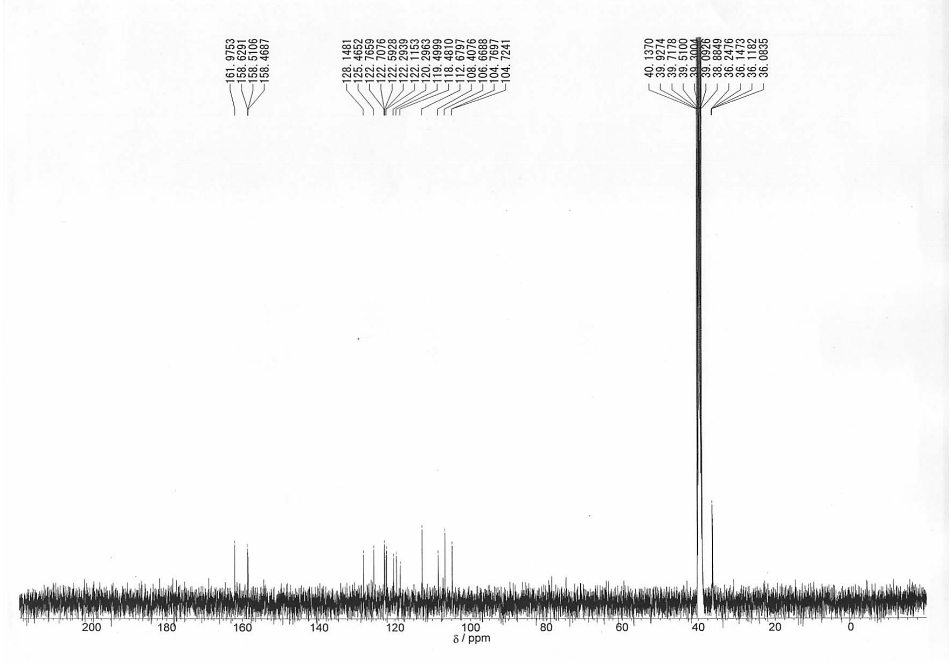

Py$_3$-OMe (**6**)

Elemental Composition Report

Single Mass Analysis (displaying only valid results)
Tolerance = 15.0 PPM / DBE: min = -1.5, max = 40.0
Isotope cluster parameters: Separation = 1.0 Abundance = 1.0%

Monoisotopic Mass, Odd and Even Electron Ions
34 formula(e) evaluated with 1 results within limits (all results (up to 1000) for each mass)

PYPYPY1 22-Sep-2010
SANO 002 16 (0.368) AM (Cen,4, 80.00, Ar,5000.0,608.39,1.00,LS 10); Sm (SG, 5x1.00); Cm (16:35) 1: TOF MS ES+

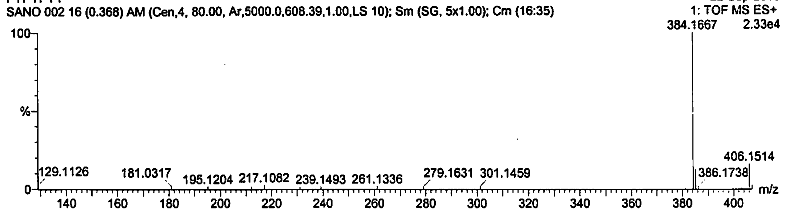

Minimum:				-1.5		
Maximum:		10.0	15.0	40.0		
Mass	Calc. Mass	mDa	PPM	DBE	Score	Formula
384.1667	384.1672	-0.4	-1.1	11.5	1	C19 H22 N5 O4

Py$_3$-OMe (**6**)

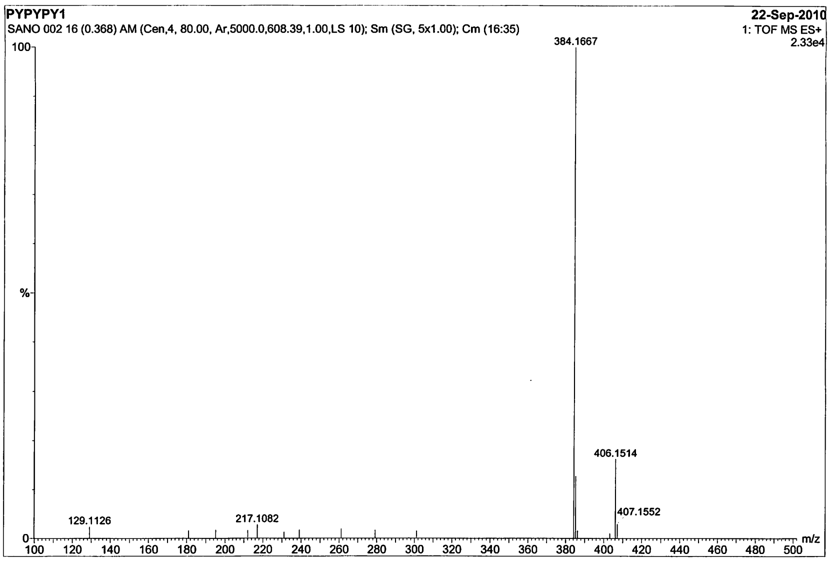

Py$_3$-OMe (**6**) ^{1}H-NMR (DMSO-d_6)

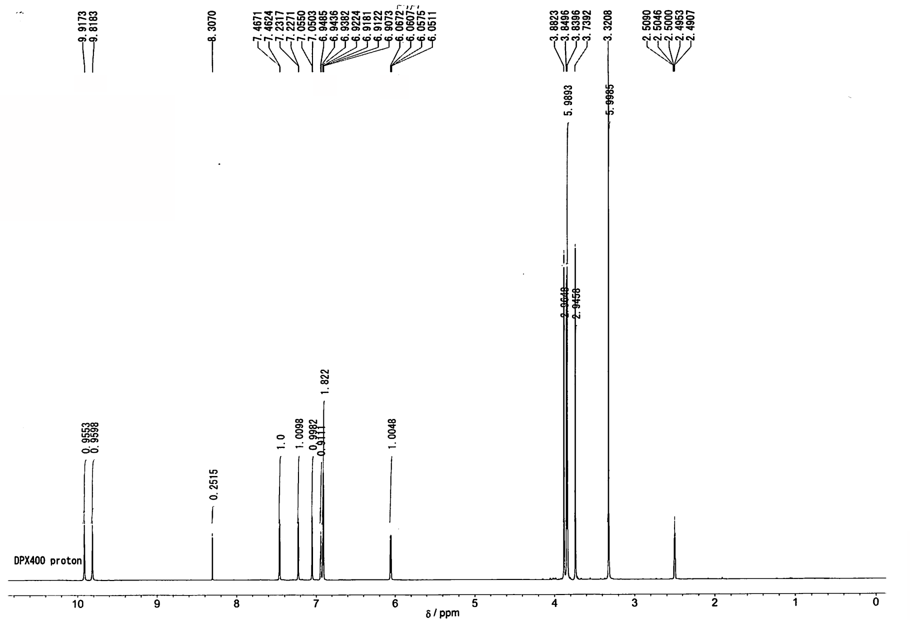

Py$_3$-OMe (**6**) ^{13}C-NMR (DMSO-d_6)

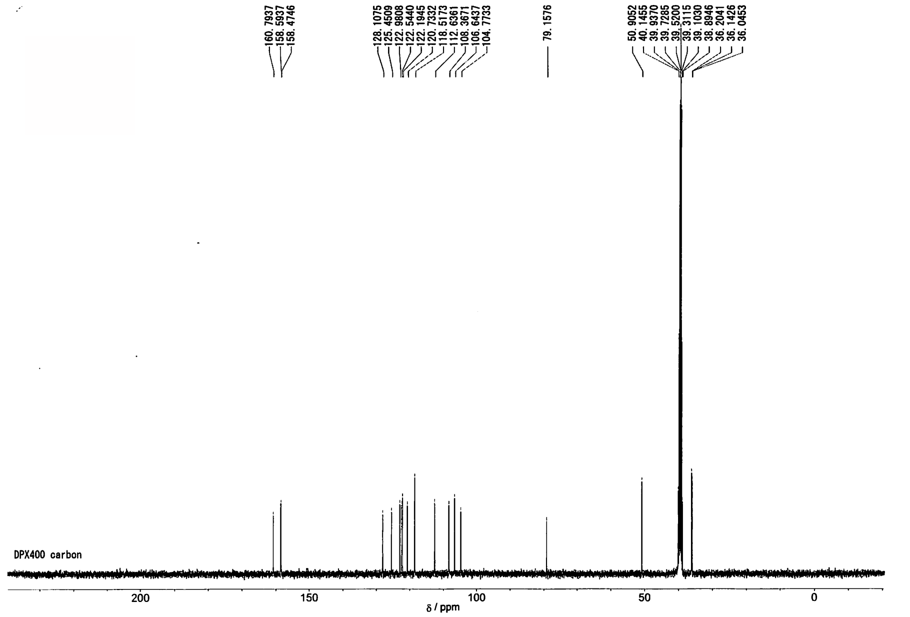

Py$_3$-OH (carboxylic acid)

Elemental Composition Report

Single Mass Analysis
Tolerance = 10.0 mDa / DBE: min = -1.5, max = 300.0
Element prediction: Off
Number of isotope peaks used for i-FIT = 3

Monoisotopic Mass, Even Electron Ions
4 formula(e) evaluated with 1 results within limits (up to 50 closest results for each mass)
Elements Used:
C: 0-300 H: 0-1000 N: 5-5 O: 4-4

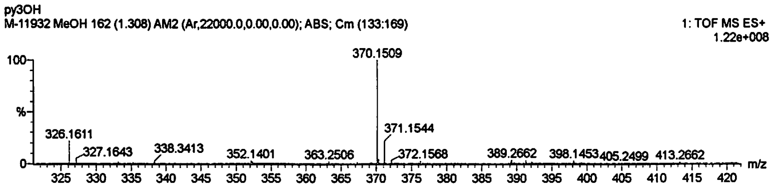

Minimum:				-1.5				
Maximum:		10.0	20.0	300.0				
Mass	Calc. Mass	mDa	PPM	DBE	i-FIT	Norm	Conf(%)	Formula
370.1509	370.1515	-0.6	-1.6	11.5	835.8	n/a	n/a	C18 H20 N5 O4

Py$_3$-OH (carboxylic acid)

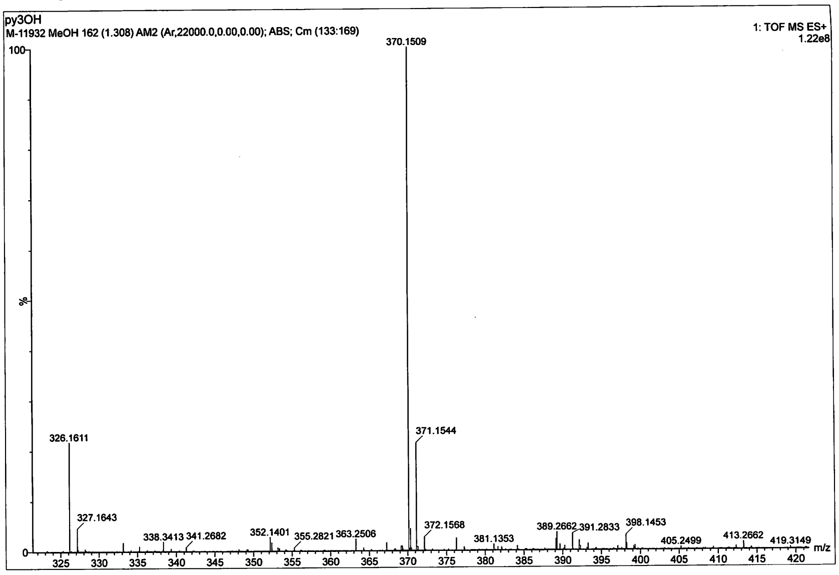

Py$_3$-OH (carboxylic acid) ^{1}H-NMR (DMSO-d$_6$)

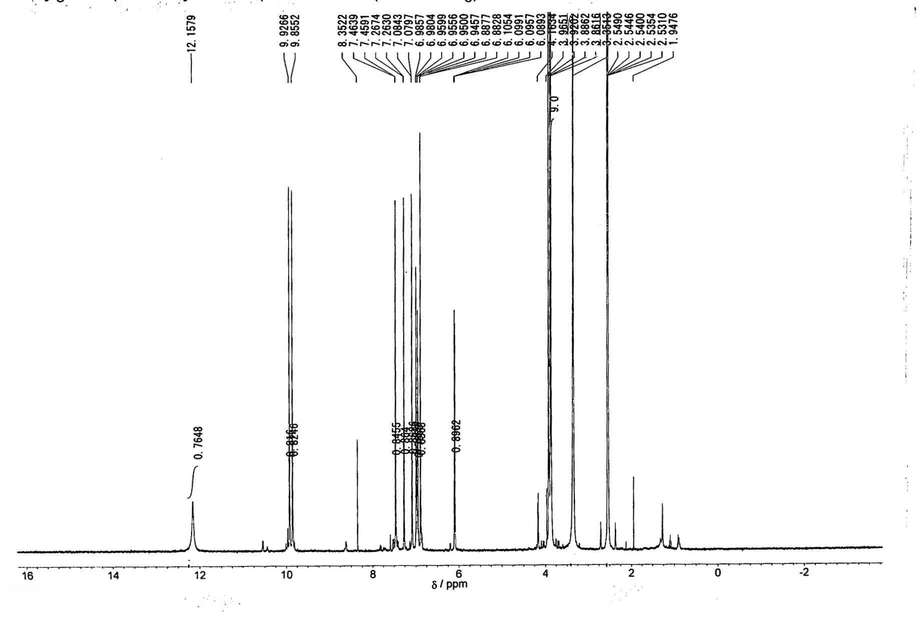

Py$_3$-OH (carboxylic acid) ^{13}C-NMR (DMSO-d$_6$)

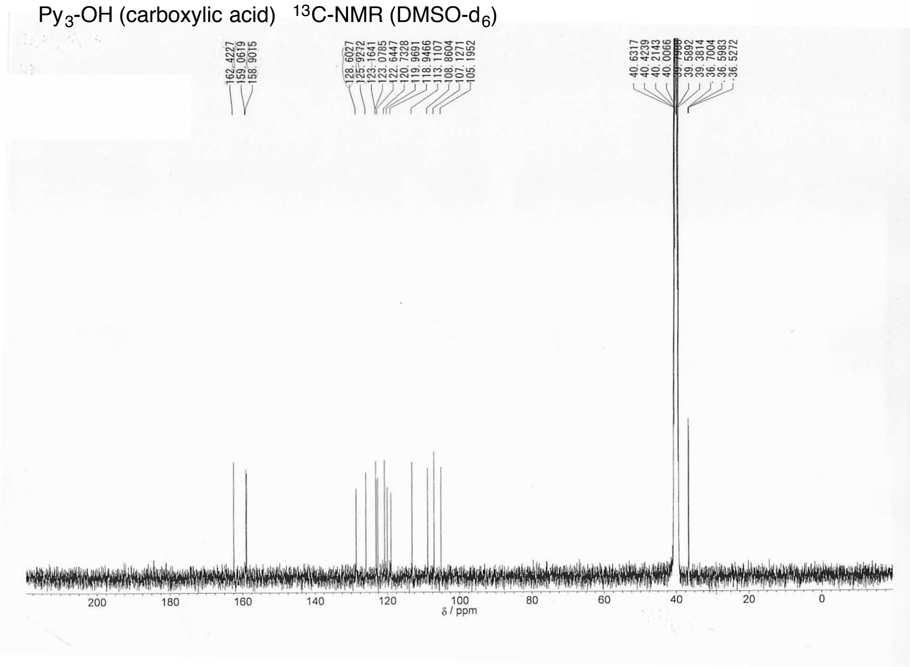

FmocNH(CH$_2$)$_2$NH$_2$ · HCl (**8**)

Elemental Composition Report

Single Mass Analysis
Tolerance = 20.0 PPM / DBE: min = -1.5, max = 50.0
Element prediction: Off
Number of isotope peaks used for i-FIT = 3

Monoisotopic Mass, Even Electron Ions
4 formula(e) evaluated with 1 results within limits (up to 50 closest results for each mass)
Elements Used:
C: 1-300 H: 1-1000 N: 2-2 O: 2-2
Fmoc C 205
M-10247 57 (1.198) AM2 (Ar,30000.0,0.00,0.00); ABS; Cm (56:64)
1: TOF MS ES+
8.71e+006

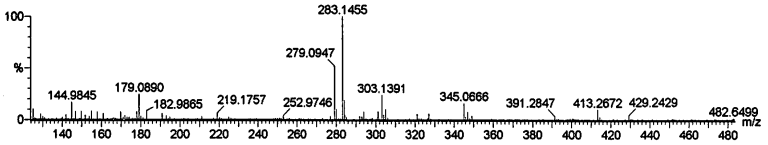

Minimum:				-1.5				
Maximum:		3.0	20.0	50.0				
Mass	Calc. Mass	mDa	PPM	DBE	i-FIT	Norm	Conf(%)	Formula
283.1455	283.1447	0.8	2.8	9.5	897.5	n/a	n/a	C17 H19 N2 O2

FmocNH(CH$_2$)$_2$NH$_2$ · HCl (**8**)

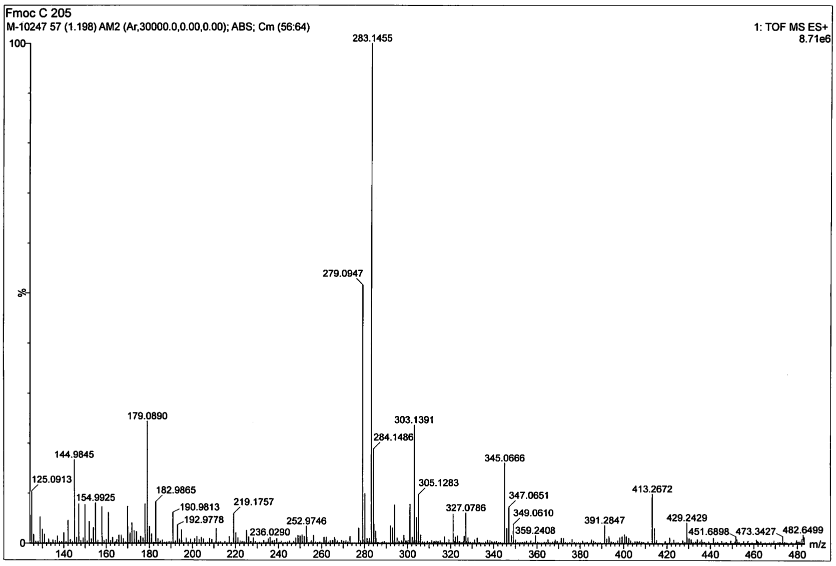

FmocNH(CH$_2$)$_2$NH$_2$ · HCl (**8**) ^{1}H-NMR (CD$_3$OD)

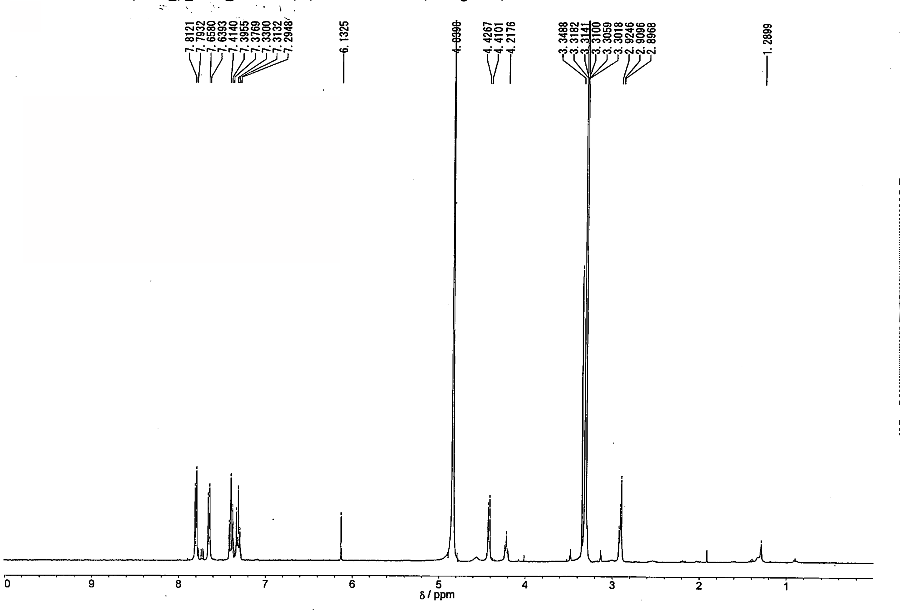

FmocNH(CH$_2$)$_2$NH$_2$ · HCl (**8**) ^{13}C-NMR (CD$_3$OD)

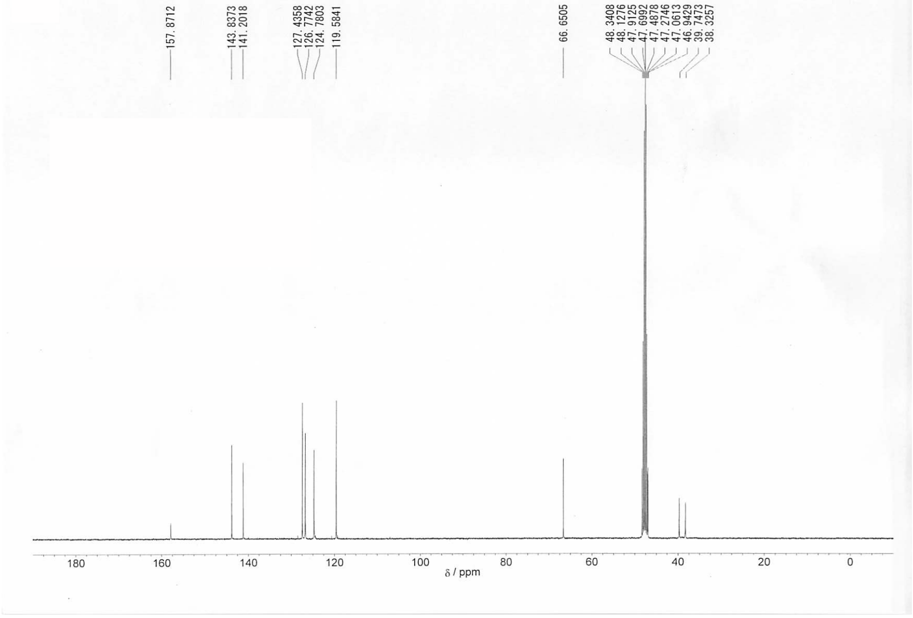

FmocNH$(CH_2)_4NH_2$ · HCl (**10**)

Elemental Composition Report

Single Mass Analysis
Tolerance = 20.0 PPM / DBE: min = -1.5, max = 300.0
Element prediction: Off
Number of isotope peaks used for i-FIT = 3

Monoisotopic Mass, Even Electron Ions
4 formula(e) evaluated with 1 results within limits (up to 50 closest results for each mass)
Elements Used:
C: 1-300 H: 1-1000 N: 2-2 O: 2-2

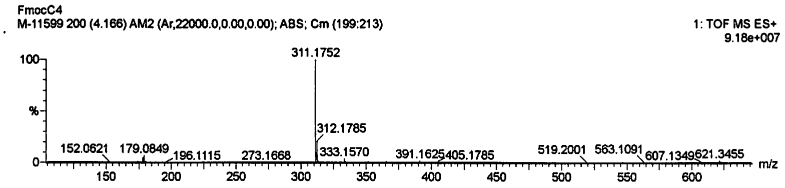

Minimum:				-1.5				
Maximum:		100.0	20.0	300.0				
Mass	Calc. Mass	mDa	PPM	DBE	i-FIT	Norm	Conf(%)	Formula
311.1752	311.1760	-0.8	-2.6	9.5	836.2	n/a	n/a	C19 H23 N2 O2

FmocNH$(CH_2)_4NH_2$ · HCl (**10**)

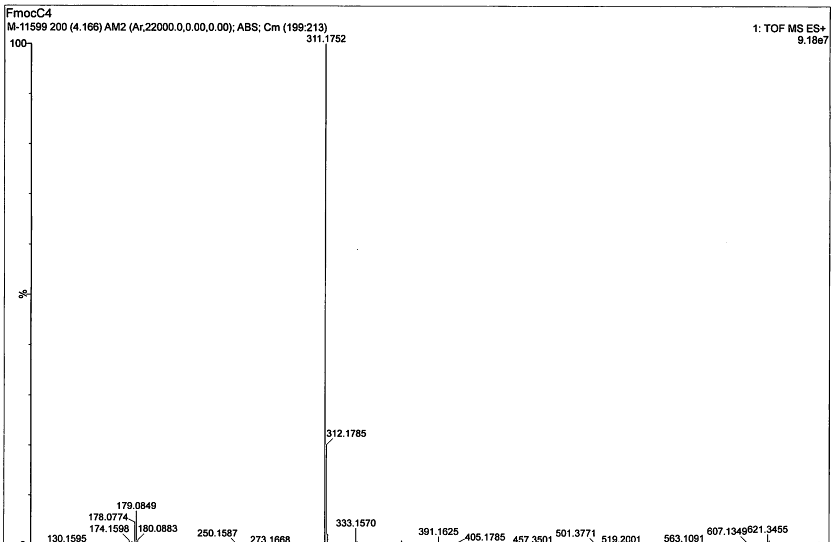

FmocNH(CH$_2$)$_4$NH$_2$ · HCl (**10**) ^{1}H-NMR (CD$_3$OD)

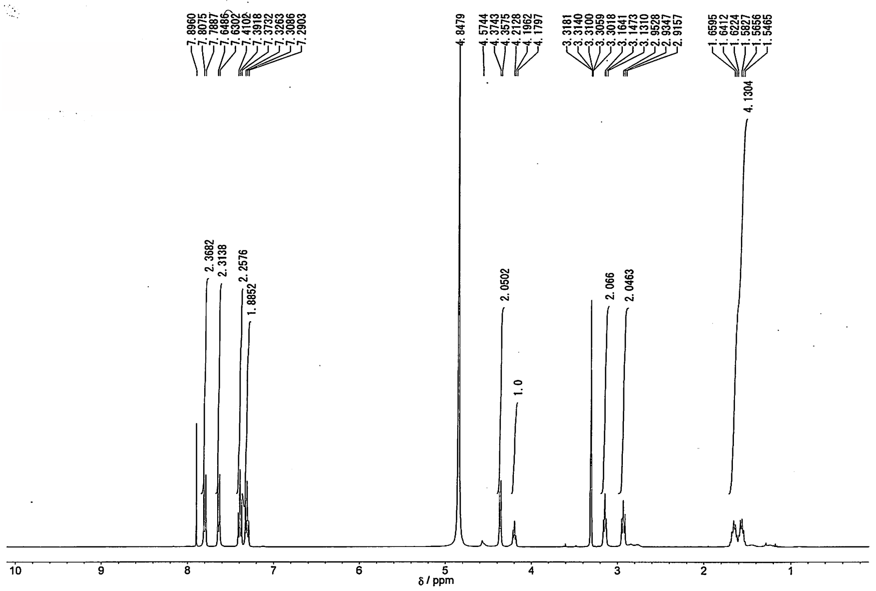

FmocNH(CH$_2$)$_4$NH$_2$ · HCl (**10**) ^{13}C-NMR (DMSO-d$_6$)

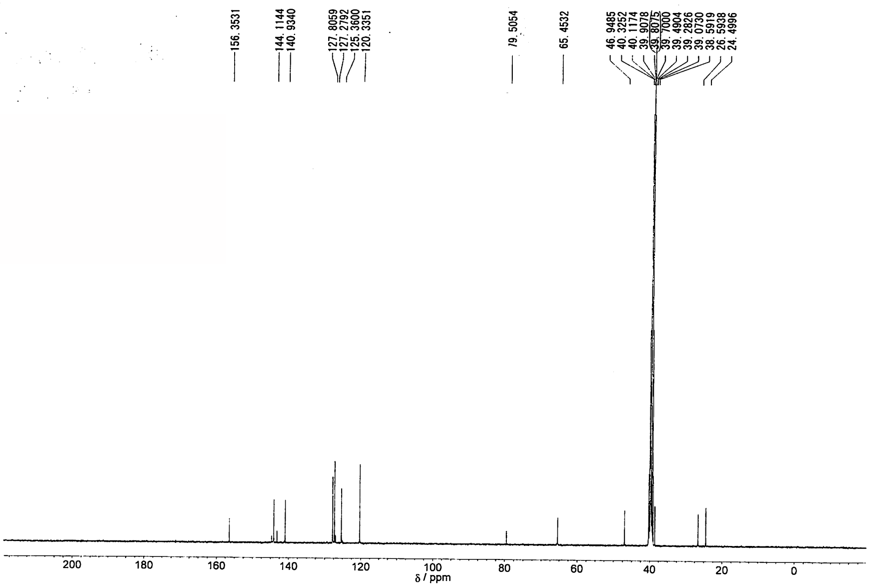

FmocNH(CH_2)$_5$$NH_2$ · HCl (**11**)

Elemental Composition Report

Single Mass Analysis (displaying only valid results)
Tolerance = 10.0 PPM / DBE: min = -1.5, max = 40.0
Isotope cluster parameters: Separation = 1.0 Abundance = 1.0%

Monoisotopic Mass, Odd and Even Electron Ions
113 formula(e) evaluated with 1 results within limits (all results (up to 1000) for each mass)

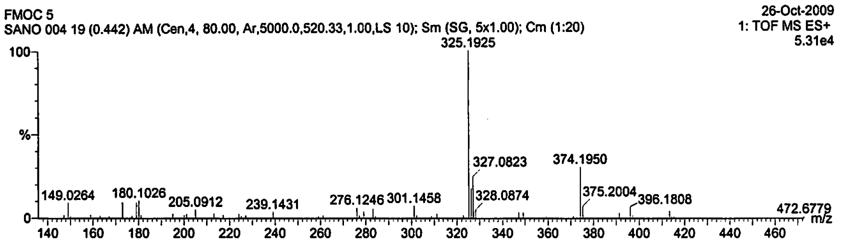

Minimum:				-1.5		
Maximum:		10.0	10.0	40.0		
Mass	Calc. Mass	mDa	PPM	DBE	Score	Formula
325.1925	325.1916	0.9	2.8	9.5	1	C20 H25 N2 O2

FmocNH(CH_2)$_5$$NH_2$ · HCl (**11**)

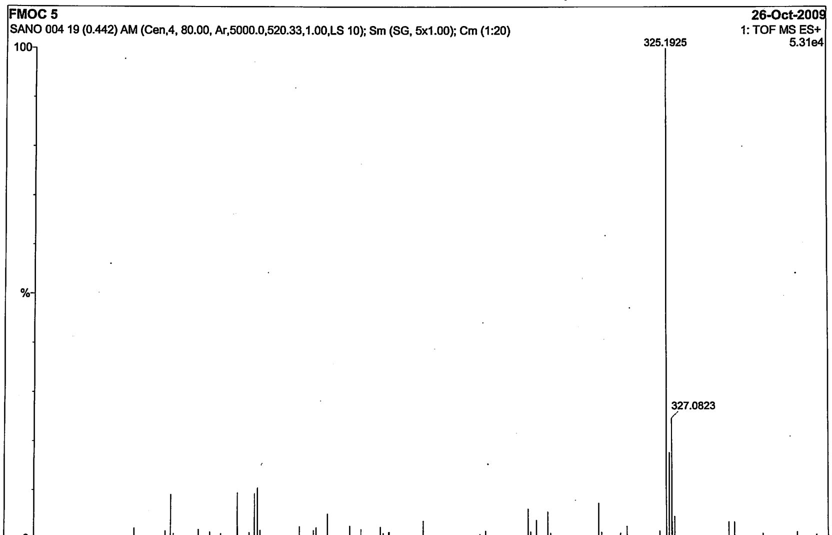

FmocNH(CH$_2$)$_5$NH$_2$ · HCl (**11**) ^{1}H-NMR (CD$_3$OD)

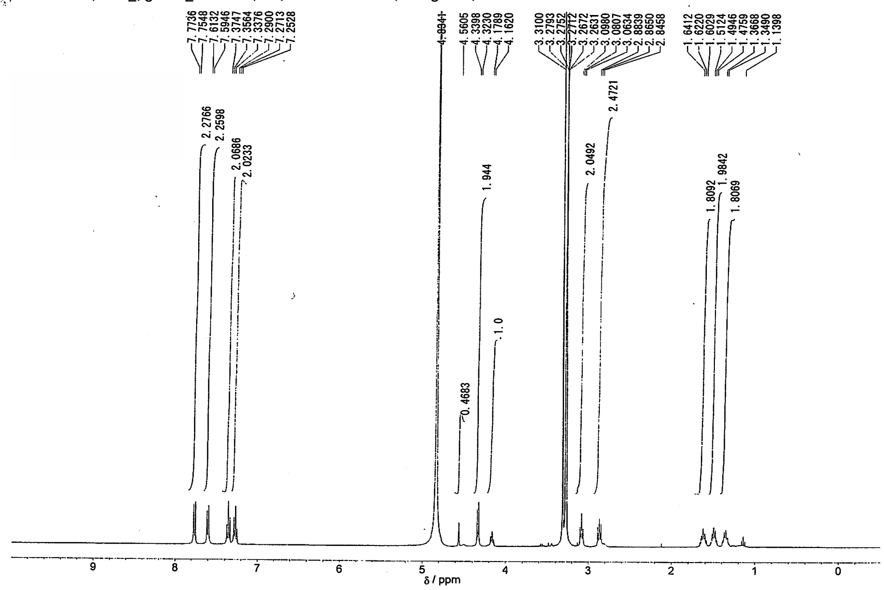

FmocNH(CH$_2$)$_5$NH$_2$ · HCl (**11**) ^{13}C-NMR (CD$_3$OD)

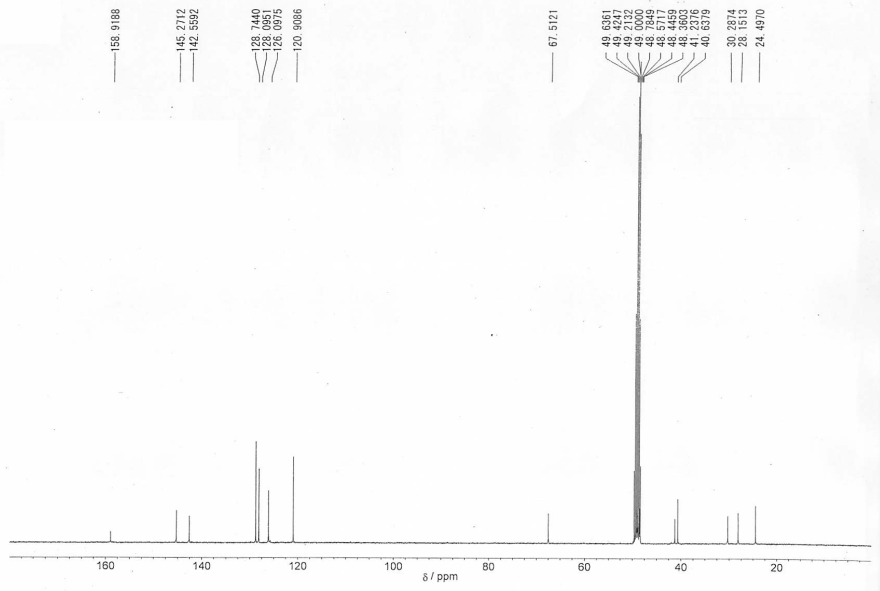

Py$_4$-NH(CH$_2$)$_4$NHFmoc (**13**)

Elemental Composition Report

Single Mass Analysis (displaying only valid results)
Tolerance = 10.0 PPM / DBE: min = -1.5, max = 40.0
Isotope cluster parameters: Separation = 1.0 Abundance = 1.0%

Monoisotopic Mass, Odd and Even Electron Ions
196 formula(e) evaluated with 4 results within limits (all results (up to 1000) for each mass)

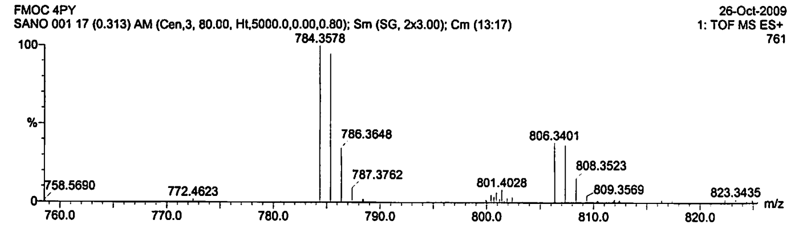

Mass	Calc. Mass	mDa	PPM	DBE	Score	Formula
Minimum:				-1.5		
Maximum:		10.0	10.0	40.0		
784.3578	784.3571	0.7	0.9	25.5	3	C43 H46 N9 O6
	784.3601	-2.2	-2.8	26.0	2	C48 H49 N4 O5 Na
	784.3547	3.1	4.0	22.5	4	C41 H47 N9 O6 Na
	784.3625	-4.6	-5.9	29.0	1	C50 H48 N4 O5

Py$_4$-NH(CH$_2$)$_4$NHFmoc (**13**)

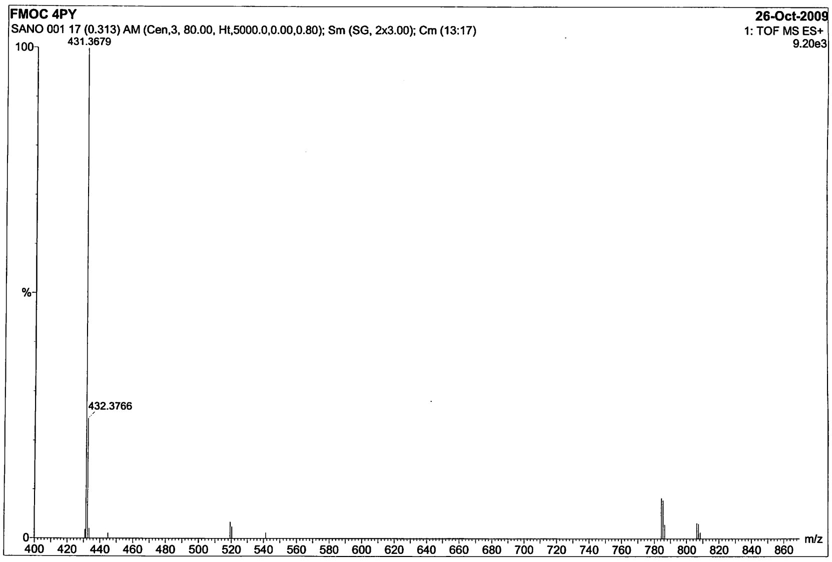

Py$_4$-NH(CH$_2$)$_4$NHFmoc (**13**) ^{1}H-NMR (DMSO-d$_6$)

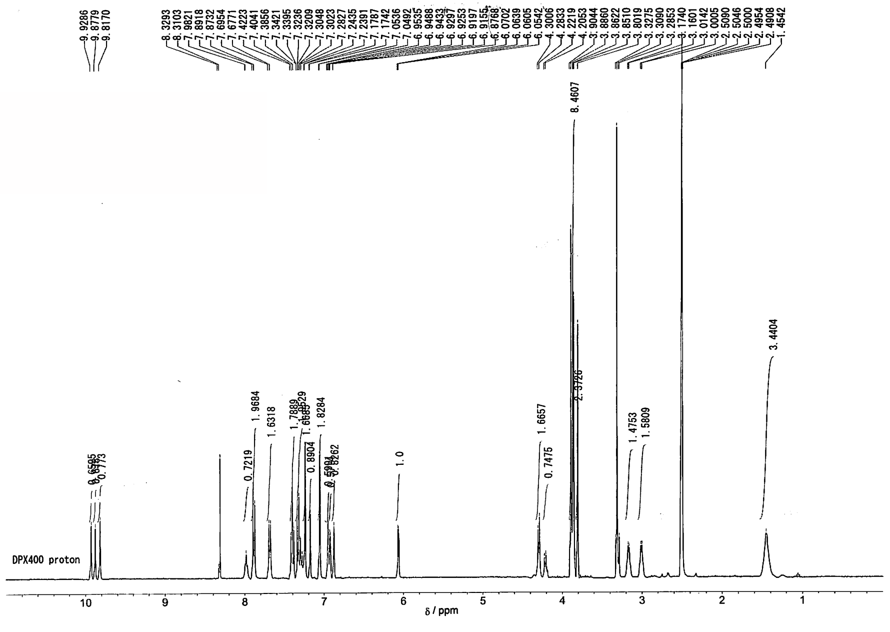

Py$_4$-NH(CH$_2$)$_4$NHFmoc (**13**) ^{13}C-NMR (DMSO-d$_6$)

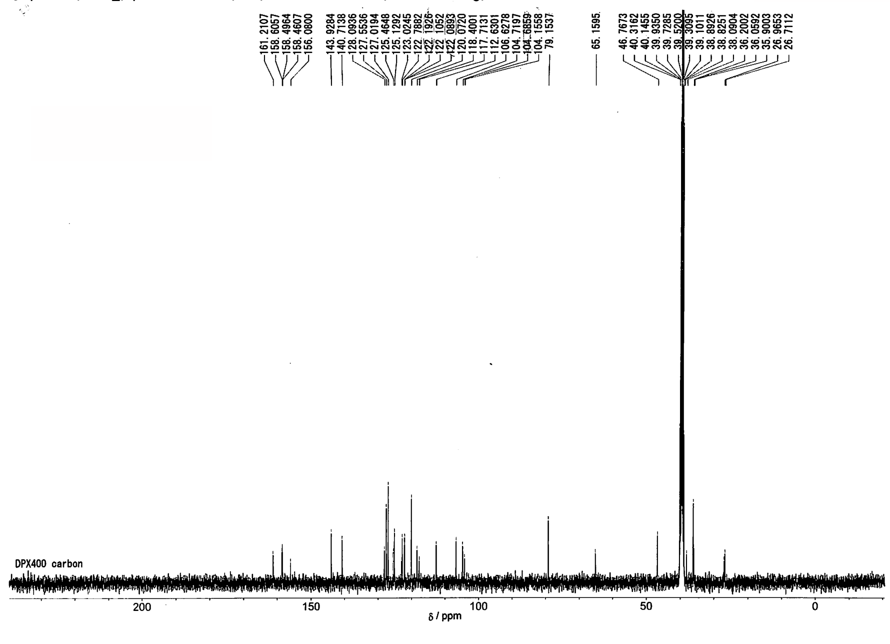

Py$_4$-NH(CH$_2$)$_5$NHFmoc (**14**)

Elemental Composition Report

Single Mass Analysis (displaying only valid results)

Tolerance = 10.0 PPM / DBE: min = -1.5, max = 40.0

Isotope cluster parameters: Separation = 1.0 Abundance = 1.0%

Monoisotopic Mass, Odd and Even Electron Ions

198 formula(e) evaluated with 5 results within limits (all results (up to 1000) for each mass)

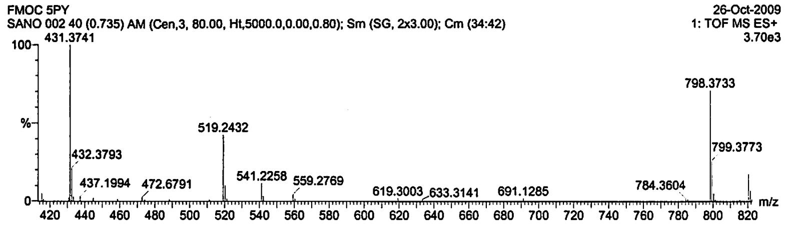

Mass	Calc. Mass	mDa	PPM	DBE	Score	Formula
Minimum:				-1.5		
Maximum:		10.0	10.0	40.0		
798.3733	798.3728	0.5	0.7	25.5	2	C44 H48 N9 O6
	798.3757	-2.4	-3.0	26.0	3	C49 H51 N4 O5 Na
	798.3704	2.9	3.7	22.5	1	C42 H49 N9 O6 Na
	798.3781	-4.8	-6.1	29.0	5	C51 H50 N4 O5
	798.3655	7.7	9.7	29.5	4	C50 H48 N5 O5

Py$_4$-NH(CH$_2$)$_5$NHFmoc (**14**)

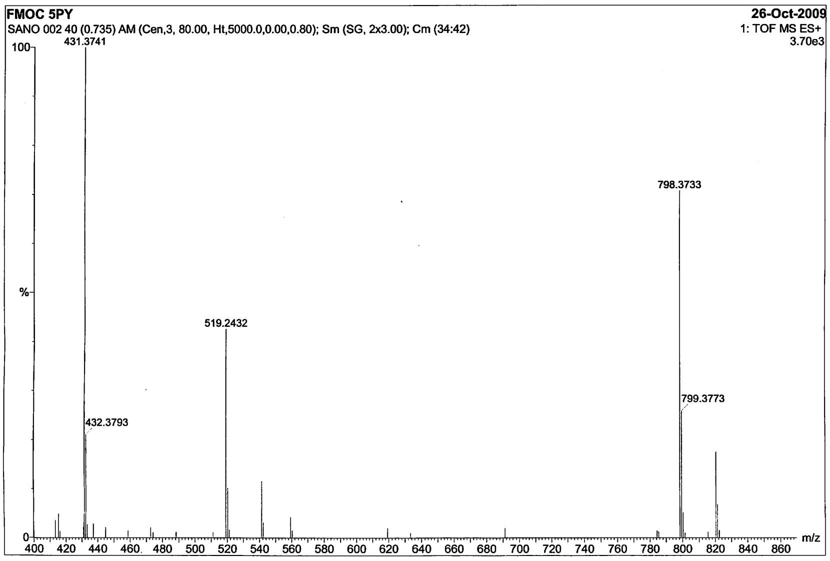

Py$_4$-NH(CH$_2$)$_5$NHFmoc (**14**) ^{1}H-NMR (DMSO-d$_6$)

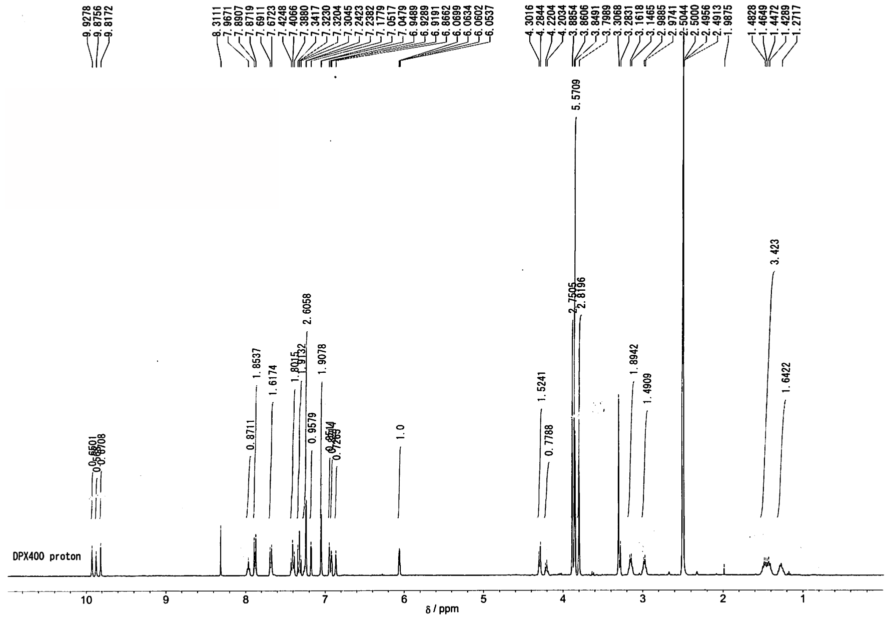

Py$_4$-NH(CH$_2$)$_5$NHFmoc (**14**) ^{13}C-NMR (DMSO-d$_6$)

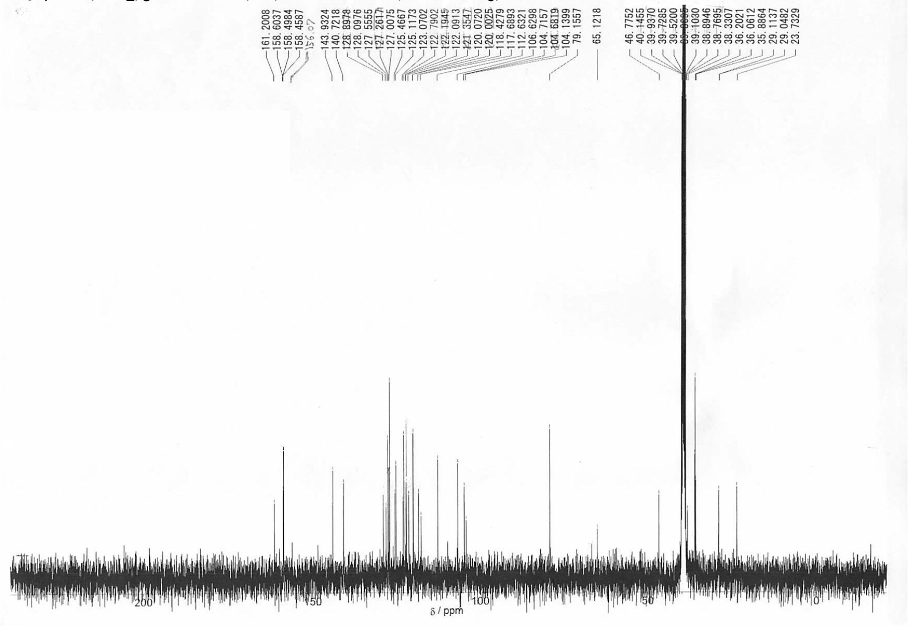

Py$_3$-NH(CH$_2$)$_4$NHFmoc (**15**)

Elemental Composition Report

Single Mass Analysis (displaying only valid results)
Tolerance = 10.0 PPM / DBE: min = -1.5, max = 40.0
Isotope cluster parameters: Separation = 1.0 Abundance = 1.0%

Monoisotopic Mass, Odd and Even Electron Ions
26 formula(e) evaluated with 1 results within limits (all results (up to 1000) for each mass)

F4PPP 26-Nov-2010
SANO 001 71 (1.745) AM (Cen,4, 80.00, Ar,5000.0,520.33,1.00,LS 10); Sm (SG, 5x1.00); Cm (64:87) 1: TOF MS ES+ 2.76e4

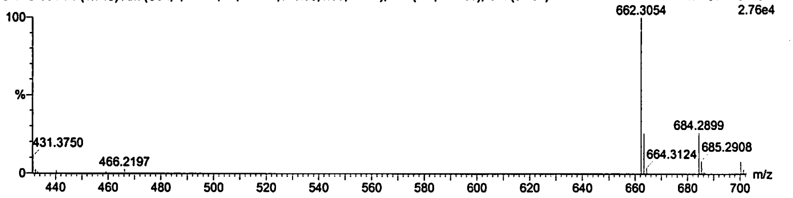

Minimum:				-1.5		
Maximum:		10.0	10.0	40.0		
Mass	Calc. Mass	mDa	PPM	DBE	Score	Formula
662.3054	662.3091	-3.7	-5.5	21.5	1	C37 H40 N7 O5

Py$_3$-NH(CH$_2$)$_4$NHFmoc (**15**)

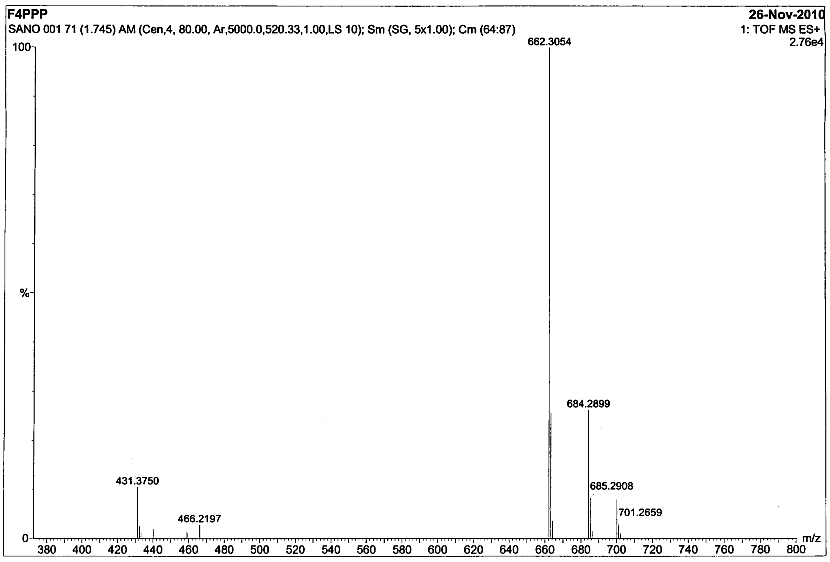

Py$_3$-NH(CH$_2$)$_4$NHFmoc (**15**) ^{1}H-NMR (DMSO-d$_6$)

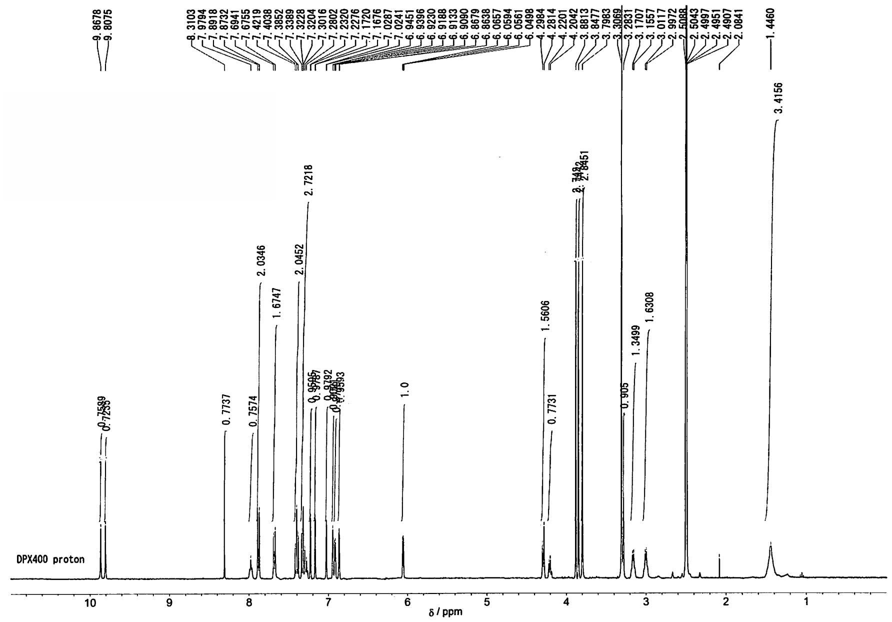

Py$_3$-NH(CH$_2$)$_4$NHFmoc (**15**) ^{13}C-NMR (DMSO-d$_6$)

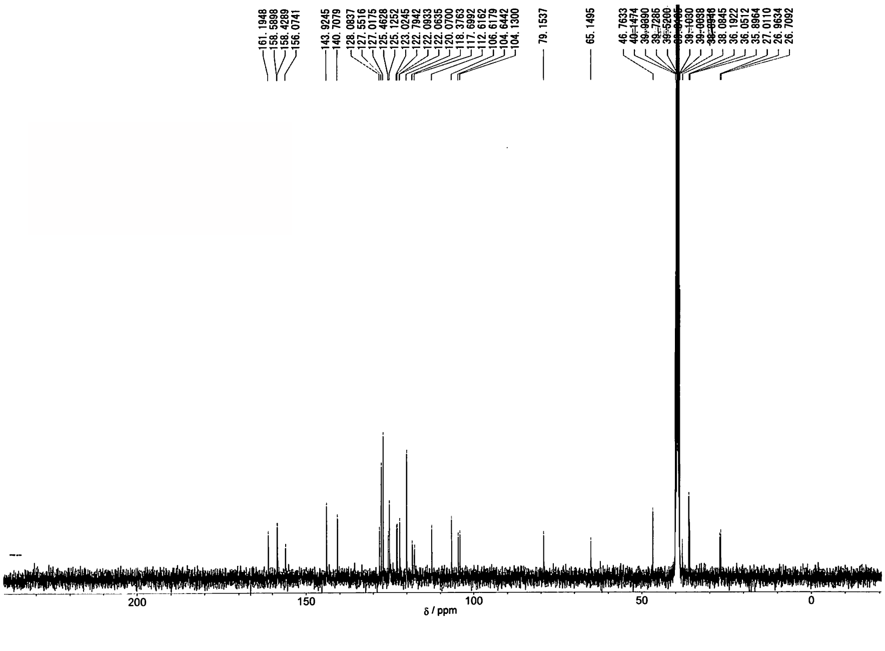

4-O_2NIm-OEt (**21**)

Elemental Composition Report

Single Mass Analysis (displaying only valid results)
Tolerance = 15.0 PPM / DBE: min = -1.5, max = 40.0
Isotope cluster parameters: Separation = 1.0 Abundance = 1.0%

Monoisotopic Mass, Odd and Even Electron Ions
27 formula(e) evaluated with 1 results within limits (all results (up to 1000) for each mass)

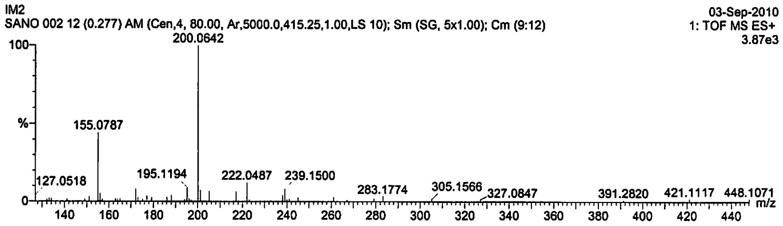

Minimum:				-1.5		
Maximum:		10.0	15.0	40.0		
Mass	Calc. Mass	mDa	PPM	DBE	Score	Formula
200.0642	200.0671	-3.0	-14.8	4.5	1	C7 H10 N3 O4

4-O_2NIm-OEt (**21**)

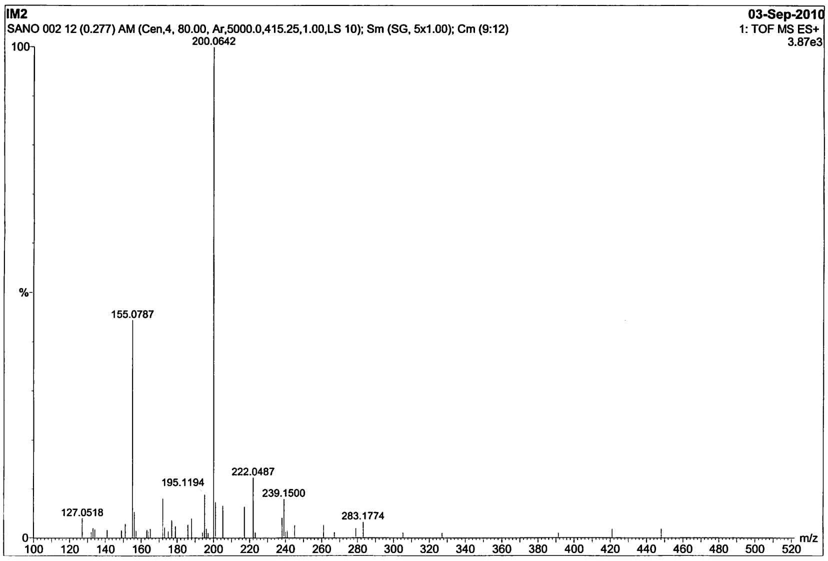

4-O_2NIm-OEt (**21**) ^{1}H-NMR (DMSO-d_6)

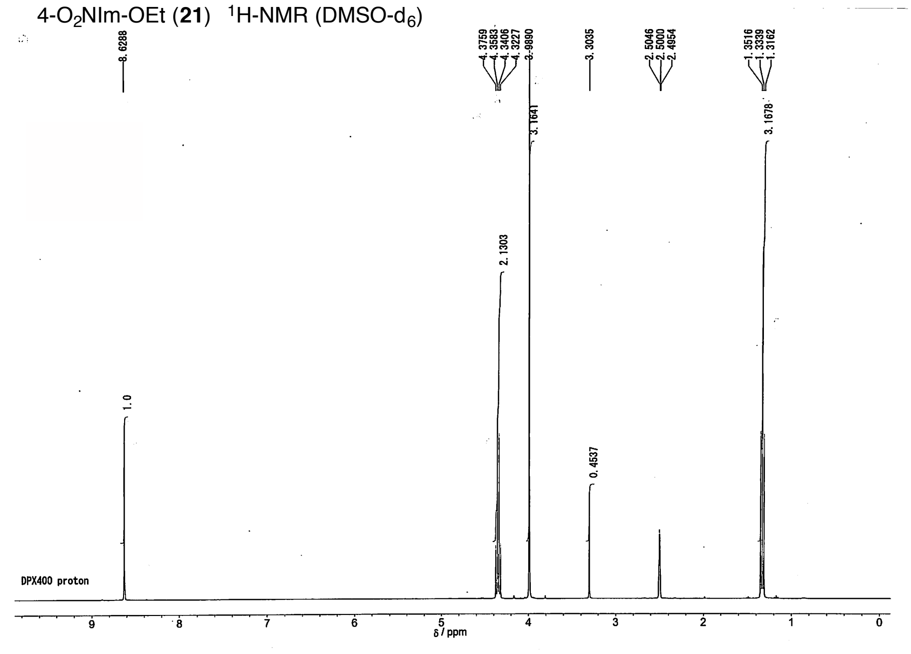

4-O_2NIm-OEt (**21**) ^{13}C-NMR (DMSO-d_6)

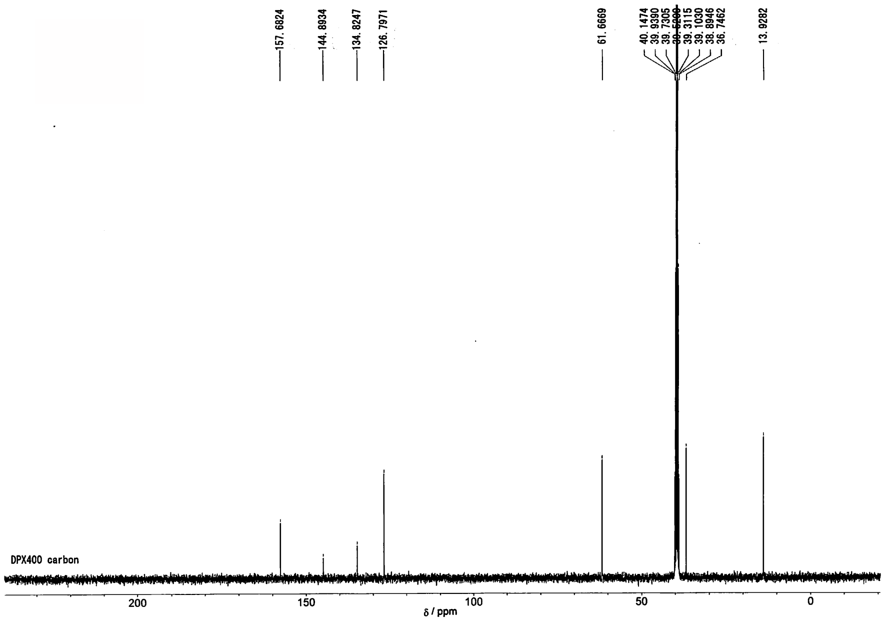

5-O_2NIm-OEt (**22**)

Elemental Composition Report

Single Mass Analysis (displaying only valid results)
Tolerance = 7.0 PPM / DBE: min = -0.5, max = 100.0
Isotope cluster parameters: Separation = 1.0 Abundance = 1.0%

Monoisotopic Mass, Even Electron Ions
213 formula(e) evaluated with 1 results within limits (all results (up to 1000) for each mass)

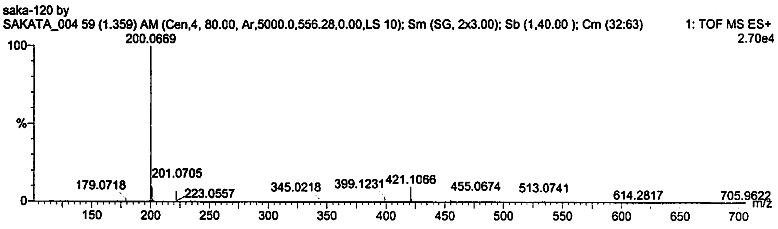

Minimum:				-0.5		
Maximum:		10.0	7.0	100.0		
Mass	Calc. Mass	mDa	PPM	DBE	Score	Formula
200.0669	200.0671	-0.2	-1.2	4.5	1	C7 H10 N3 O4

5-O_2NIm-OEt (**22**)

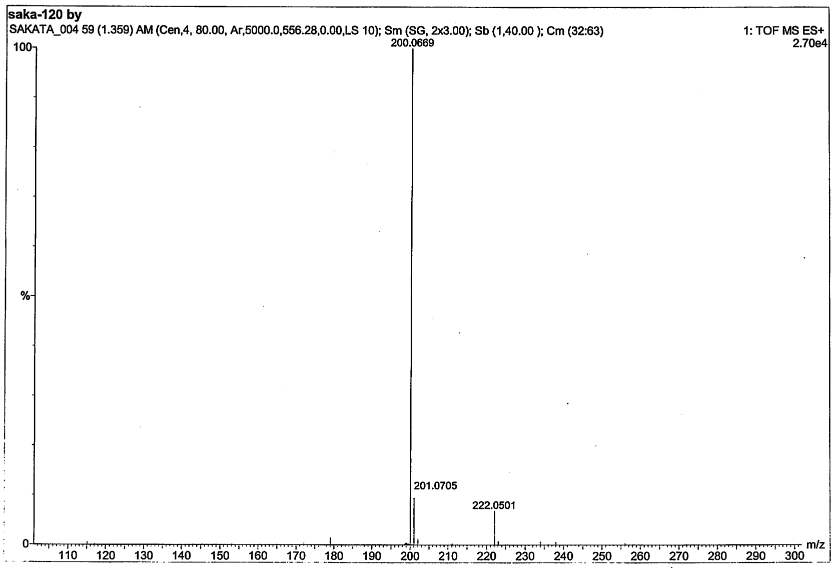

5-O_2NIm-OEt (**22**) ^{1}H-NMR (DMSO-d_6)

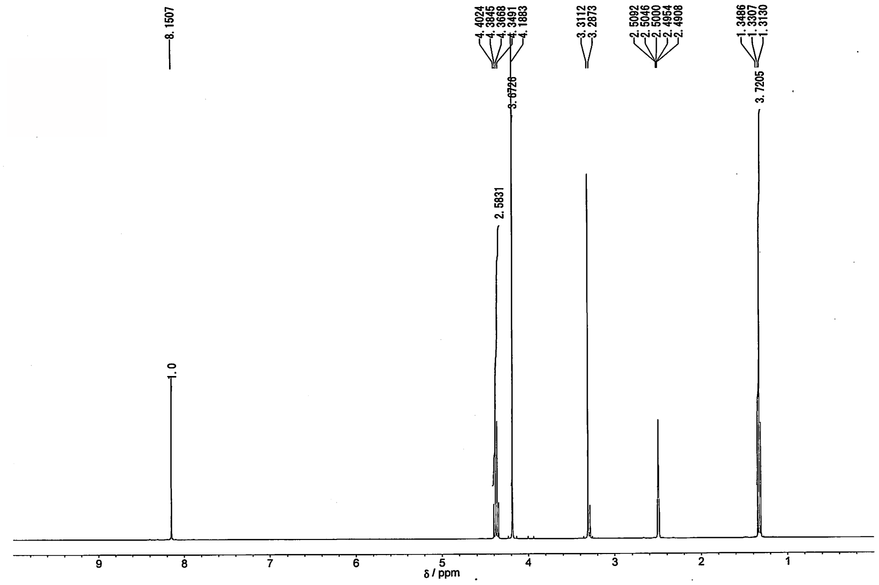

5-O_2NIm-OEt (**22**) ^{13}C-NMR (DMSO-d_6)

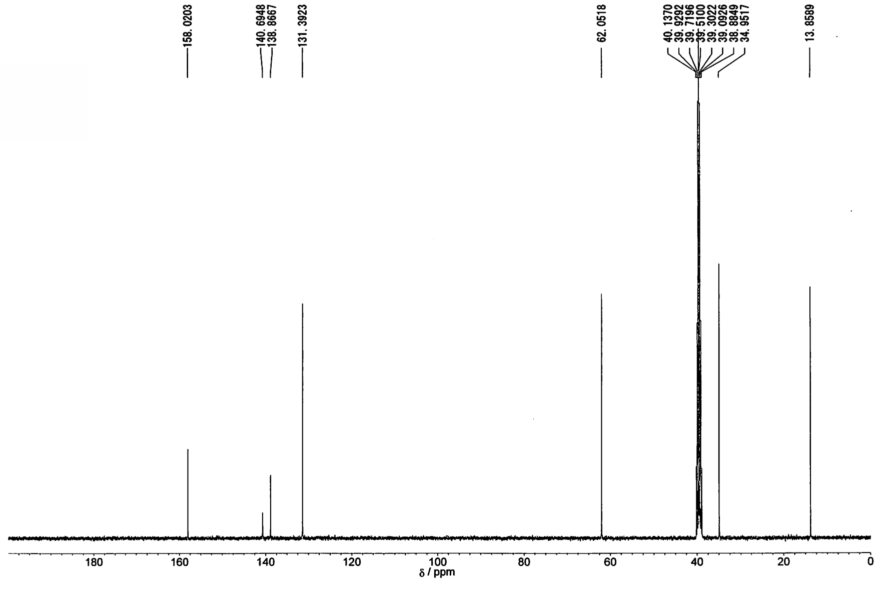

Im-OH (**23**)

Elemental Composition Report

Single Mass Analysis
Tolerance = 20.0 PPM / DBE: min = -1.5, max = 300.0
Element prediction: Off
Number of isotope peaks used for i-FIT = 3

Monoisotopic Mass, Even Electron Ions
2 formula(e) evaluated with 1 results within limits (up to 20 closest results for each mass)
Elements Used:
C: 1-300 H: 1-1000 N: 2-2 O: 2-2
OTUKI-34
M-9682 55 (0.665) Cn (Cen,4, 80.00, Ar); Sm (SG, 2x3.00); Sb (1,40.00); Cm (22:56)　　1: TOF MS ES+ 7.97e+003

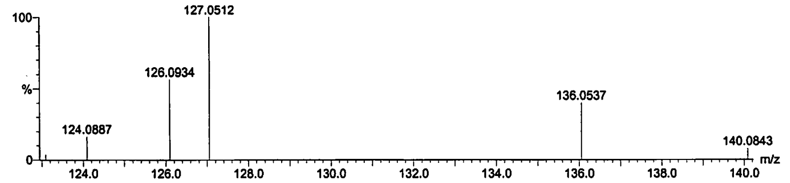

Minimum:				-1.5		
Maximum:		100.0	20.0	300.0		
Mass	Calc. Mass	mDa	PPM	DBE	i-FIT	Formula
127.0512	127.0508	0.4	3.1	3.5	n/a	C5 H7 N2 O2

Im-OH (**23**)

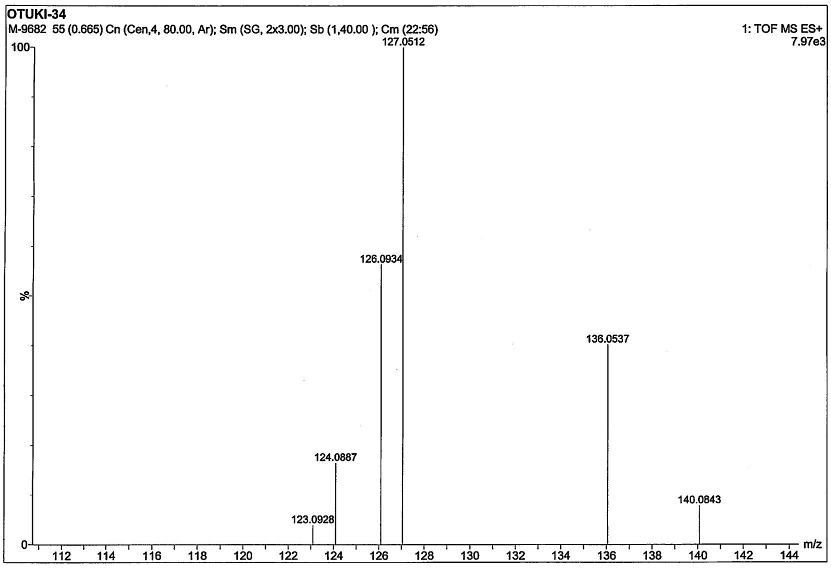

Im-OH (**23**) ^{1}H-NMR (CD_3OD)

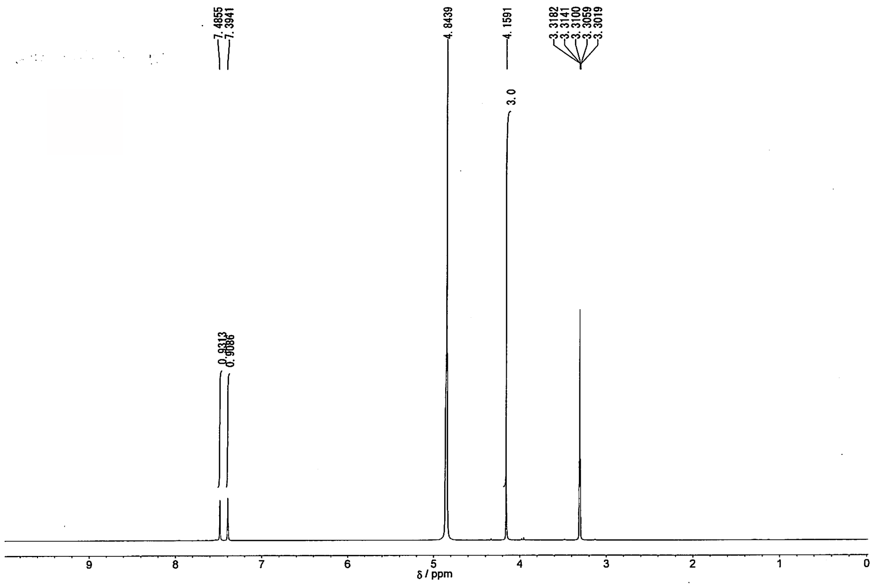

Im-OH (**23**) ^{13}C-NMR (CD_3OD)

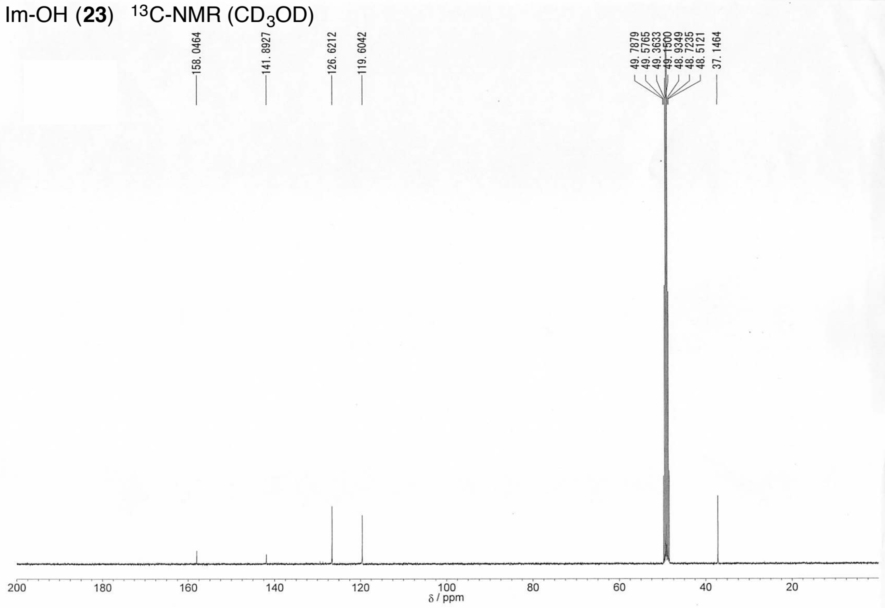

4-H_2NIm-OEt (**24**)

Elemental Composition Report

Single Mass Analysis

Tolerance = 20.0 PPM / DBE: min = -1.5, max = 300.0
Element prediction: Off
Number of isotope peaks used for i-FIT = 3

Monoisotopic Mass, Even Electron Ions
2 formula(e) evaluated with 1 results within limits (up to 20 closest results for each mass)
Elements Used:
C: 1-300 H: 1-1000 N: 3-3 O: 2-2
aki-30
M-10184 15 (0.196) Cn (Cen,4, 80.00, Ar); Sm (SG, 2x3.00); Sb (1,40.00); Cm (15:41)   1: TOF MS ES+ 1.25e+004

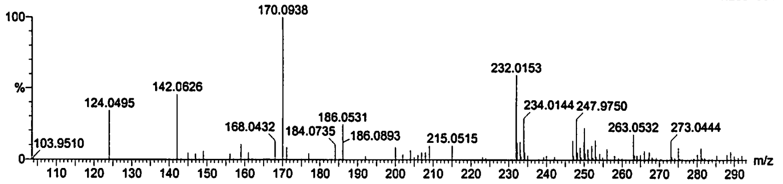

Minimum:				-1.5		
Maximum:		100.0	20.0	300.0		
Mass	Calc. Mass	mDa	PPM	DBE	i-FIT	Formula
170.0938	170.0930	0.8	4.7	3.5	n/a	C7 H12 N3 O2

4-H_2NIm-OEt (**24**)

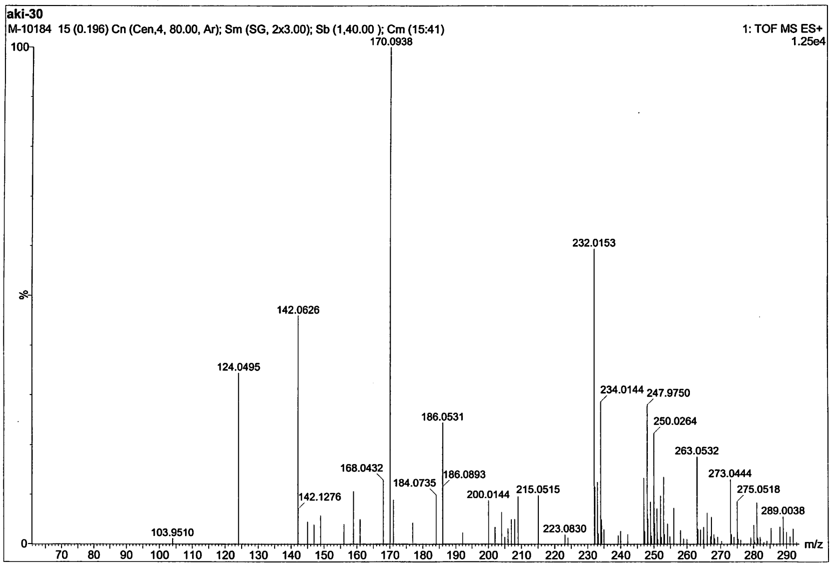

4-H_2NIm-OEt (**24**) ^{1}H-NMR ($CDCl_3$)

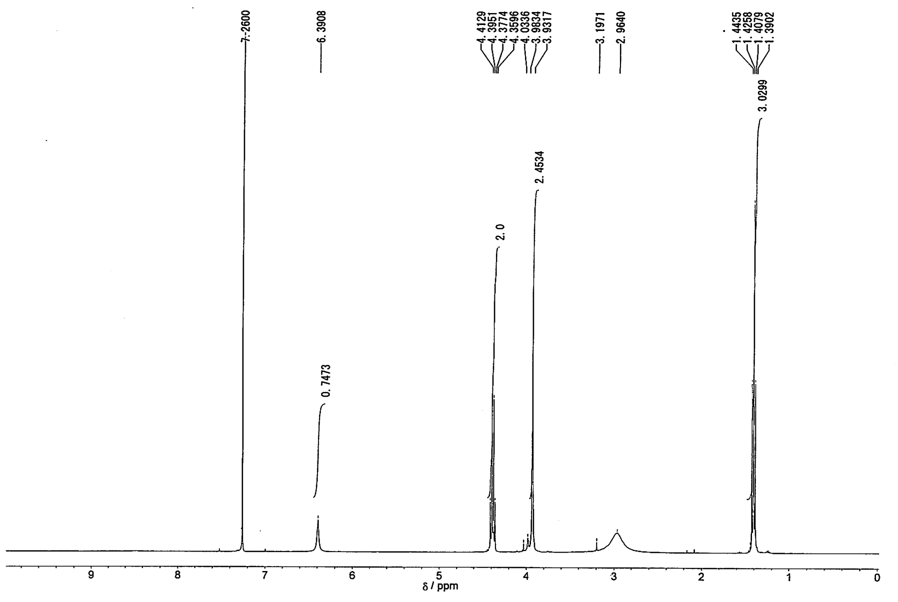

4-H_2NIm-OEt (**24**) ^{13}C-NMR ($CDCl_3$)

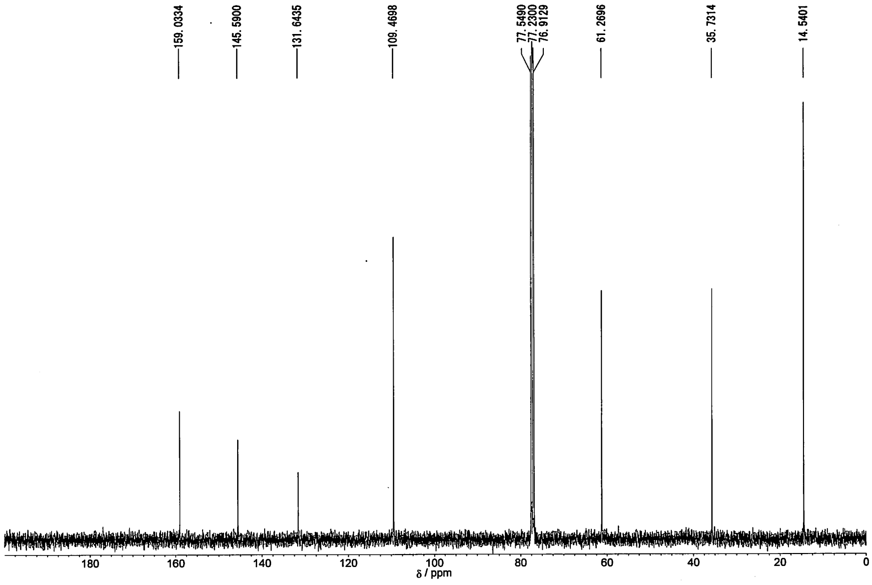

4-BocHNIm-OEt (**25**)

Elemental Composition Report

Single Mass Analysis (displaying only valid results)
Tolerance = 15.0 PPM / DBE: min = -1.5, max = 40.0
Isotope cluster parameters: Separation = 1.0 Abundance = 1.0%

Monoisotopic Mass, Odd and Even Electron Ions
12 formula(e) evaluated with 1 results within limits (all results (up to 1000) for each mass)

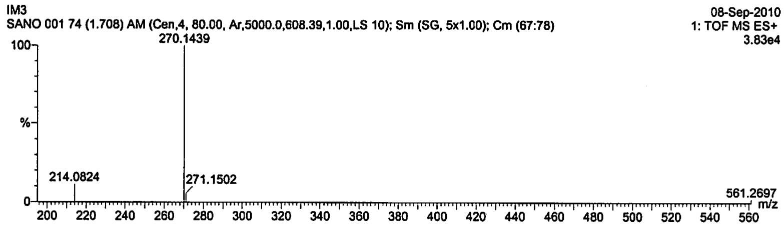

Minimum:				-1.5		
Maximum:		10.0	15.0	40.0		
Mass	Calc. Mass	mDa	PPM	DBE	Score	Formula
270.1439	270.1454	-1.5	-5.4	4.5	1	C12 H20 N3 O4

4-BocHNIm-OEt (**25**)

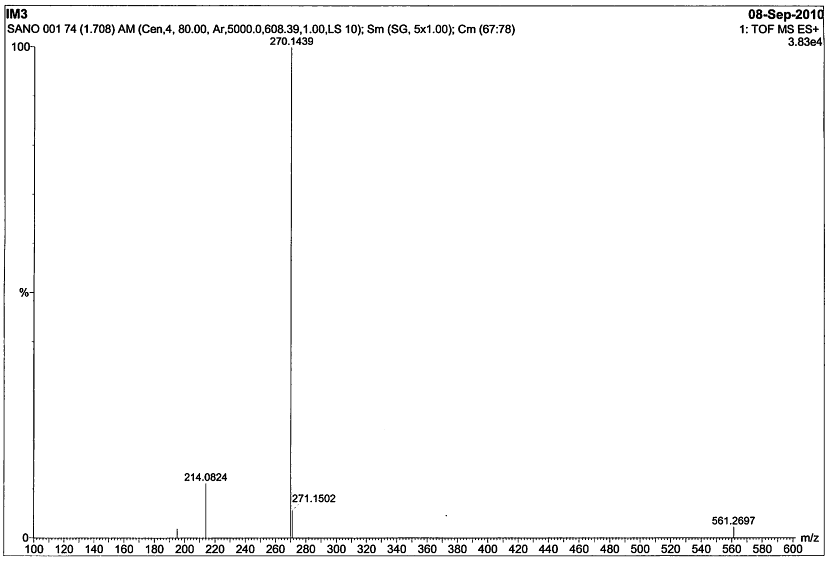

4-BocHNIm-OEt (**25**) ^{1}H-NMR (DMSO-d_6)

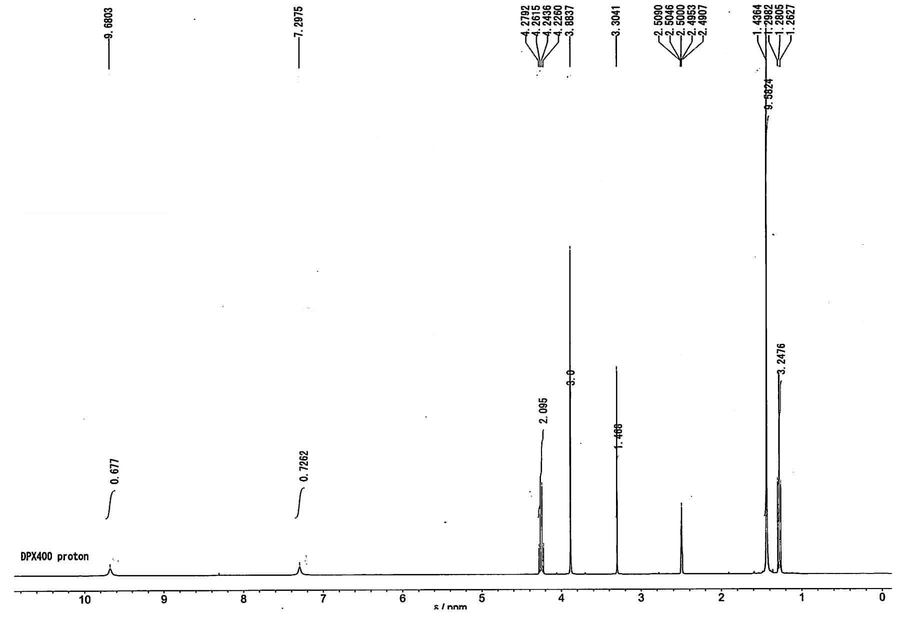

4-BocHNIm-OEt (**25**) ^{13}C-NMR (DMSO-d_6)

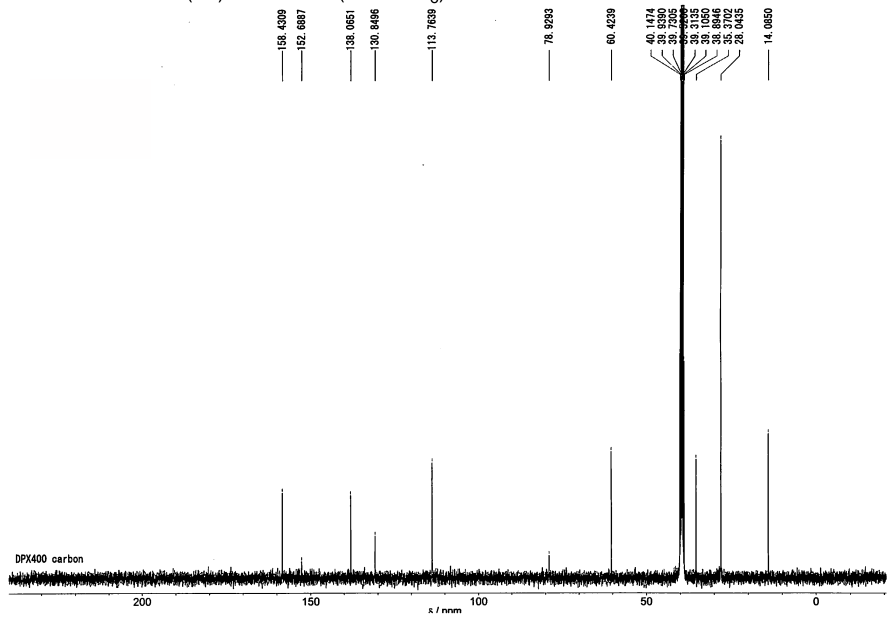

4-BocHNIm-OH (**26**)

Elemental Composition Report

Single Mass Analysis
Tolerance = 20.0 PPM / DBE: min = -1.5, max = 300.0
Element prediction: Off
Number of isotope peaks used for i-FIT = 3

Monoisotopic Mass, Even Electron Ions
3 formula(e) evaluated with 1 results within limits (up to 20 closest results for each mass)
Elements Used:
C: 1-300 H: 1-1000 N: 3-3 O: 4-4 Na: 1-1
aki-81
M-10182 78 (0.942) AM (Cen,4, 80.00, Ar,8500.0,556.28,0.00,LS 10); Sm (SG, 2x3.00); Sb (1,40.00); Cm (78:104) 1: TOF MS ES+ 3.91e+004

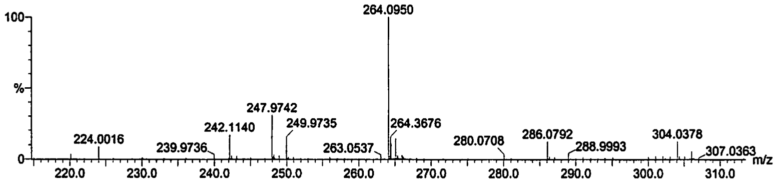

Minimum:				-1.5		
Maximum:		100.0	20.0	300.0		
Mass	Calc. Mass	mDa	PPM	DBE	i-FIT	Formula
264.0950	264.0960	-1.0	-3.8	4.5	70.3	C10 H15 N3 O4 Na

4-BocHNIm-OH (**26**)

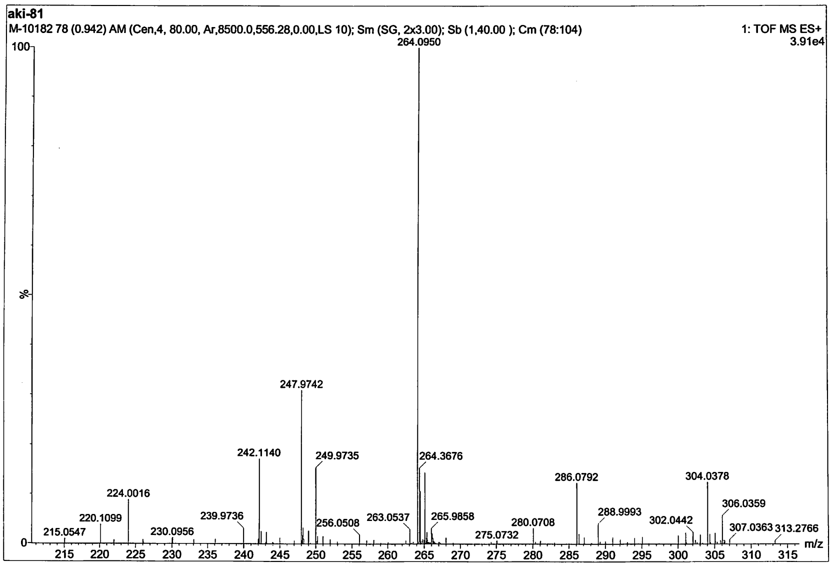

4-BocHNIm-OH (**26**) ^{1}H-NMR (DMSO-d$_6$)

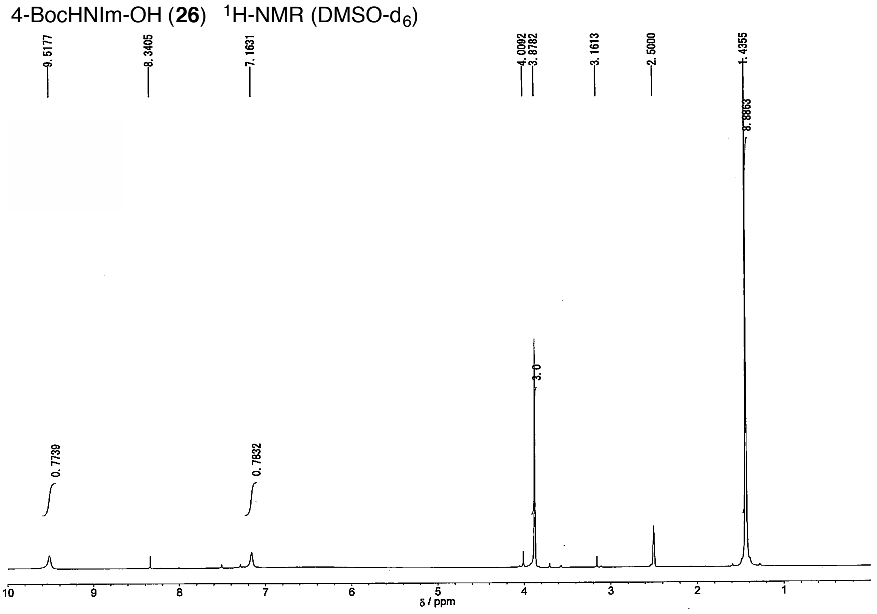

4-BocHNIm-OH (**26**) ^{13}C-NMR (DMSO-d$_6$)

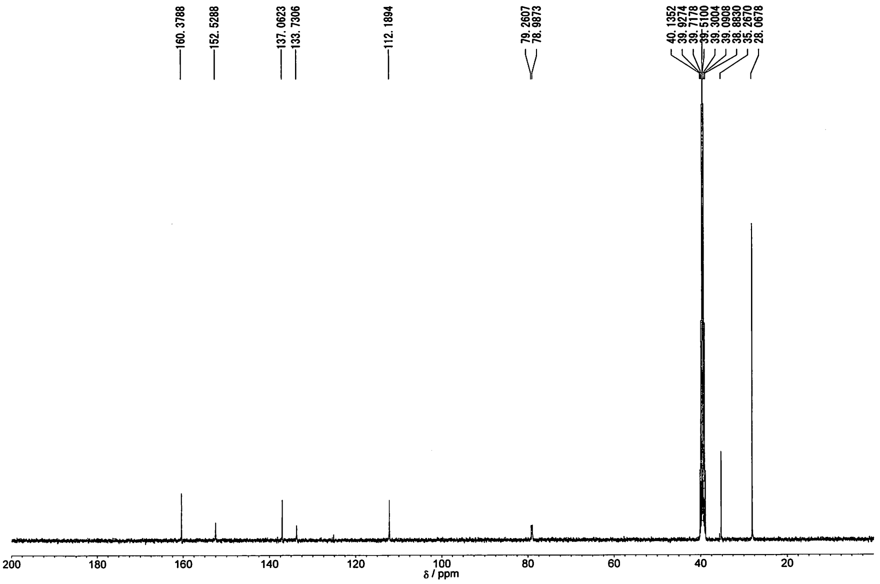

BocHNIm$_2$-OEt (**27**)

Elemental Composition Report

Single Mass Analysis (displaying only valid results)
Tolerance = 20.0 mDa / DBE: min = -0.5, max = 100.0
Isotope cluster parameters: Separation = 1.0 Abundance = 1.0%

Monoisotopic Mass, Odd and Even Electron Ions
4 formula(e) evaluated with 1 results within limits (all results (up to 1000) for each mass)

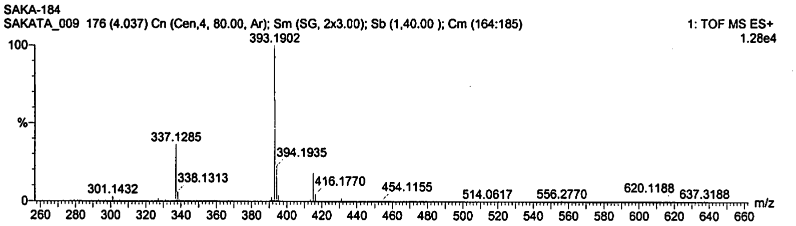

Mass	Calc. Mass	mDa	PPM	DBE	Score	Formula
Minimum:				-0.5		
Maximum:		20.0	50.0	100.0		
393.1902	393.1886	1.5	3.8	8.5	1	C17 H25 N6 O5

BocHNIm$_2$-OEt (**27**)

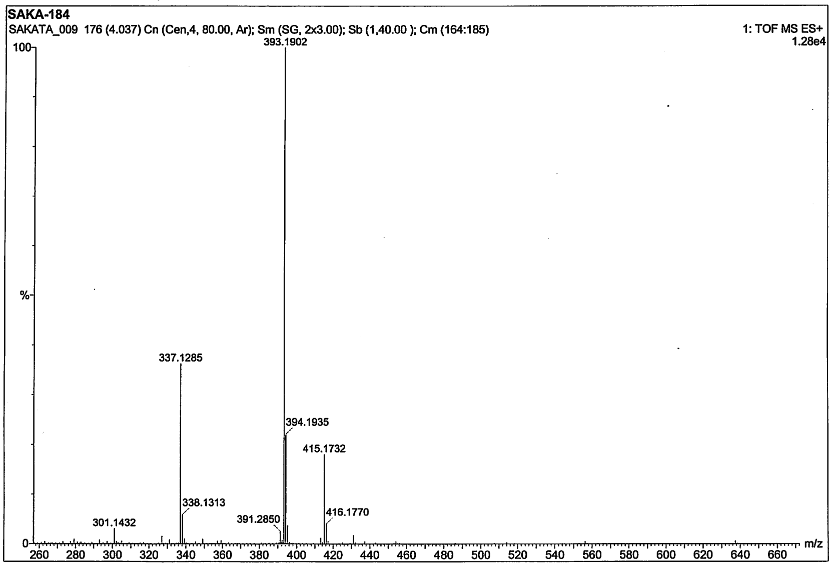

BocHNIm$_2$-OEt (**27**) ^{1}H-NMR ($CDCl_3$)

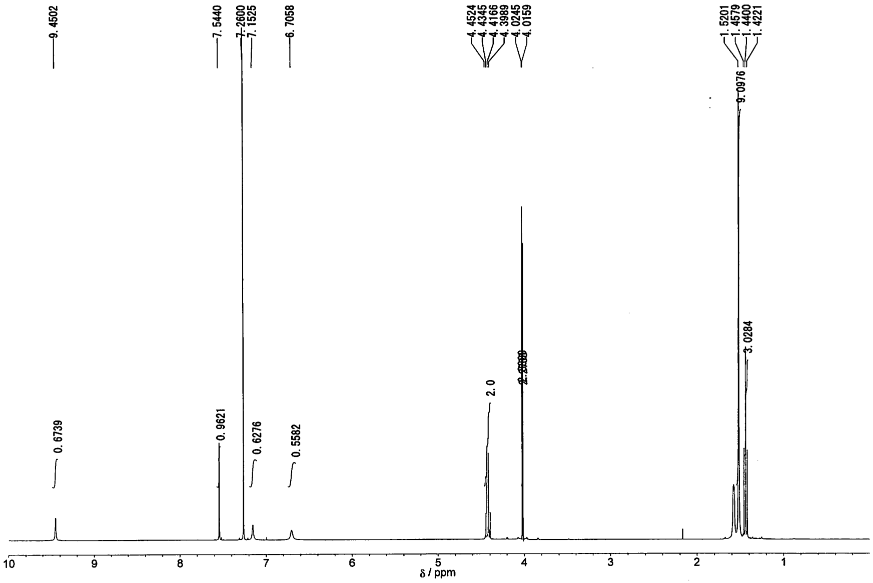

BocHNIm$_2$-OEt (**27**) ^{13}C-NMR ($CDCl_3$)

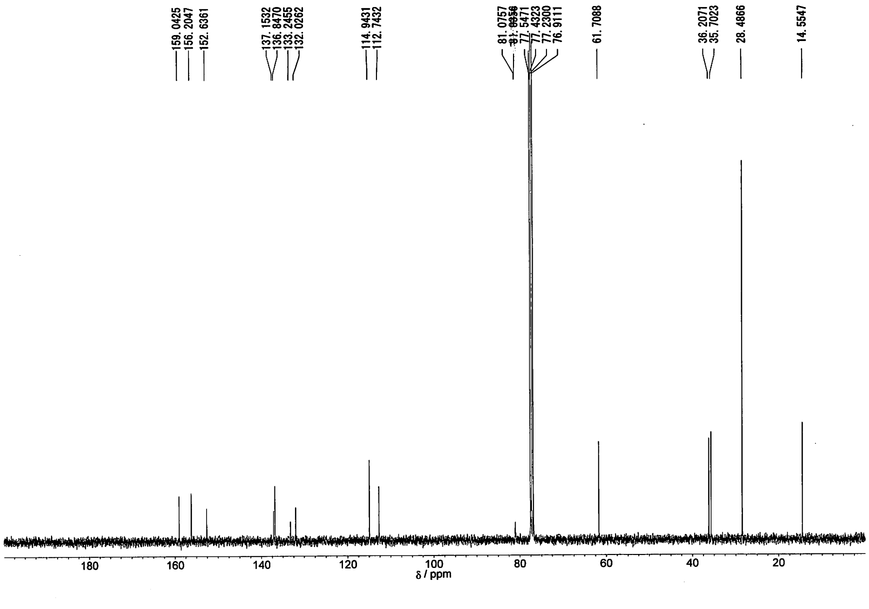

• H_2NIm_2-OEt

Elemental Composition Report

Single Mass Analysis (displaying only valid results)
Tolerance = 20.0 PPM / DBE: min = -0.5, max = 100.0
Isotope cluster parameters: Separation = 1.0 Abundance = 1.0%

Monoisotopic Mass, Odd and Even Electron Ions
31 formula(e) evaluated with 1 results within limits (all results (up to 1000) for each mass)

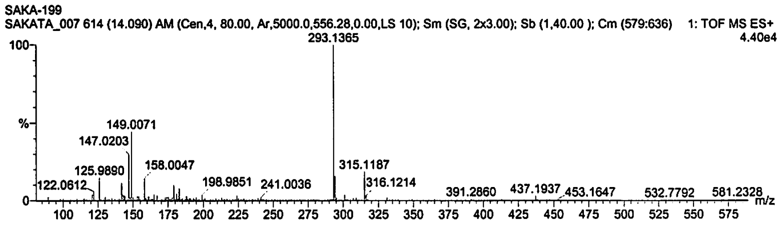

Minimum:				-0.5		
Maximum:		10.0	20.0	100.0		
Mass	Calc. Mass	mDa	PPM	DBE	Score	Formula
293.1365	293.1362	0.3	1.0	7.5	1	C12 H17 N6 O3

H_2NIm_2-OEt

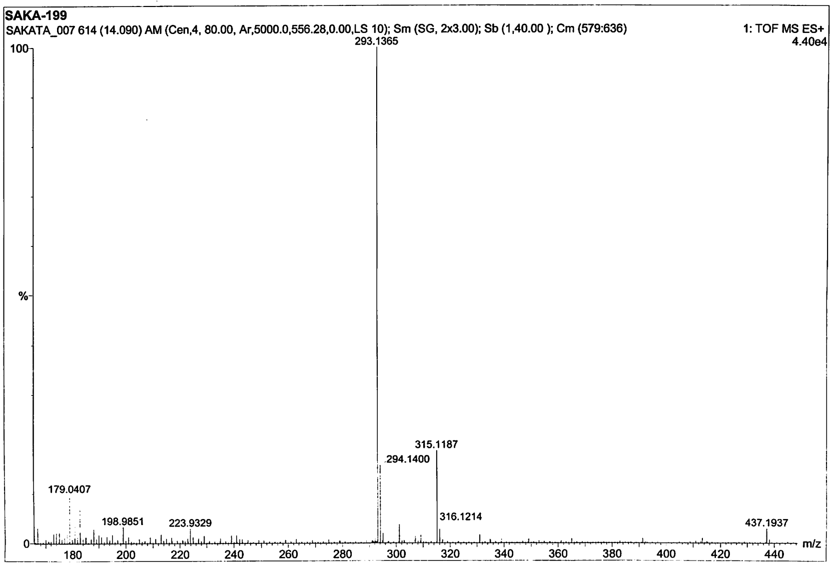

H_2NIm_2-OEt ^{1}H-NMR (DMSO-d_6)

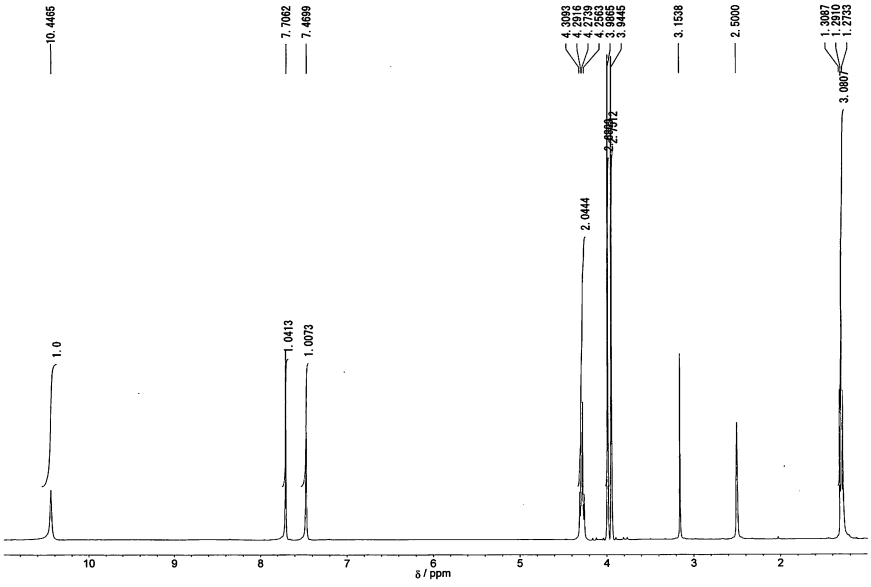

H_2NIm_2-OEt ^{13}C-NMR (DMSO-d_6)

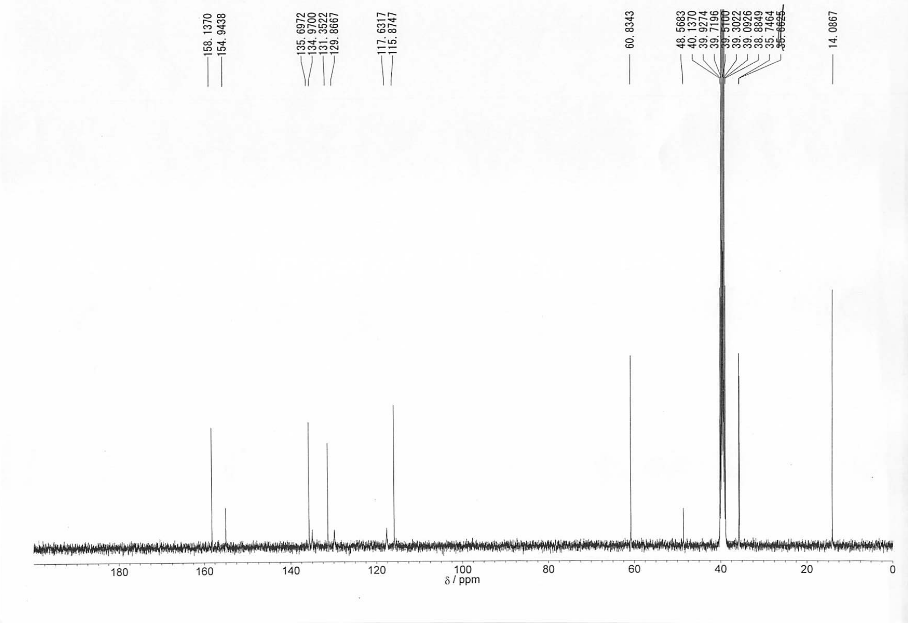

Im$_3$-OEt (**28**)

Elemental Composition Report

Single Mass Analysis (displaying only valid results)
Tolerance = 5.0 PPM / DBE: min = -0.5, max = 100.0
Isotope cluster parameters: Separation = 1.0 Abundance = 1.0%

Monoisotopic Mass, Even Electron Ions
42 formula(e) evaluated with 1 results within limits (all results (up to 1000) for each mass)

SAKA-194
SAKATA_008 599 (13.741) AM (Cen,4, 80.00, Ar,5000.0,556.28,0.00,LS 10); Sm (SG, 2x3.00); Sb (1,40.00); Cm (581:611) 1: TOF MS ES+ 2.85e4

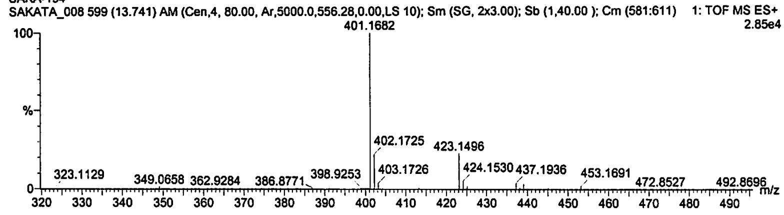

Minimum:				-0.5		
Maximum:		10.0	5.0	100.0		
Mass	Calc. Mass	mDa	PPM	DBE	Score	Formula
401.1682	401.1686	-0.4	-0.9	11.5	1	C17 H21 N8 O4

Im$_3$-OEt (**28**)

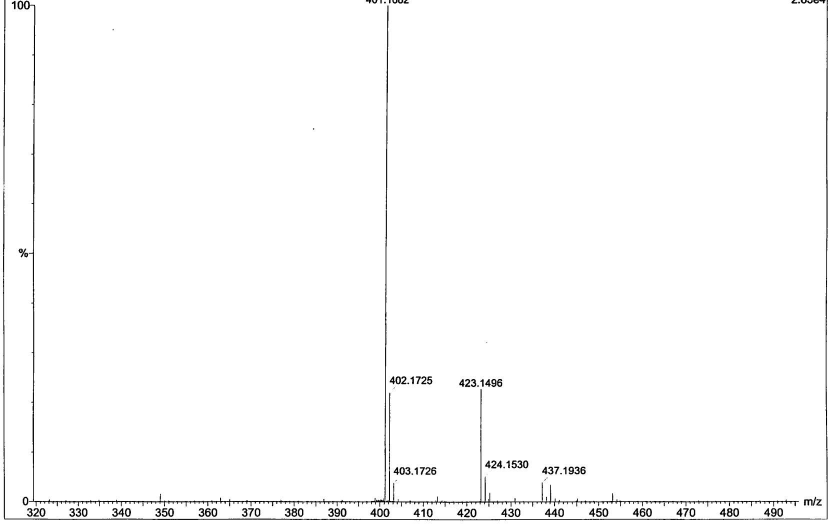

Im$_3$-OEt (**28**) ^{1}H-NMR ($CDCl_3$)

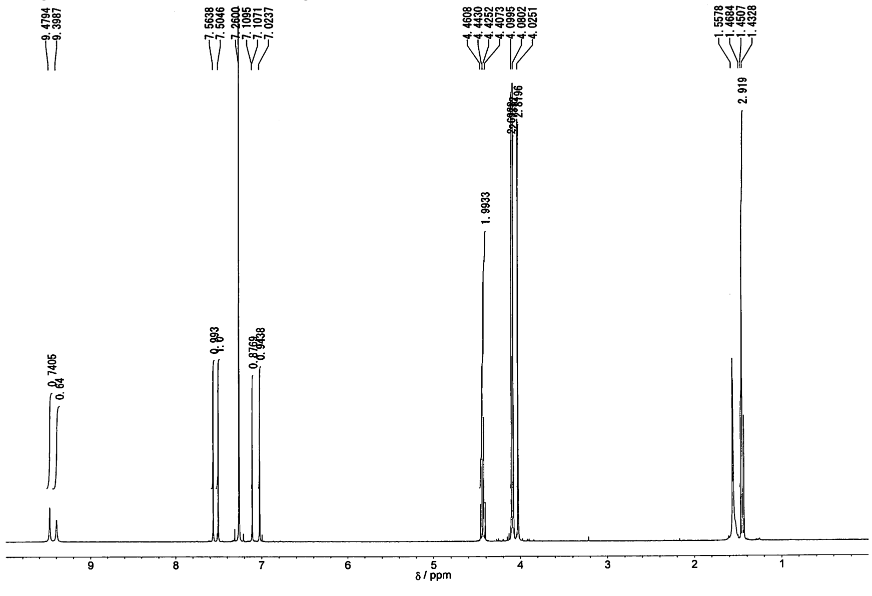

Im$_3$-OEt (**28**) ^{13}C-NMR ($CDCl_3$)

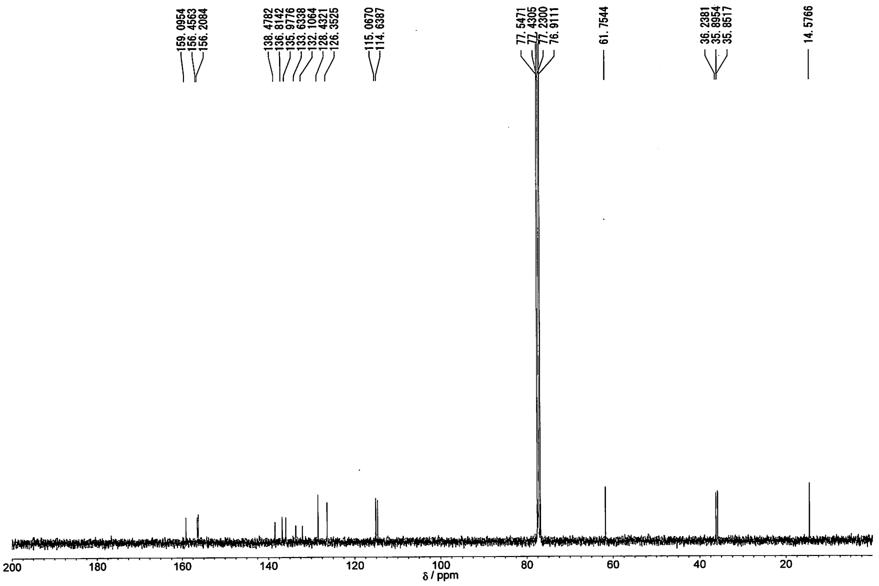

Im$_3$-OH (carboxylic acid)

Elemental Composition Report

Single Mass Analysis

Tolerance = 1000.0 mDa / DBE: min = 0.5, max = 500.0

Element prediction: Off

Number of isotope peaks used for i-FIT = 4

Monoisotopic Mass, Even Electron Ions

1 formula(e) evaluated with 1 results within limits (all results (up to 1000) for each mass)

Elements Used:

C: 0-15 H: 0-17 N: 8-8 O: 4-4

saka-224

SAKATA 009 93 (1.116) Cn (Cen,4, 80.00, Ar); Sm (SG, 2x3.00); Cm (71:113)   1: TOF MS ES+ 1.82e+005

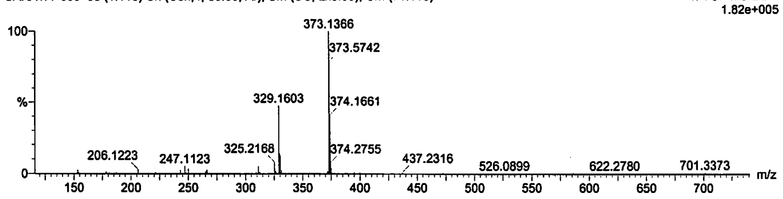

	Mass	Calc. Mass	mDa	PPM	DBE	i-FIT	Formula
Minimum:					0.5		
Maximum:			1000.0	20.0	500.0		
	[illegible]	[illegible]	[illegible]	[illegible]	[illegible]	[illegible]	[illegible]

Im$_3$-OH (carboxylic acid)

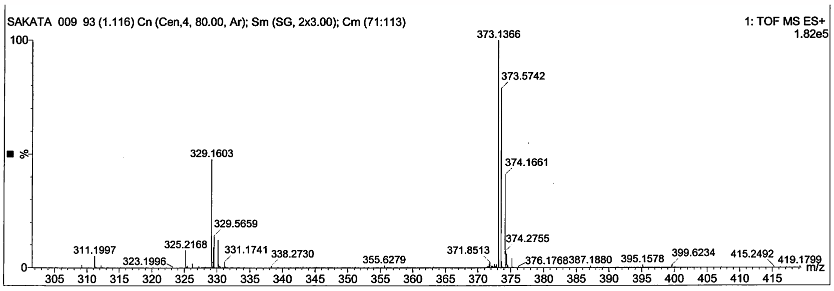

Im$_3$-OH (carboxylic acid) ^{1}H-NMR (DMSO-d$_6$)

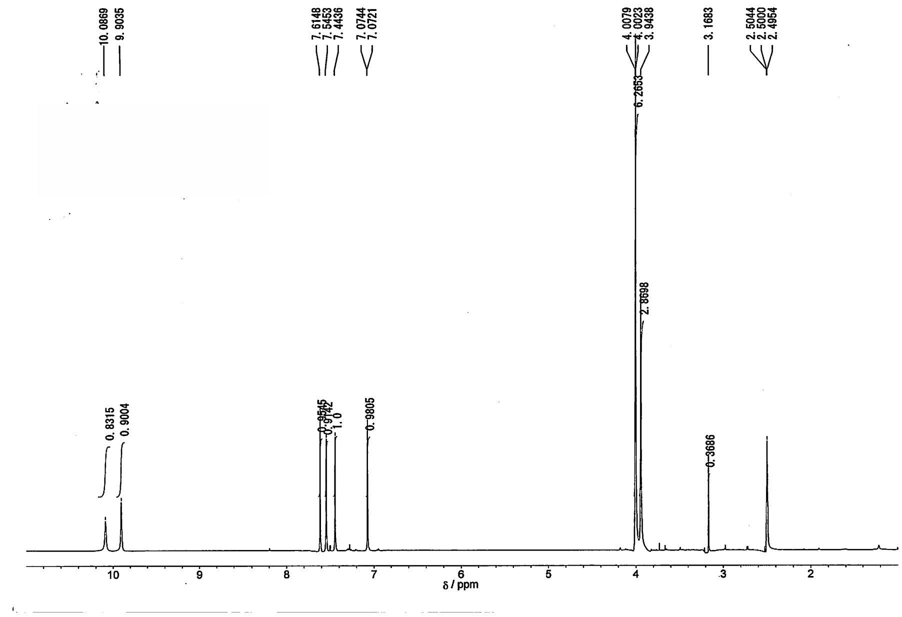

Im$_3$-OH (carboxylic acid) ^{13}C-NMR (DMSO-d$_6$)

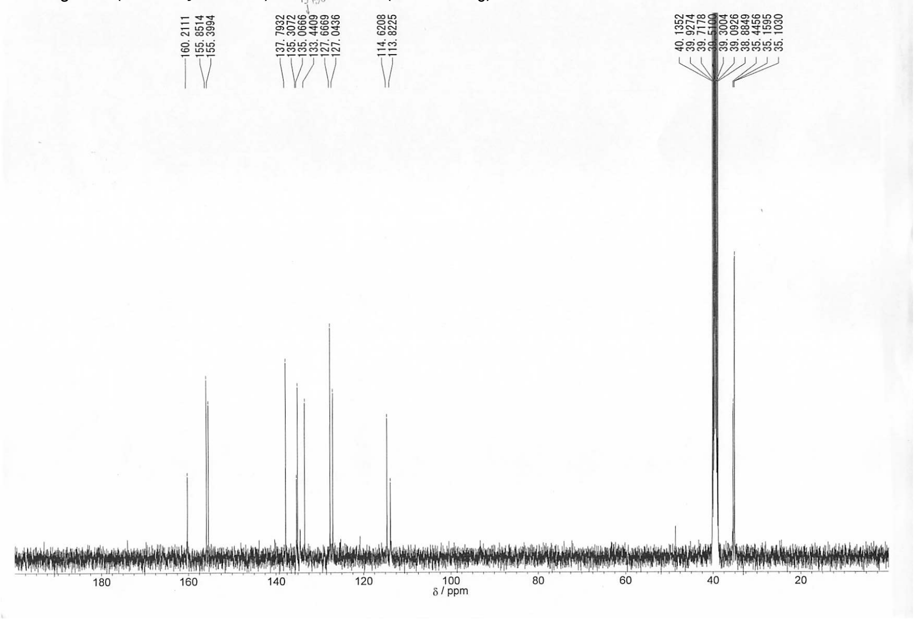

Im$_3$-NH(CH$_2$)$_3$NHFmoc (**29**)

Elemental Composition Report

Single Mass Analysis (displaying only valid results)

Tolerance = 20.0 mDa / DBE: min = -0.5, max = 100.0

Isotope cluster parameters: Separation = 1.0 Abundance = 1.0%

Monoisotopic Mass, Odd and Even Electron Ions

7 formula(e) evaluated with 1 results within limits (all results (up to 1000) for each mass)

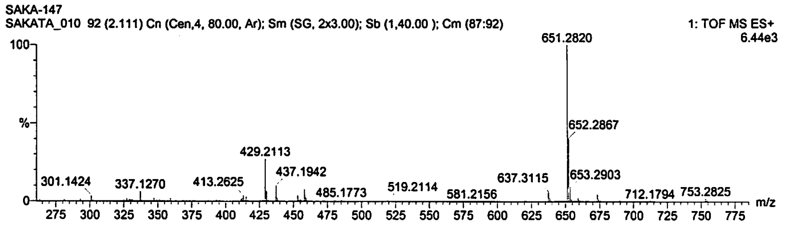

Minimum:				-0.5		
Maximum:		20.0	50.0	100.0		
Mass	Calc. Mass	mDa	PPM	DBE	Score	Formula
651.2820	651.2792	2.9	4.4	21.5	1	C33 H35 N10 O5

Im$_3$-NH(CH$_2$)$_3$NHFmoc (**29**)

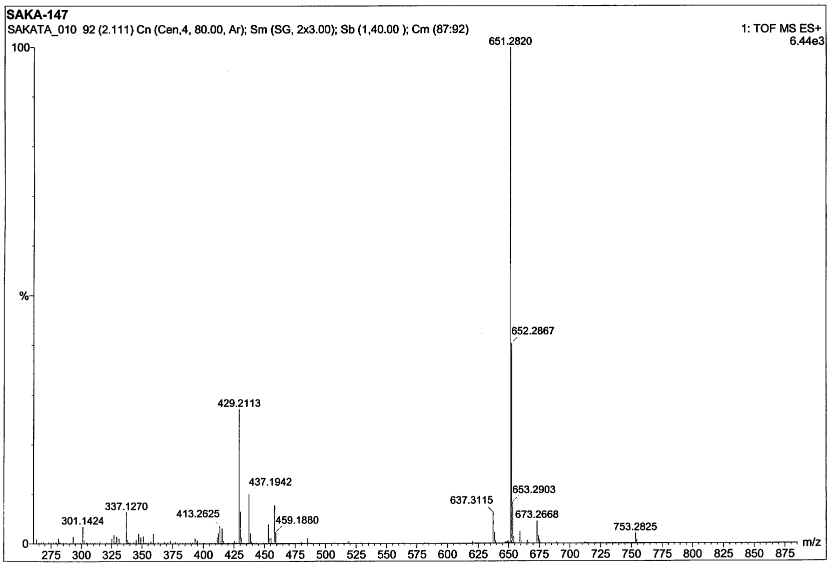

Im$_3$-NH(CH$_2$)$_3$NHFmoc (**29**) ^{1}H-NMR (DMSO-d$_6$)

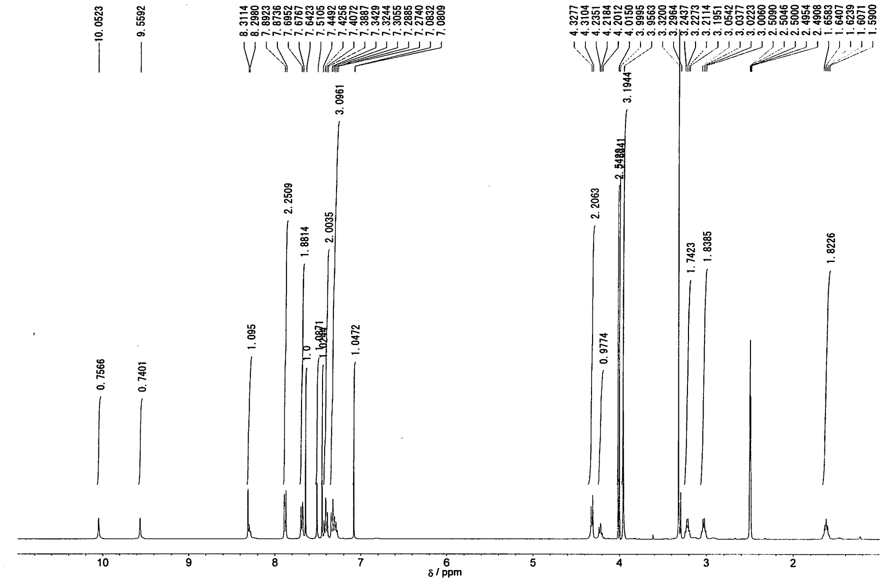

Im$_3$-NH(CH$_2$)$_3$NHFmoc (**29**) ^{13}C-NMR (CDCl$_3$)

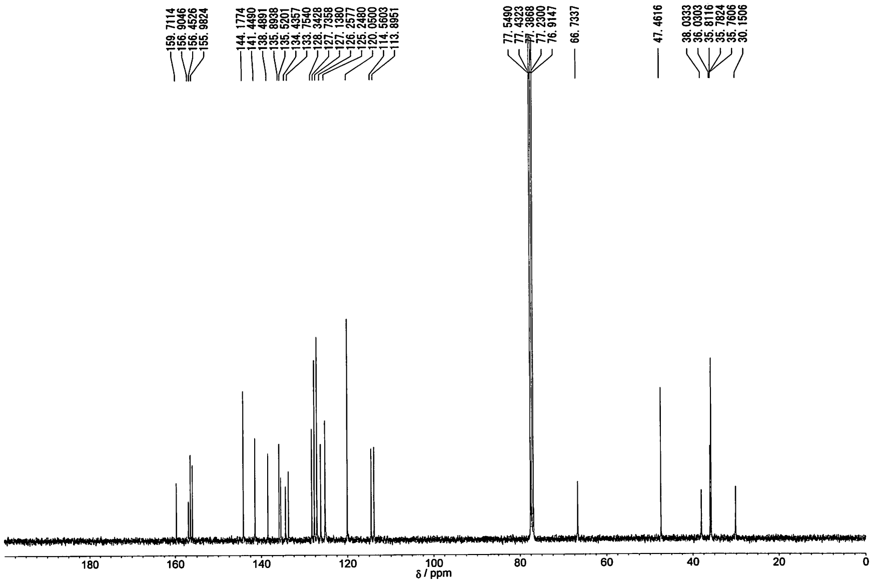

Im_3-NH(CH_2)$_4$NHFmoc (**30**)

Elemental Composition Report

Single Mass Analysis (displaying only valid results)

Tolerance = 20.0 mDa / DBE: min = -0.5, max = 100.0

Isotope cluster parameters: Separation = 1.0 Abundance = 1.0%

Monoisotopic Mass, Odd and Even Electron Ions

7 formula(e) evaluated with 1 results within limits (all results (up to 1000) for each mass)

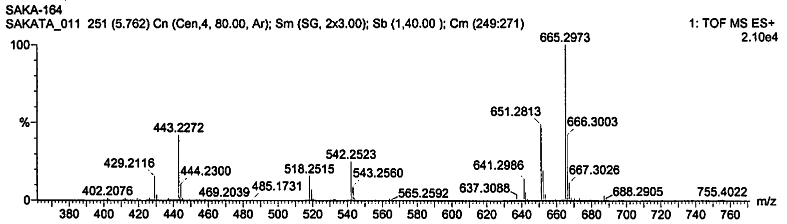

Minimum:				-0.5		
Maximum:		20.0	50.0	100.0		
Mass	Calc. Mass	mDa	PPM	DBE	Score	Formula
665.2973	665.2948	2.5	3.7	21.5	1	C34 H37 N10 O5

Im_3-NH(CH_2)$_4$NHFmoc (**30**)

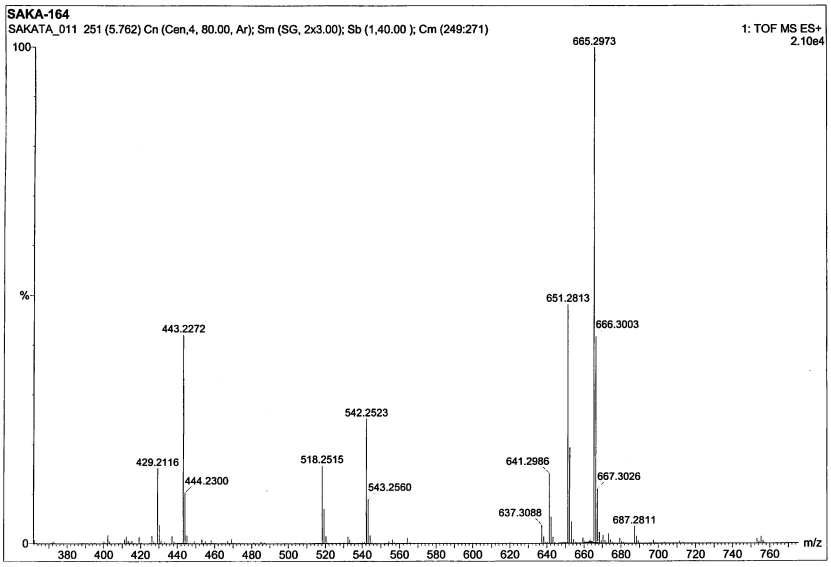

Im$_3$-NH(CH$_2$)$_4$NHFmoc (**30**) ^{1}H-NMR (DMSO-d$_6$)

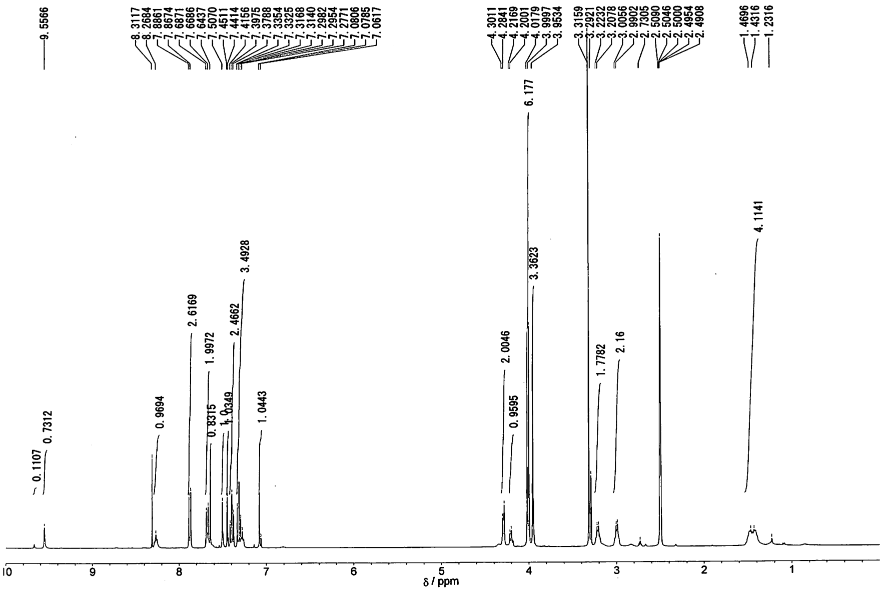

Im$_3$-NH(CH$_2$)$_4$NHFmoc (**30**) ^{13}C-NMR (CDCl$_3$)

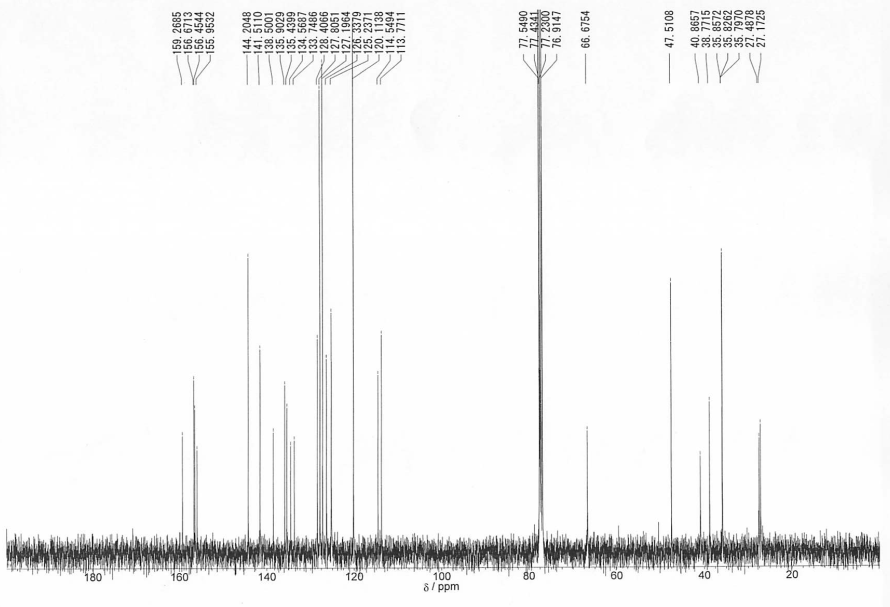

Im$_3$-NH(CH$_2$)$_5$NHFmoc (**31**)

Elemental Composition Report

Single Mass Analysis (displaying only valid results)

Tolerance = 20.0 mDa / DBE: min = -0.5, max = 100.0

Isotope cluster parameters: Separation = 1.0 Abundance = 1.0%

Monoisotopic Mass, Odd and Even Electron Ions

7 formula(e) evaluated with 1 results within limits (all results (up to 1000) for each mass)

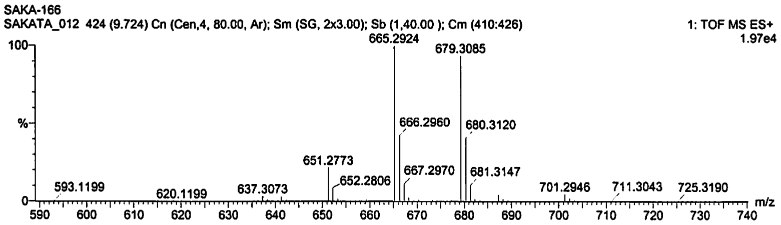

	Calc. Mass	mDa	PPM	DBE	Score	Formula
Minimum:				-0.5		
Maximum:		20.0	50.0	100.0		
Mass	Calc. Mass	mDa	PPM	DBE	Score	Formula
679.3085	679.3105	-2.0	-3.0	21.5	1	C35 H39 N10 O5

Im$_3$-NH(CH$_2$)$_5$NHFmoc (**31**)

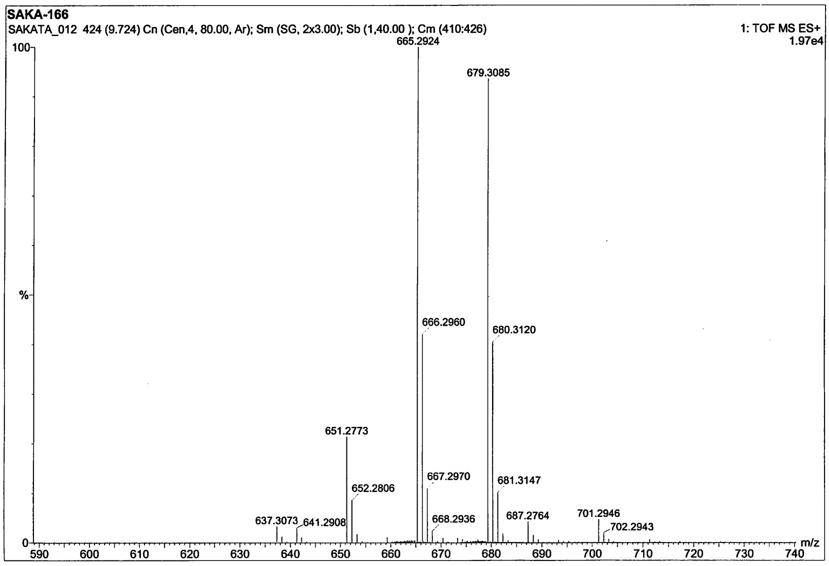

Im$_3$-NH(CH$_2$)$_5$NHFmoc (**31**) ^{1}H-NMR (DMSO-d$_6$)

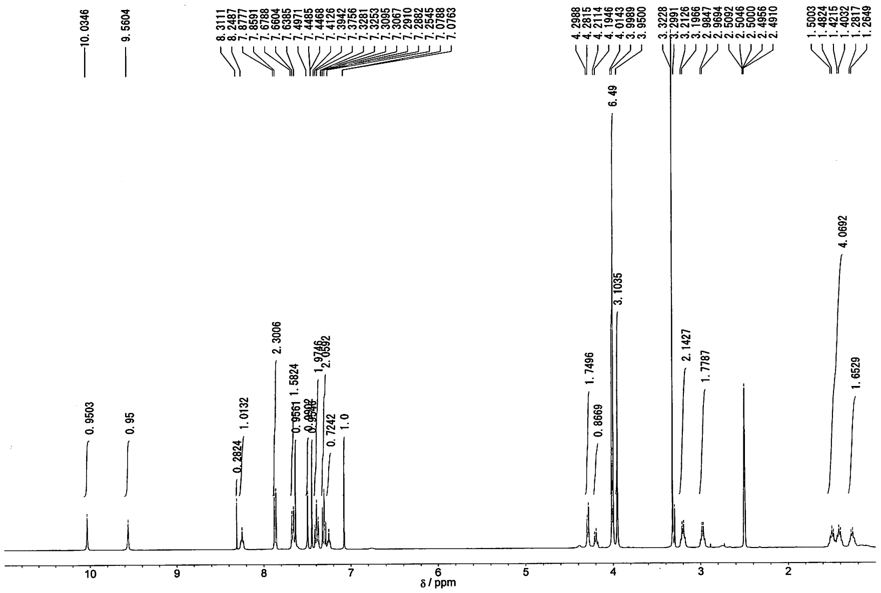

Im$_3$-NH(CH$_2$)$_5$NHFmoc (**31**) ^{13}C-NMR (CDCl$_3$)

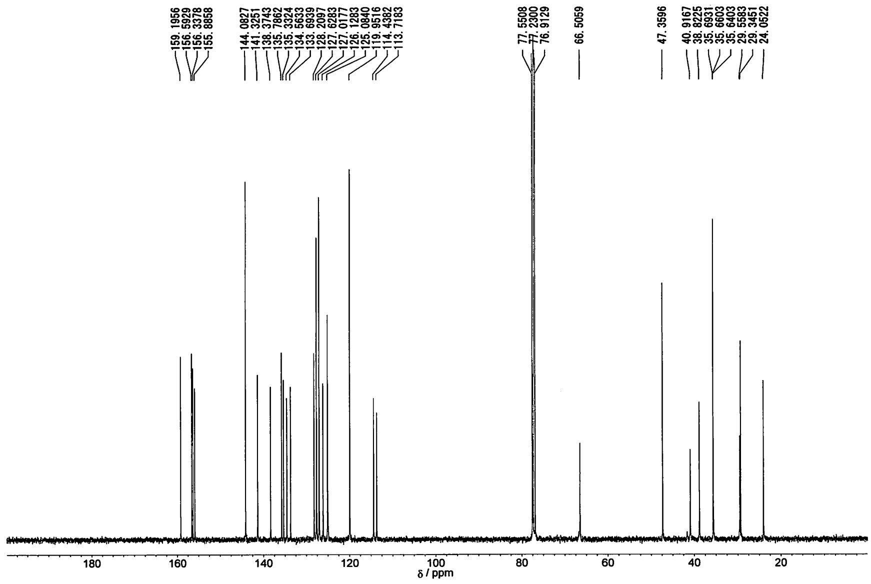

Py$_3$-NH(CH$_2$)$_3$CO$_2$Et (**32**)

Elemental Composition Report

Single Mass Analysis
Tolerance = 20.0 PPM / DBE: min = -1.5, max = 300.0
Element prediction: Off
Number of isotope peaks used for i-FIT = 3

Monoisotopic Mass, Even Electron Ions
5 formula(e) evaluated with 1 results within limits (up to 20 closest results for each mass)
Elements Used:
C: 1-300 H: 1-1000 N: 6-6 O: 5-5
yu-Py3GABA
M-8434 98 (1.167) AM (Cen,4, 80.00, Ar,8500.0,556.28,0.00,LS 10); Sm (SG, 2x3.00); Sb (1,40.00); Cm (95:129) 1: TOF MS ES+ 1.52e+004

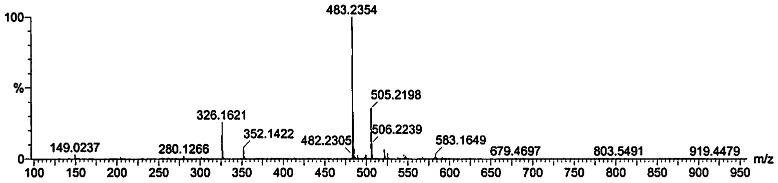

Mass	Calc. Mass	mDa	PPM	DBE	i-FIT	Formula
Minimum:				-1.5		
Maximum:		100.0	20.0	300.0		
483.2354	483.2356	-0.2	-0.4	12.5	62.6	C24 H31 N6 O5

Py$_3$-NH(CH$_2$)$_3$CO$_2$Et (**32**)

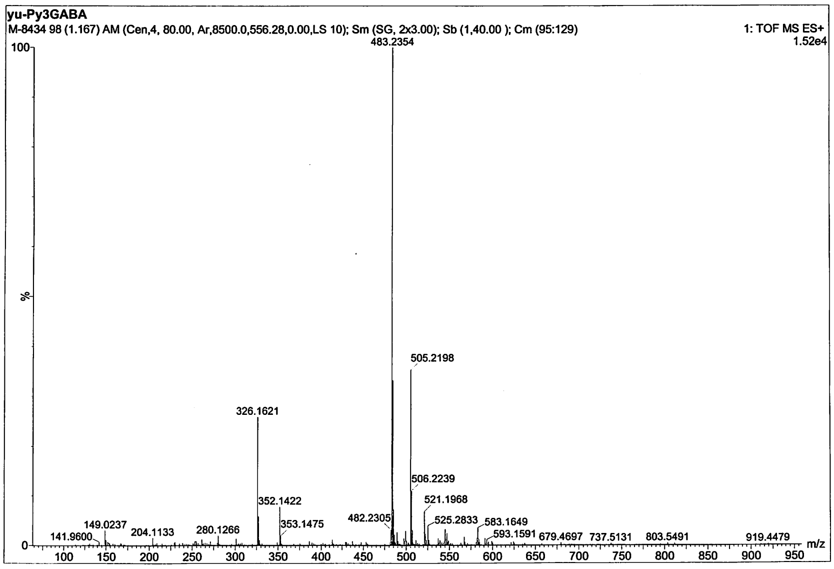

Py$_3$-NH(CH$_2$)$_3$CO$_2$Et (**32**) ^{1}H-NMR (CDCl$_3$)

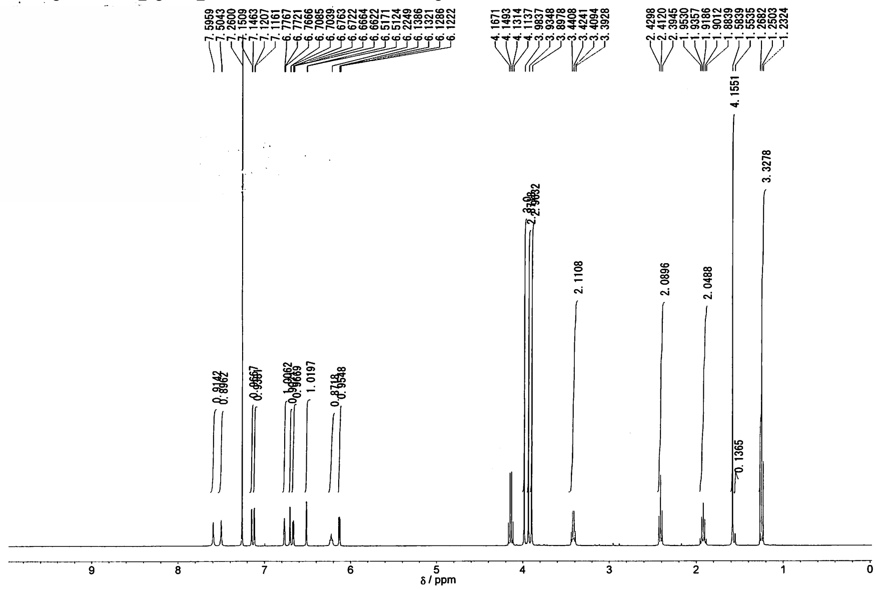

Py$_3$-NH(CH$_2$)$_3$CO$_2$Et (**32**) ^{13}C-NMR (CDCl$_3$)

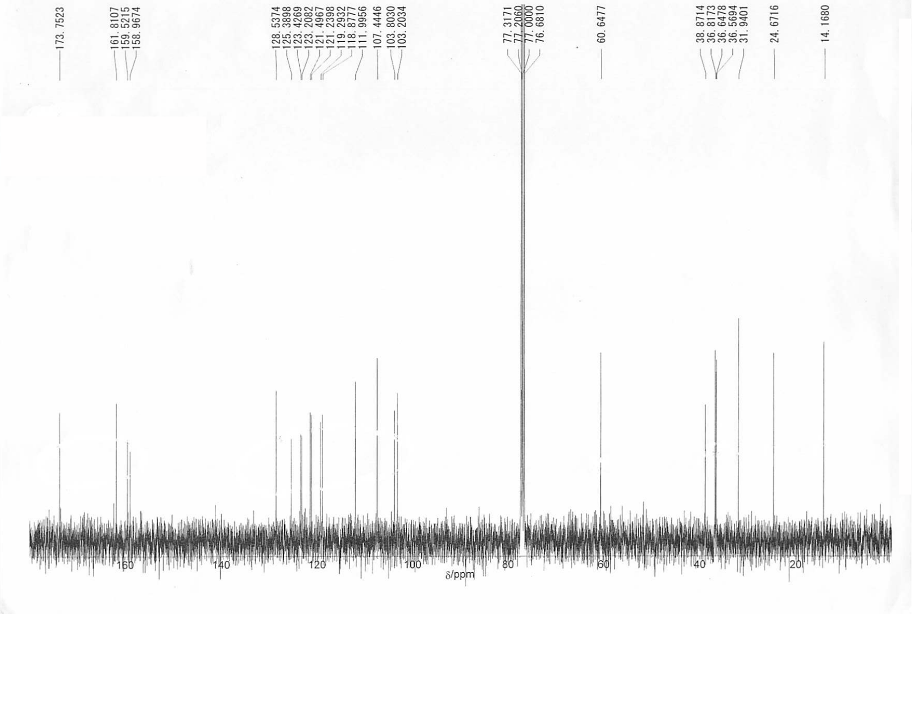

Py$_3$-NH(CH$_2$)$_3$CO$_2$H

Elemental Composition Report

Single Mass Analysis
Tolerance = 20.0 PPM / DBE: min = 0.5, max = 500.0
Element prediction: Off
Number of isotope peaks used for i-FIT = 4

Monoisotopic Mass, Even Electron Ions
5 formula(e) evaluated with 1 results within limits (all results (up to 1000) for each mass)
Elements Used:
C: 1-300 H: 1-1000 N: 6-6 O: 5-5
koga-156
KOGAWARA_002 44 (0.537) Cn (Cen,4, 80.00, Ar); Sm (SG, 2x3.00); Cm (2:44)

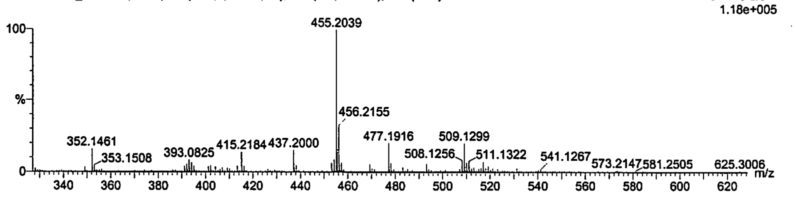

Mass	Calc. Mass	mDa	PPM	DBE	i-FIT	Formula
Minimum:				0.5		
Maximum:		100.0	20.0	500.0		
455.2039	455.2043	-0.4	-0.9	12.5	1368.3	C22 H27 N6 O5

Py$_3$-NH(CH$_2$)$_3$CO$_2$H

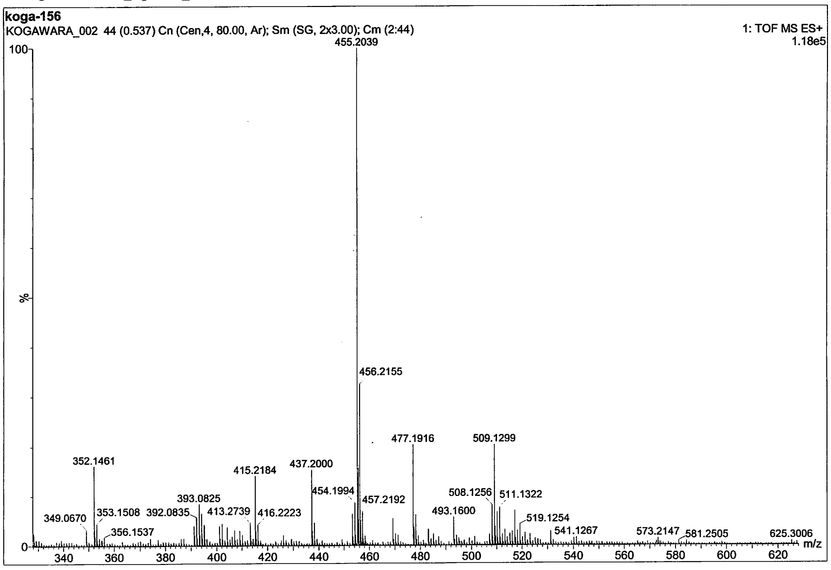

Py$_3$-NH(CH$_2$)$_3$CO$_2$H ^{1}H-NMR (DMSO-d$_6$)

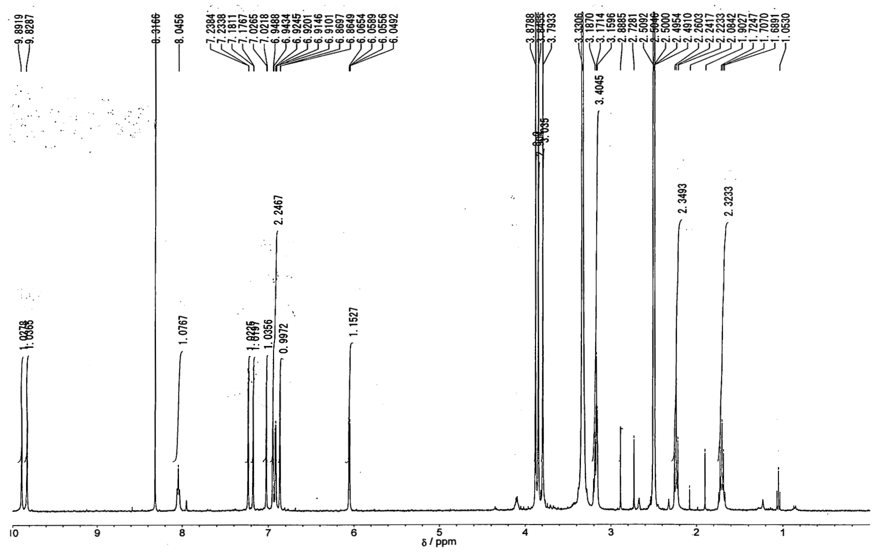

Py$_3$-NH(CH$_2$)$_3$CO$_2$H ^{13}C-NMR (DMSO-d$_6$)

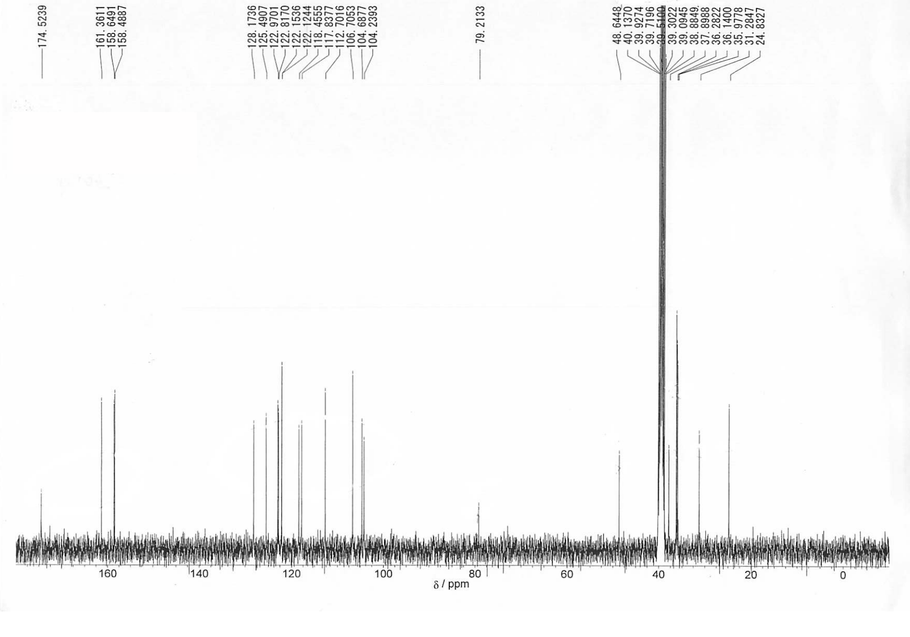

BocHNIm$_3$-OEt (**33**)

Elemental Composition Report

Single Mass Analysis
Tolerance = 20.0 PPM / DBE: min = 0.5, max = 500.0
Element prediction: Off
Number of isotope peaks used for i-FIT = 4

Monoisotopic Mass, Even Electron Ions
8 formula(e) evaluated with 1 results within limits (all results (up to 1000) for each mass)
Elements Used:
C: 1-300 H: 1-1000 N: 9-9 O: 6-6 127I: 0-1

koga-147
OGASAHARA_023 82 (0.982) Cn (Cen,4, 80.00, Ar); Sm (SG, 2x3.00); Cm (71:112)

1: TOF MS ES+
1.97e+004

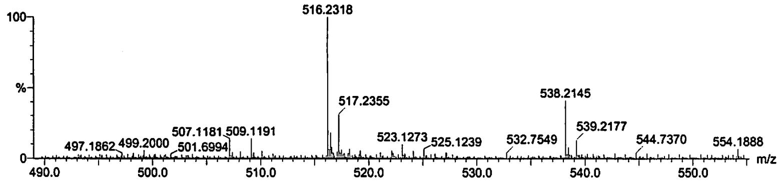

	Mass	Calc. Mass	mDa	PPM	DBE	i-FIT	Formula
Minimum:					0.5		
Maximum:			100.0	20.0	500.0		
	516.2318	516.2319	-0.1	-0.2	12.5	523.7	C22 H30 N9 O6

BocHNIm$_3$-OEt (**33**)

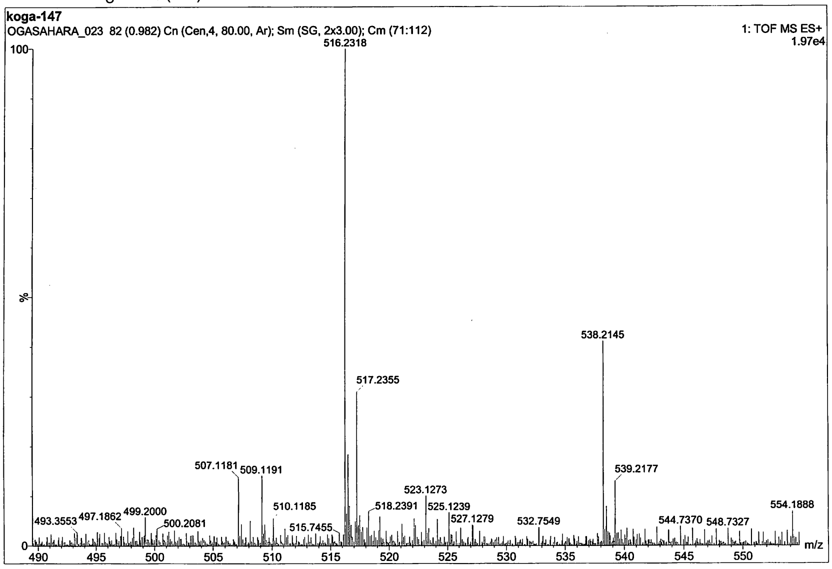

BocHNIm$_3$-OEt (**33**) ^{1}H-NMR ($CDCl_3$)

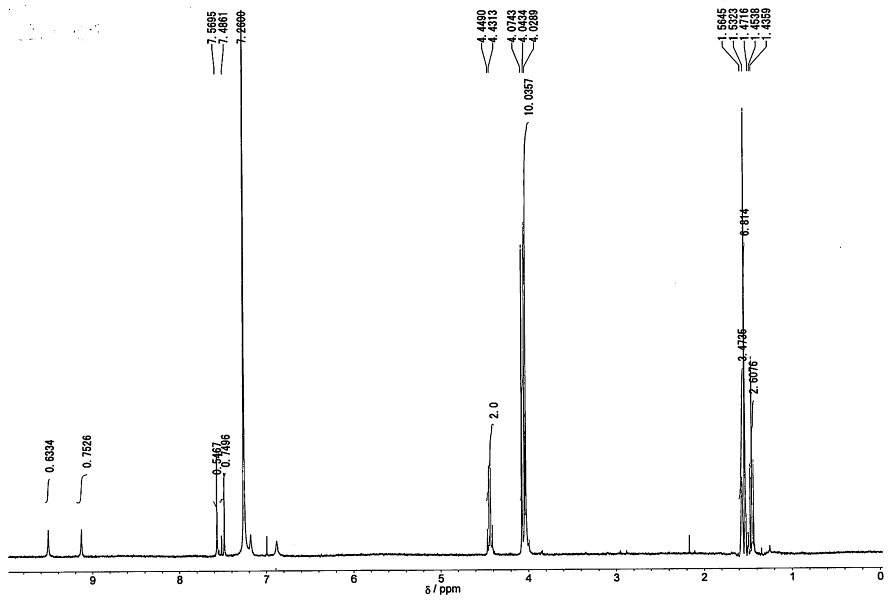

BocHNIm$_3$-OEt (**33**) ^{13}C-NMR ($CDCl_3$)

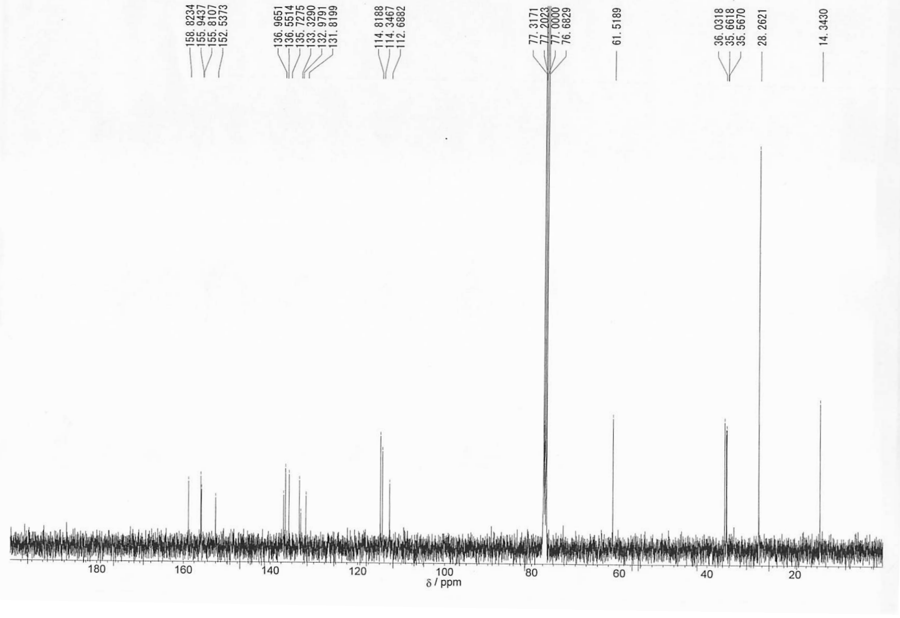

H_2NIm_3-OEt

Elemental Composition Report

Single Mass Analysis

Tolerance = 10.0 mDa / DBE: min = -1.5, max = 300.0
Element prediction: Off
Number of isotope peaks used for i-FIT = 3

Monoisotopic Mass, Even Electron Ions
4 formula(e) evaluated with 1 results within limits (up to 50 closest results for each mass)
Elements Used:
C: 0-300 H: 0-1000 N: 9-9 O: 4-4

NH2Im3OEt
M-12011 192 (1.549) AM2 (Ar,22000.0,0.00,0.00); ABS; Cm (192:213)

1: TOF MS ES+
8.81e+007

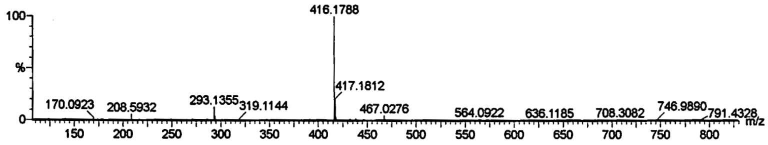

Minimum:				-1.5				
Maximum:		10.0	20.0	300.0				
Mass	Calc. Mass	mDa	PPM	DBE	i-FIT	Norm	Conf(%)	Formula
416.1788	416.1795	-0.7	-1.7	11.5	724.1	n/a	n/a	C17 H22 N9 O4

H_2NIm_3-OEt

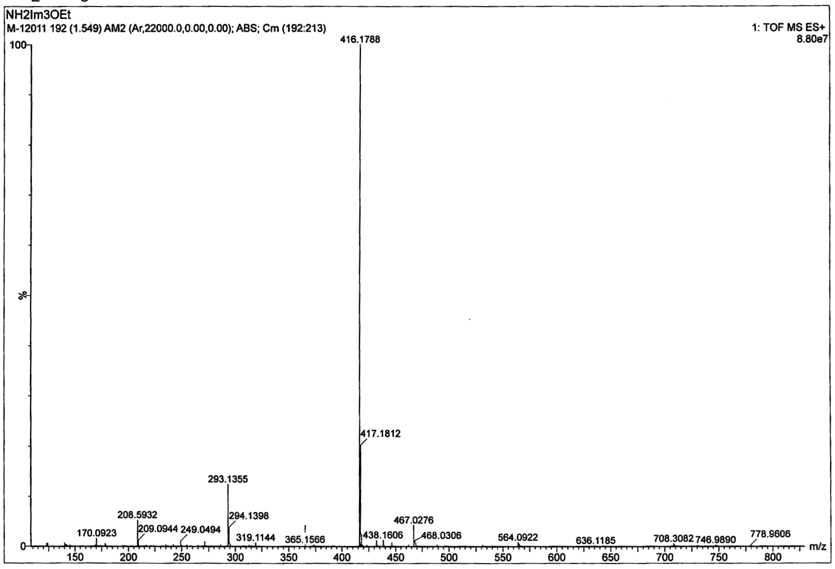

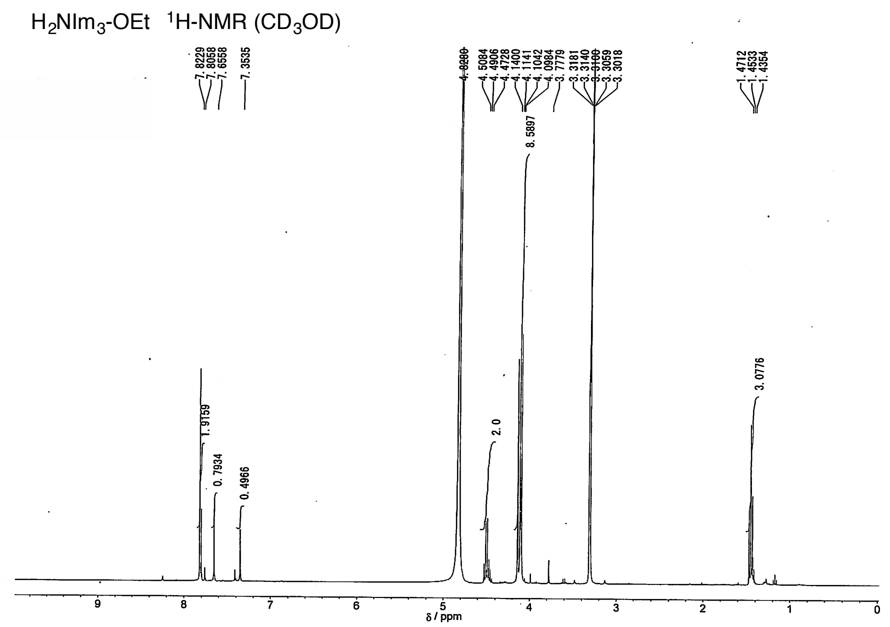
H_2NIm_3-OEt ^1H-NMR (CD_3OD)
δ / ppm

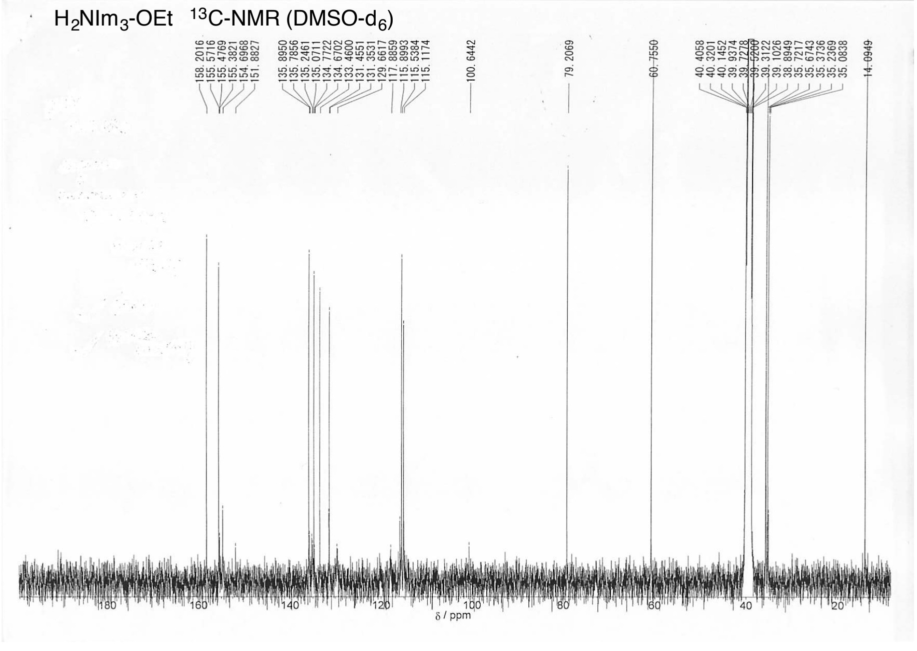
H_2NIm_3-OEt ^{13}C-NMR (DMSO-d_6)
δ / ppm

Py$_3$-γ-Im$_3$-OEt (**34**)

Elemental Composition Report

Single Mass Analysis

Tolerance = 20.0 PPM / DBE: min = -1.5, max = 300.0

Element prediction: Off

Number of isotope peaks used for i-FIT = 3

Monoisotopic Mass, Even Electron Ions

8 formula(e) evaluated with 1 results within limits (up to 20 closest results for each mass)

Elements Used:

C: 1-300 H: 1-1000 N: 15-15 O: 8-8

yu-199cru

M-7935-1 79 (0.951) AM (Cen,4, 80.00, Ar,8500.0,556.28,0.00,LS 10); Sm (SG, 2x3.00); Sb (1,40.00); Cm (79:119) 1: TOF MS ES+ 1.05e+005

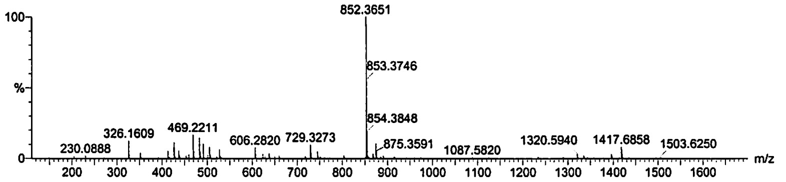

Minimum:				-1.5		
Maximum:		100.0	20.0	300.0		
Mass	Calc. Mass	mDa	PPM	DBE	i-FIT	Formula
852.3651	852.3654	-0.3	-0.4	24.5	758.9	C39 H46 N15 O8

Py$_3$-γ–Im$_3$-OEt (**34**)

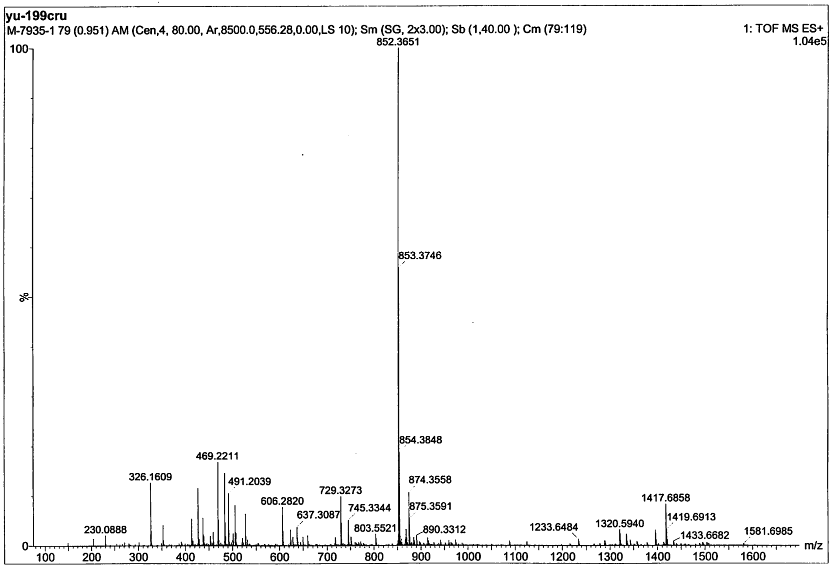

Py$_3$-γ-Im$_3$-OEt (**34**) ^{1}H-NMR ($CDCl_3$)

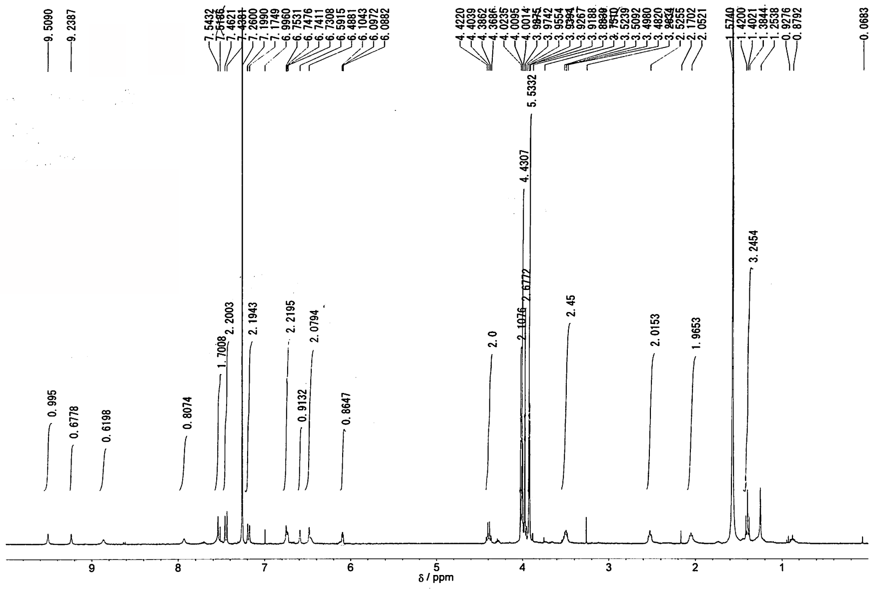

Py$_3$-γ-Im$_3$-OEt (**34**) ^{13}C-NMR ($CDCl_3$)

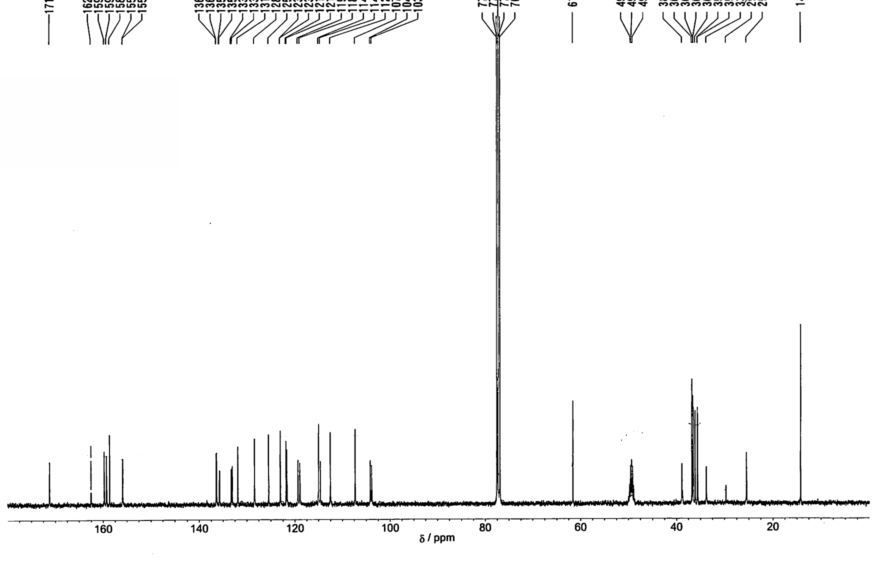

Py_3-γ-Im_3-OH (carboxylic acid)

Single Mass Analysis
Tolerance = 10.0 mDa / DBE: min = -1.5, max = 300.0
Element prediction: Off
Number of isotope peaks used for i-FIT = 3

Monoisotopic Mass, Even Electron Ions
8 formula(e) evaluated with 1 results within limits (up to 50 closest results for each mass)
Elements Used:
C: 0-300 H: 0-3000 N: 15-15 O: 8-8
mt1-p132
M-19806 162 (1.647) AM2 (Ar,22000.0,0.00,0.00); ABS; Cm (153:183) **1: TOF MS ES+ 4.62e+007**

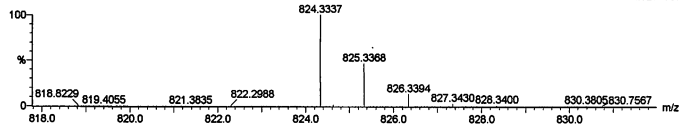

Minimum:				-1.5				
Maximum:		10.0	5.0	300.0				
Mass	Calc. Mass	mDa	PPM	DBE	i-FIT	Norm	Conf(%)	Formula
824.3337	824.3341	-0.4	-0.5	24.5	502.7	n/a	n/a	C37 H42 N15 O8

Py_3-γ-Im_3-OH (carboxylic acid)

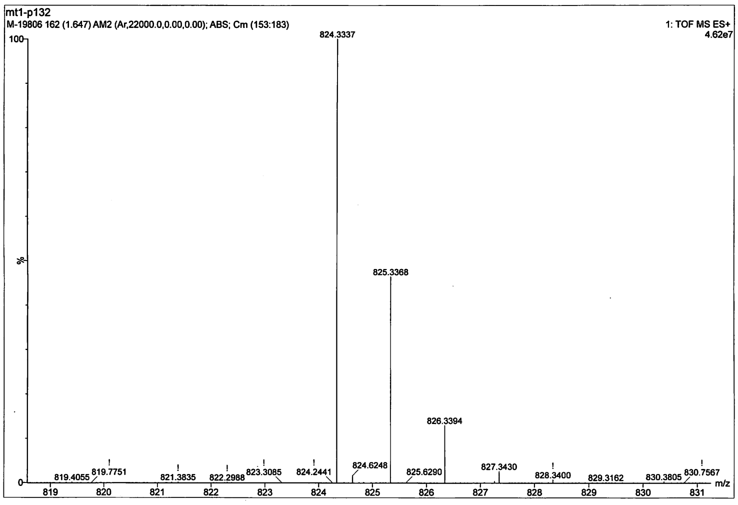

Py$_3$-γ-Im$_3$-OH (carboxylic acid) ^{1}H-NMR (DMSO-d_6)

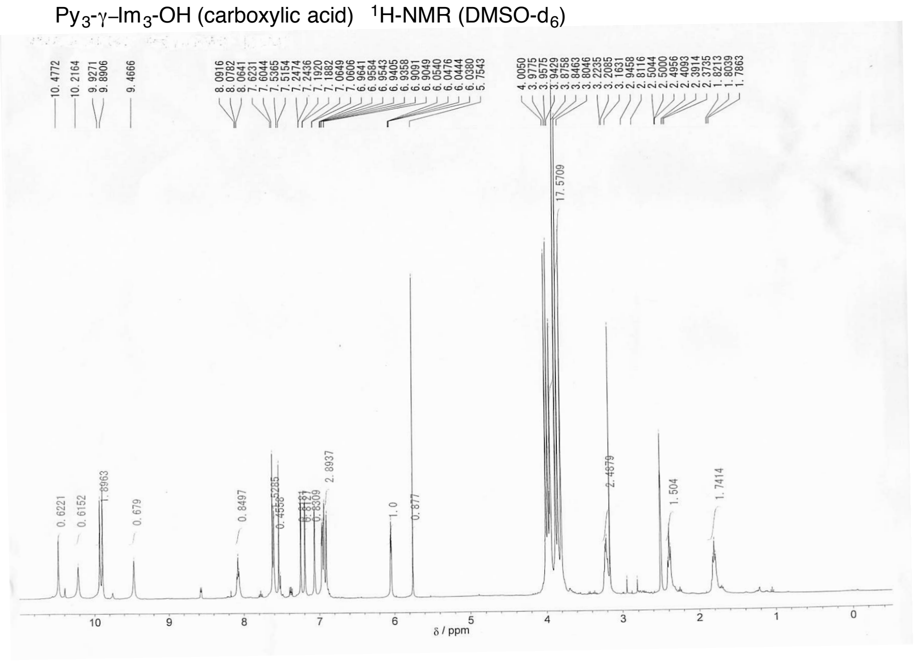

Py$_3$-γ-Im$_3$-OH (carboxylic acid) ^{13}C-NMR (DMSO-d_6)

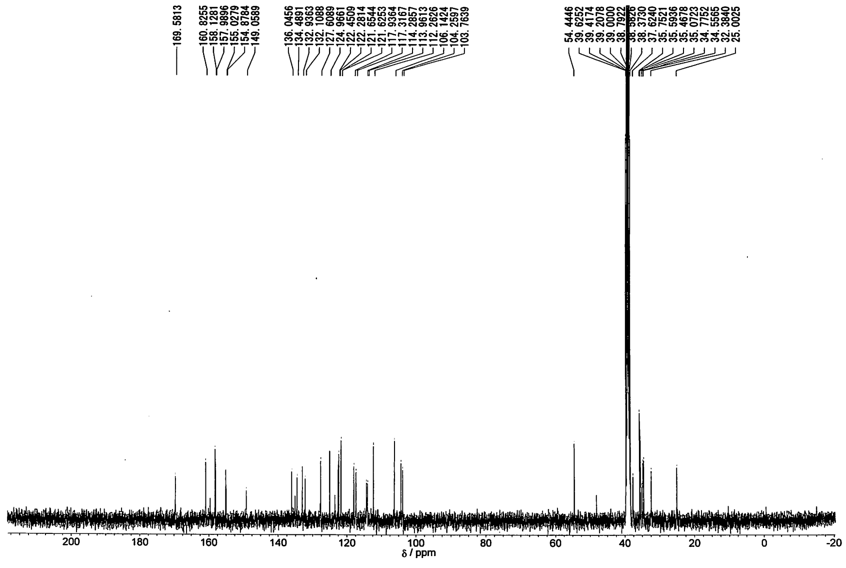

Py$_3$-γ-Im$_3$-NH(CH$_2$)$_2$NHFmoc (**35**)

Elemental Composition Report

Single Mass Analysis
Tolerance = 20.0 PPM / DBE: min = -1.5, max = 300.0
Element prediction: Off
Number of isotope peaks used for i-FIT = 3

Monoisotopic Mass, Even Electron Ions
10 formula(e) evaluated with 1 results within limits (up to 20 closest results for each mass)
Elements Used:
C: 1-300 H: 1-1000 N: 17-17 O: 9-9
P160C2
M-9249 187 (2.223) AM (Cen,4, 80.00, Ar,8500.0,556.28,0.00,LS 10); Sm (SG, 2x3.00); Sb (1,40.00); Cm (183:216) 1: TOF MS ES+ 1.20e+004

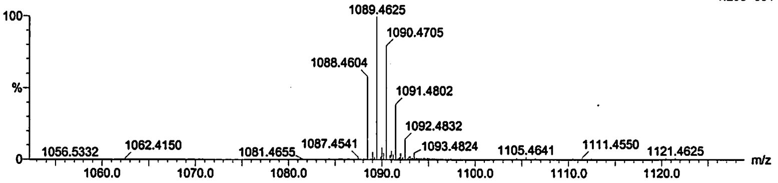

Minimum:				-1.5		
Maximum:		100.0	20.0	300.0		
Mass	Calc. Mass	mDa	PPM	DBE	i-FIT	Formula
1088.4604	1088.4603	0.1	0.1	34.5	4443.2	C54 H58 N17 O9

Py$_3$-γ-Im$_3$-NH(CH$_2$)$_2$NHFmoc (**35**)

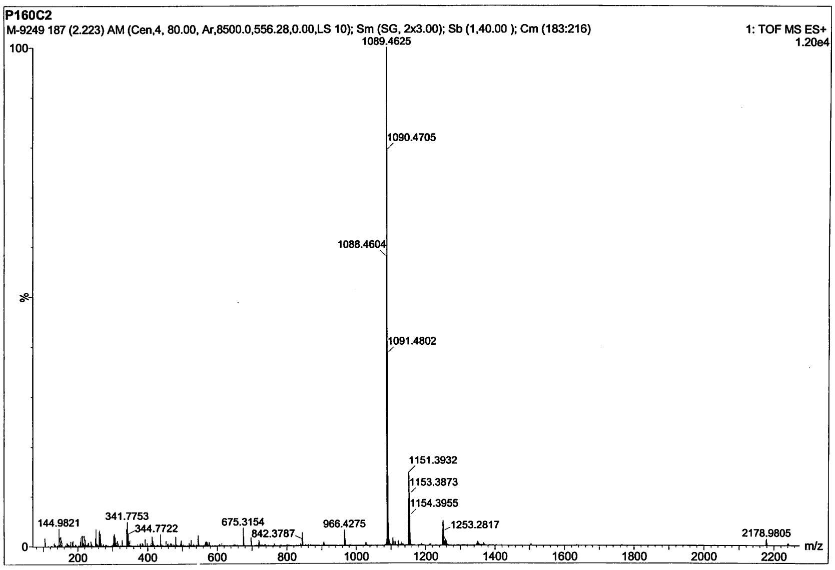

Py$_3$-γ–Im$_3$-NH(CH$_2$)$_2$NHFmoc (**35**) ^{1}H-NMR (DMSO-d$_6$)

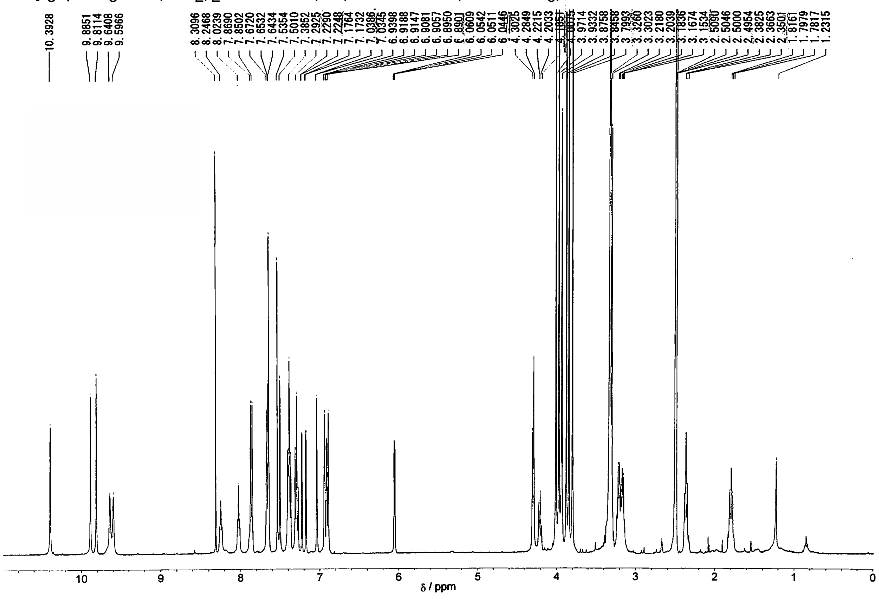

Py$_3$-γ–Im$_3$-NH(CH$_2$)$_2$NHFmoc (**35**) ^{13}C-NMR (CDCl$_3$)

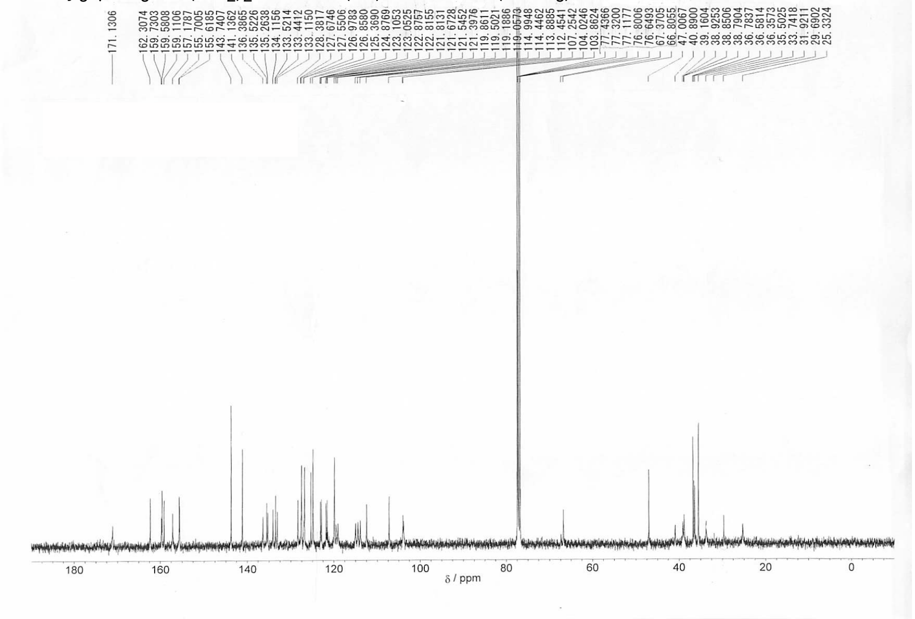

Py_3-γ-Im_3-NH$(CH_2)_3$NHFmoc (**36**)

Elemental Composition Report

Single Mass Analysis
Tolerance = 20.0 PPM / DBE: min = -1.5, max = 300.0
Element prediction: Off
Number of isotope peaks used for i-FIT = 3

Monoisotopic Mass, Even Electron Ions
10 formula(e) evaluated with 1 results within limits (up to 20 closest results for each mass)
Elements Used:
C: 1-300 H: 1-1000 N: 17-17 O: 9-9
yu2-11-3
M-8017 616 (7.298) AM (Cen,4, 80.00, Ar,8500.0,556.28,0.00,LS 10); Sm (SG, 2x3.00); Sb (1,40.00); Cm (596:630) 1: TOF MS ES+ 3.22e+004

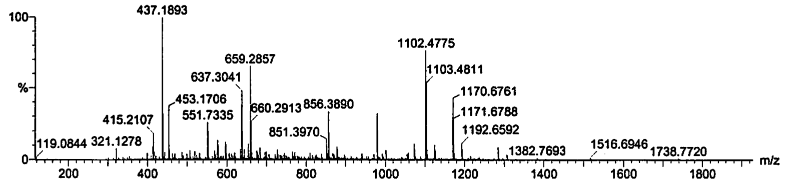

	Calc. Mass	mDa	PPM	DBE	i-FIT	Formula
Minimum:				-1.5		
Maximum:		100.0	20.0	300.0		
Mass	Calc. Mass	mDa	PPM	DBE	i-FIT	Formula
1102.4775	1102.4760	1.5	1.4	34.5	31.9	C55 H60 N17 O9

Py_3-γ-Im_3-NH$(CH_2)_3$NHFmoc (**36**)

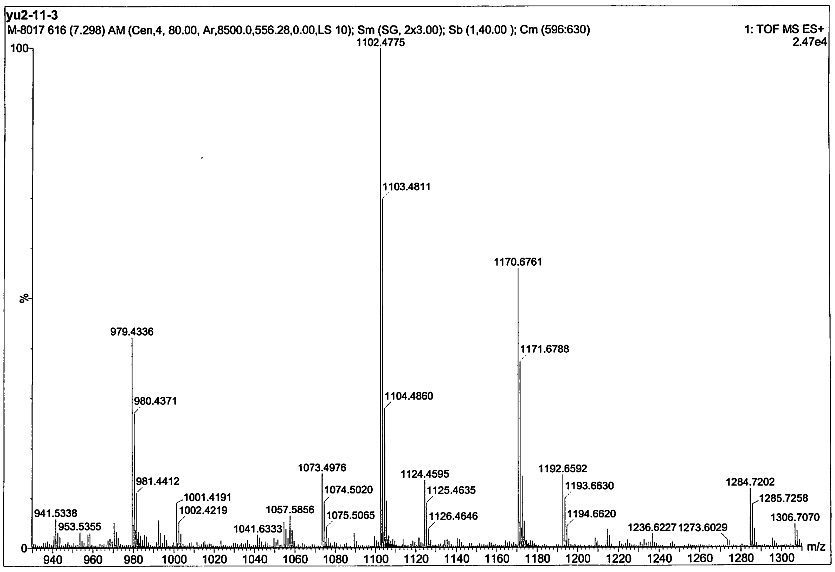

Py$_3$-γ–Im$_3$-NH(CH$_2$)$_3$NHFmoc (**36**) ^{1}H-NMR (DMSO-d$_6$)

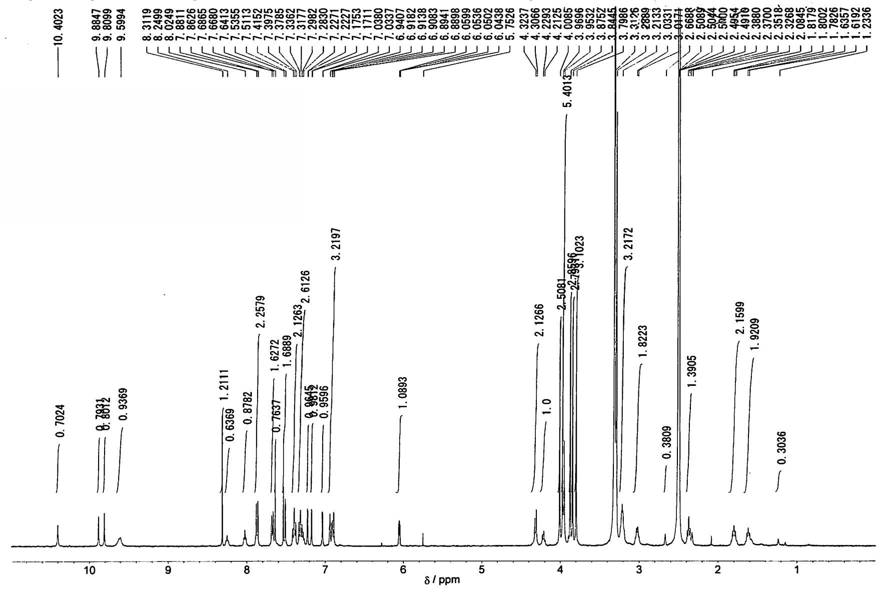

Py$_3$-γ–Im$_3$-NH(CH$_2$)$_3$NHFmoc (**36**) ^{13}C-NMR (DMSO-d$_6$)

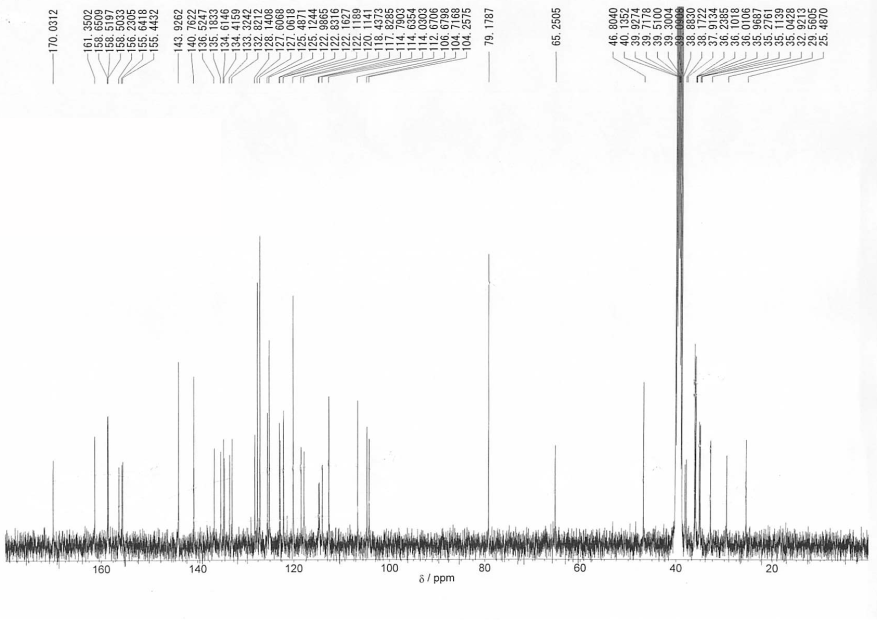

Py$_3$-γ-Im$_3$-NH(CH$_2$)$_4$NHFmoc (**37**)

Elemental Composition Report

Single Mass Analysis
Tolerance = 20.0 PPM / DBE: min = -1.5, max = 300.0
Element prediction: Off
Number of isotope peaks used for i-FIT = 3

Monoisotopic Mass, Even Electron Ions
11 formula(e) evaluated with 1 results within limits (up to 20 closest results for each mass)
Elements Used:
C: 1-300 H: 1-1000 N: 17-17 O: 9-9

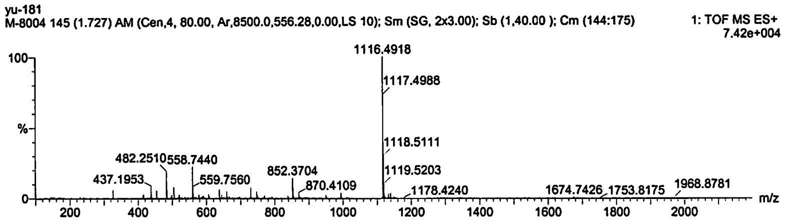

Minimum:				-1.5		
Maximum:		100.0	20.0	300.0		
Mass	Calc. Mass	mDa	PPM	DBE	i-FIT	Formula
1116.4918	1116.4916	0.2	0.2	34.5	207.8	C56 H62 N17 O9

Py$_3$-γ-Im$_3$-NH(CH$_2$)$_4$NHFmoc (**37**)

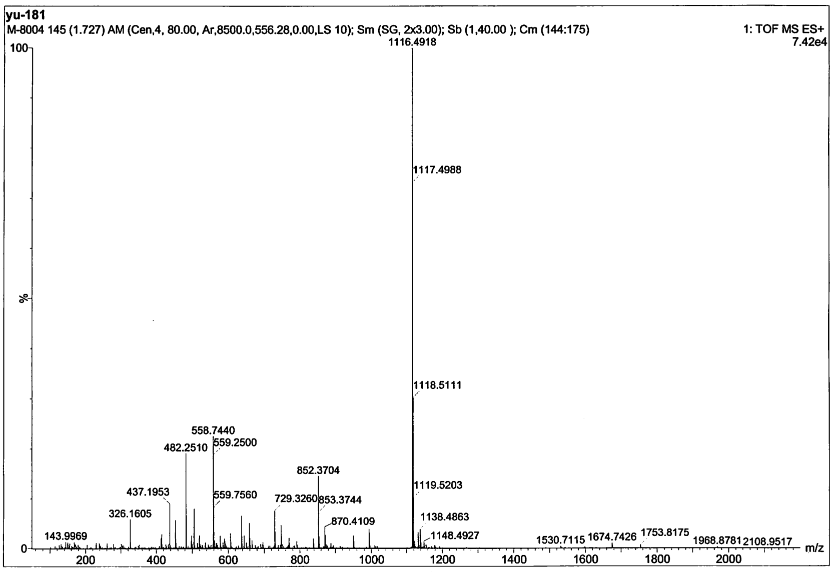

Py$_3$-γ-Im$_3$-NH(CH$_2$)$_4$NHFmoc (**37**) ^{1}H-NMR (DMSO-d$_6$)

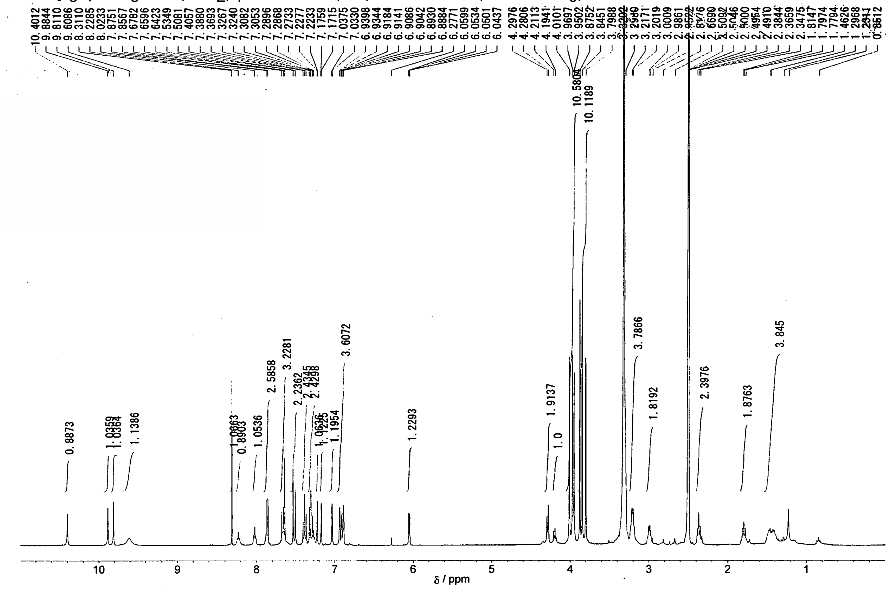

Py$_3$-γ-Im$_3$-NH(CH$_2$)$_4$NHFmoc (**37**) ^{13}C-NMR (DMSO-d$_6$)

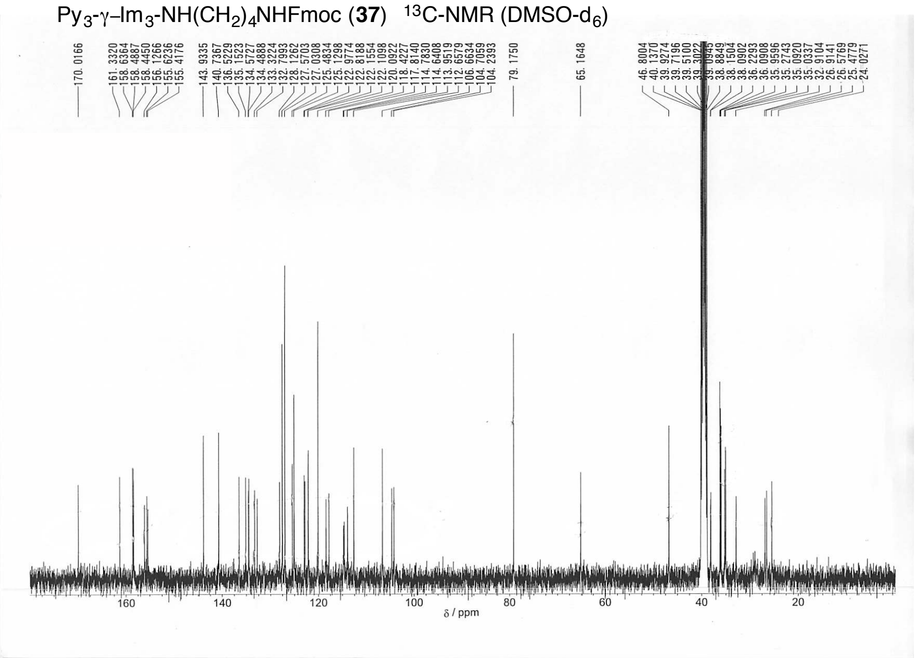

Figure S2: UV spectra of MGB amide compounds **4**, **6**, **28** and **34**.

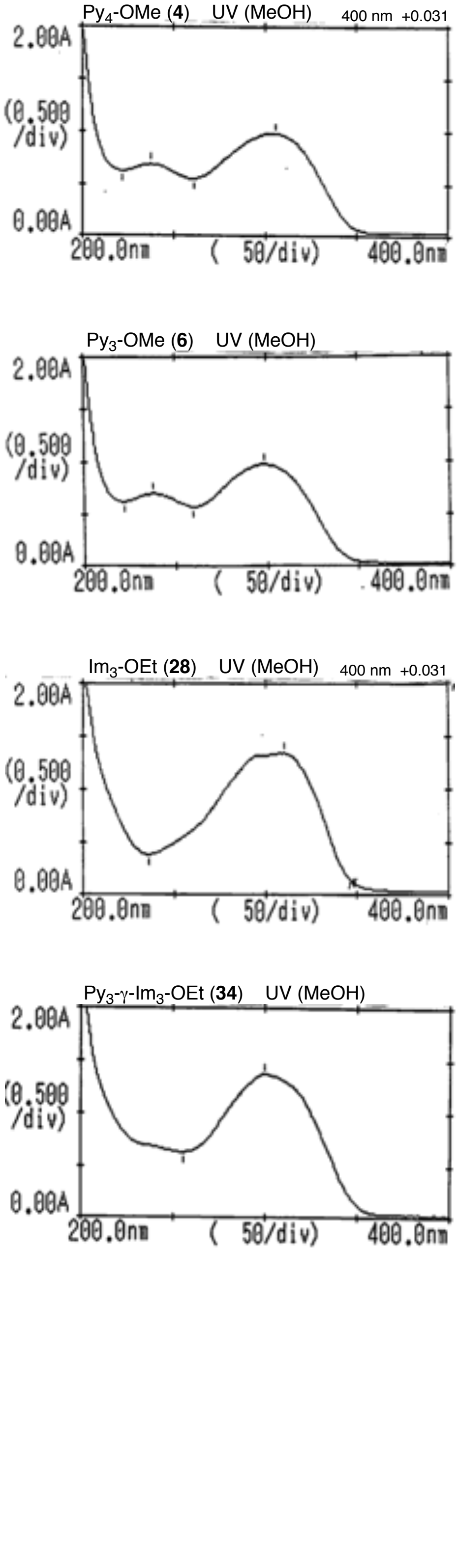

Figure S3: HPLC charts of MGB polyamide-oligonucleotide conjugates.

(A) HPLC chart of crude products of the preparation of modified DNA.

(B) HPLC chart of the modified DNA isolated from crude products (A).

HPLC conditions: Detection: UV 260 nm, flow rate: 1.0 mL/min, mobile phase: 5-50% CH_3CN in water (0.01 M TEAA, pH 7), column: μBONDASPHARE C18 5 μm 100A (3.9 mm ID x 150 mm L)

5'-d(CGGAATTTGGC)-3'

G = Py_4-NH-(CH_2)n-G, ON 1 (n = 3, 4, 5), G = Py_3-NH-(CH_2)n-G, ON 2 (n = 4)

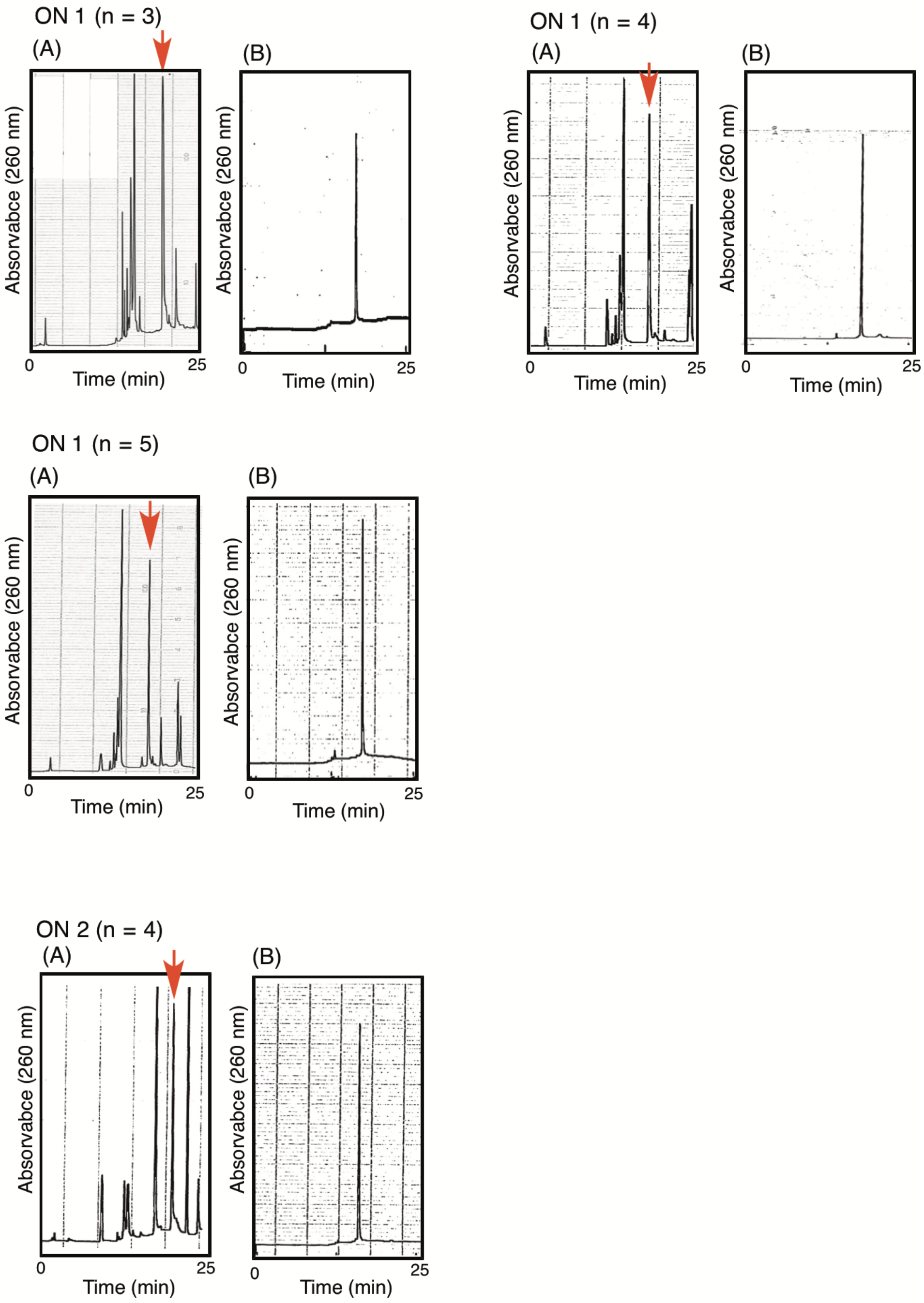

5'-d(CG<u>G</u>ACCCTGGC)-3'

<u>G</u> = Im_{3}-NH-(CH_2)n-G, ON 3 (n = 3, 4, 5), <u>G</u> = Py_{3}-γ-Im_{3}-NH-(CH_2)n-G, ON 4 (n = 2, 3, 4)

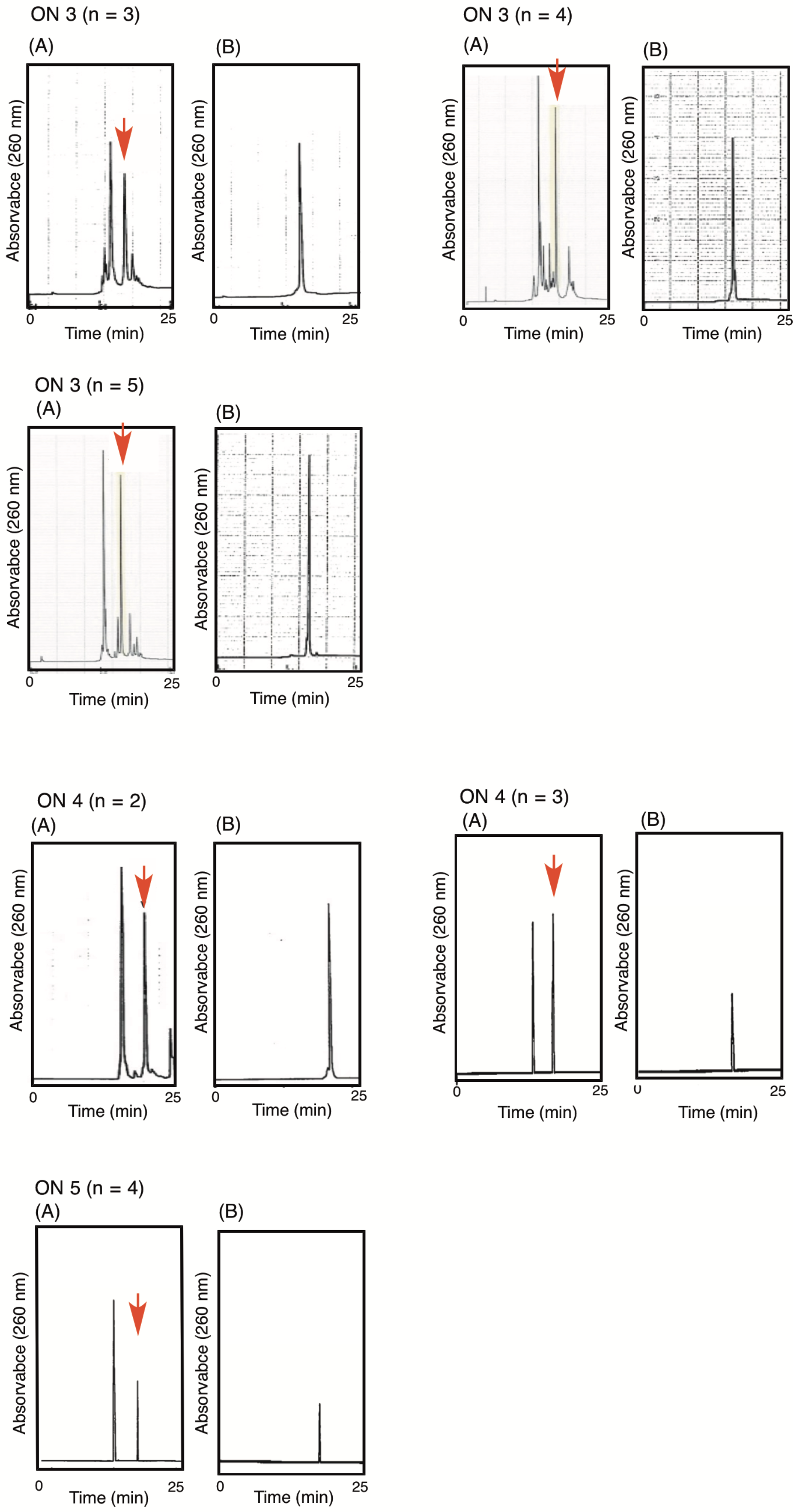

Figure S4: Mass spectra of MGB polyamide-oligonucleotide conjugates.

Py_4-$NH(CH_2)_3$-ON [**ON 1** (n = 3)]

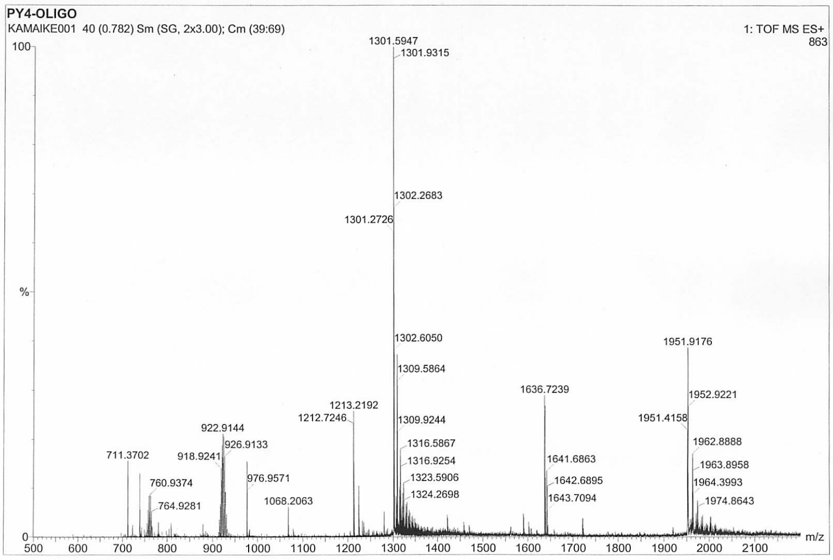

Py_4-$NH(CH_2)_3$-ON [**ON 1** (n = 3)]

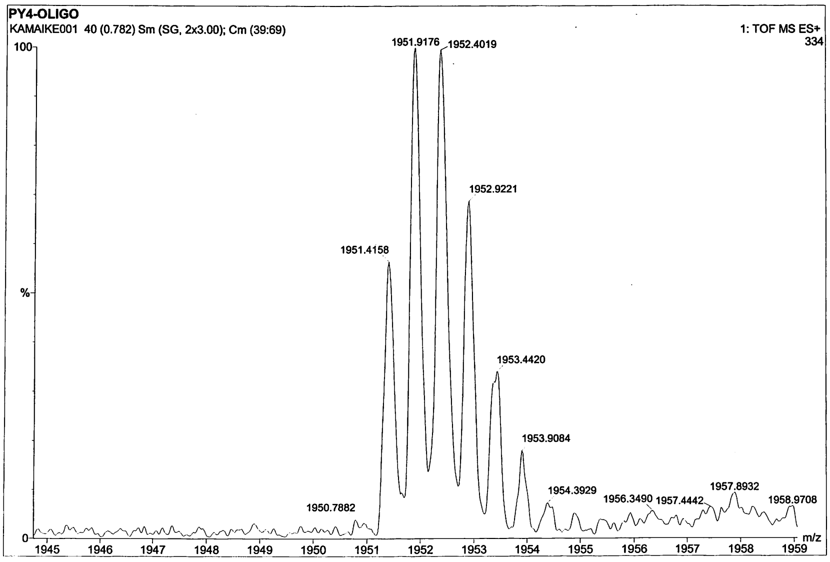

Py$_4$-NH(CH$_2$)$_4$-ON [**ON 1** (n = 4)]

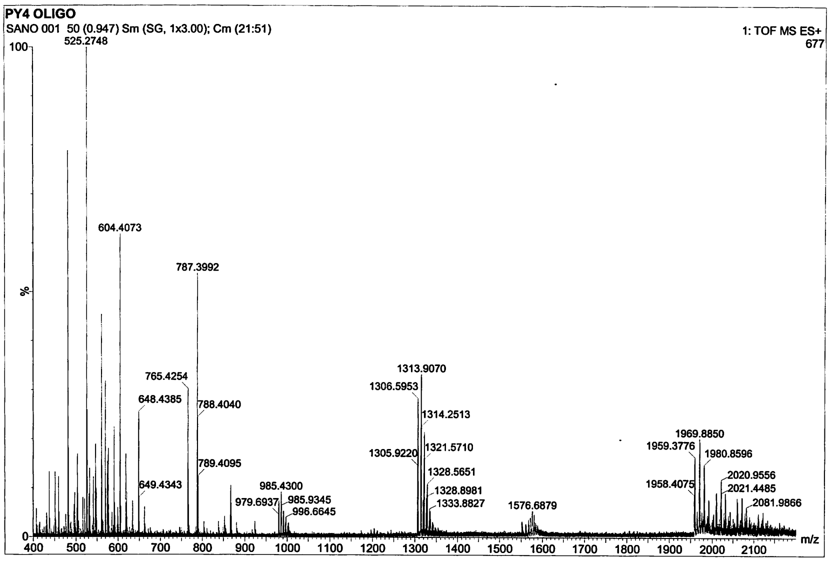

Py$_4$-NH(CH$_2$)$_4$-ON [**ON 1** (n = 4)]

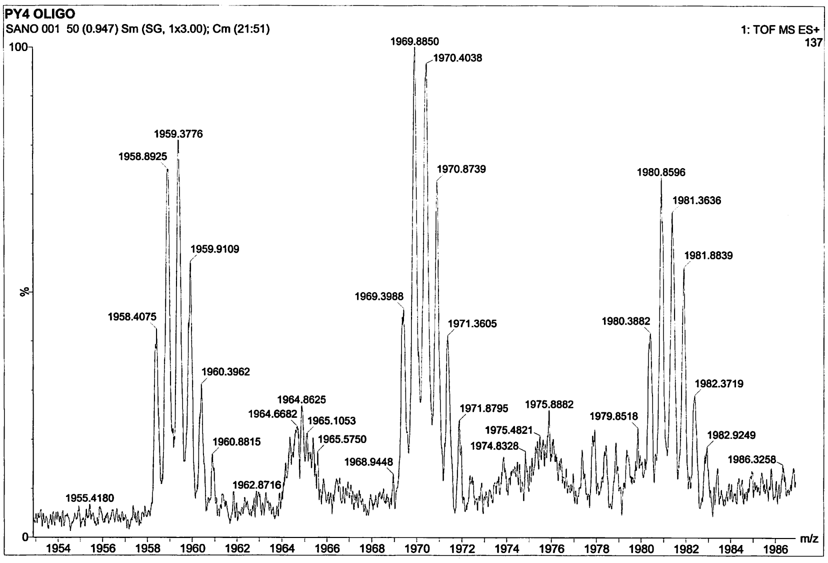

Py$_4$-NH(CH$_2$)$_5$-ON [**ON 1** (n = 5)]

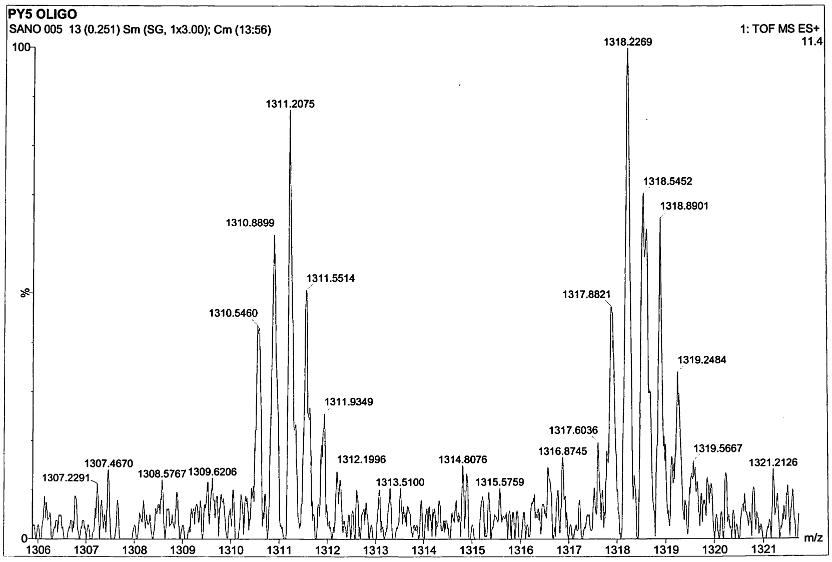

Py_3-$NH(CH_2)_4$-ON [ON 2 (n = 4)]

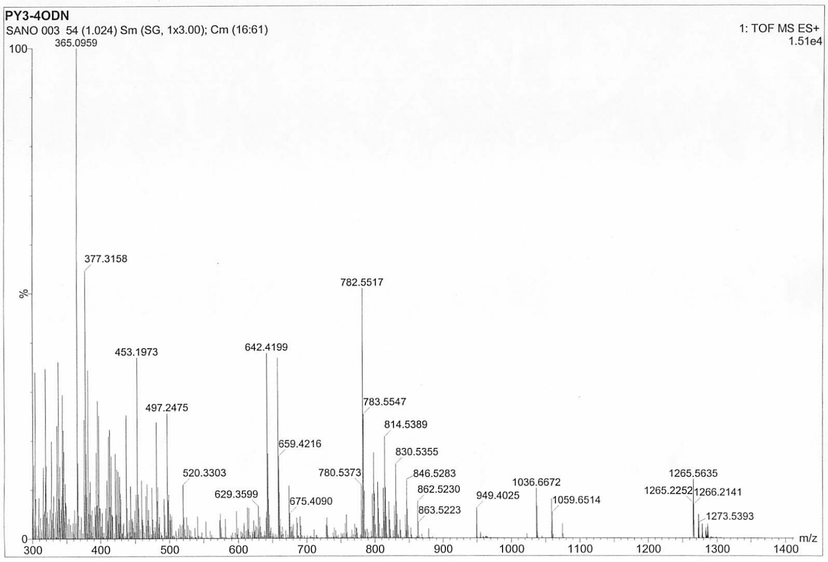

Py_3-$NH(CH_2)_4$-ON [ON 2 (n = 4)]

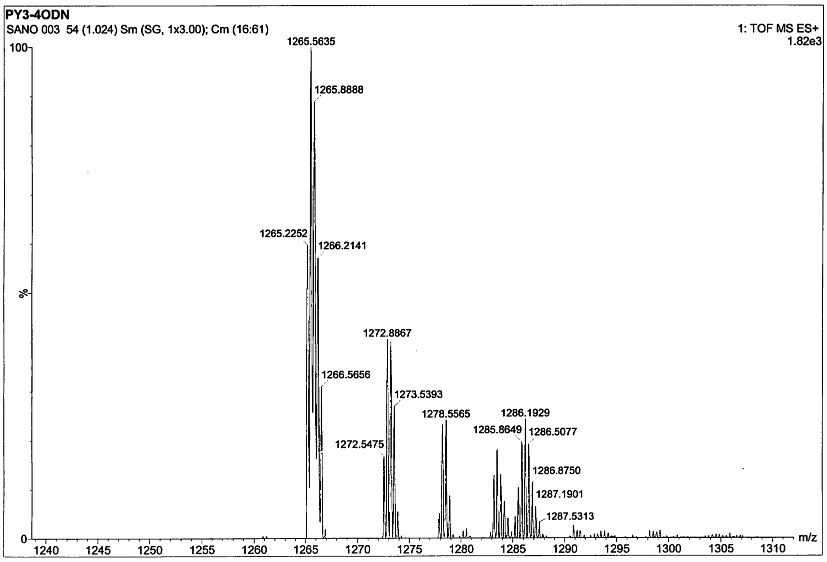

Im_3-NH$(CH_2)_3$-ON [**ON 3** (n = 3)]

Elemental Composition Report

Single Mass Analysis
Tolerance = 100.0 mDa / DBE: min = -1.5, max = 300.0
Element prediction: Off
Number of isotope peaks used for i-FIT = 3

Monoisotopic Mass, Even Electron Ions
1 formula(e) evaluated with 1 results within limits (up to 50 closest results for each mass)
Elements Used:
C: 123-123 H: 1-3000 N: 51-51 O: 67-67 P: 10-10
saka-146A 1.56e+003
SAKATA 001 807 (9.566) M3 [Ev-75272,It50,En1] (25000.0,4,Pep,Cmp); Mk [Ev-2296,It50,En1] (25000.0,4,Pep,Cmp); Sm (SG, 3x3.00); Sb (10,10.00

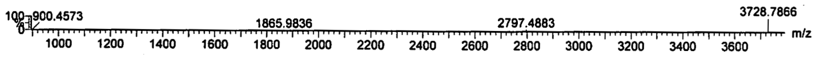

Mass	Calc. Mass	mDa	PPM	DBE	i-FIT	Norm	Conf(%)	Formula
Minimum:				-1.5				
Maximum:		100.0	10.0	300.0				
3728.7866	3728.7744	12.2	3.3	76.5	28.6	n/a	n/a	C123 H156 N51 O67 P10

Im_3-NH$(CH_2)_3$-ON [**ON 3** (n = 3)]

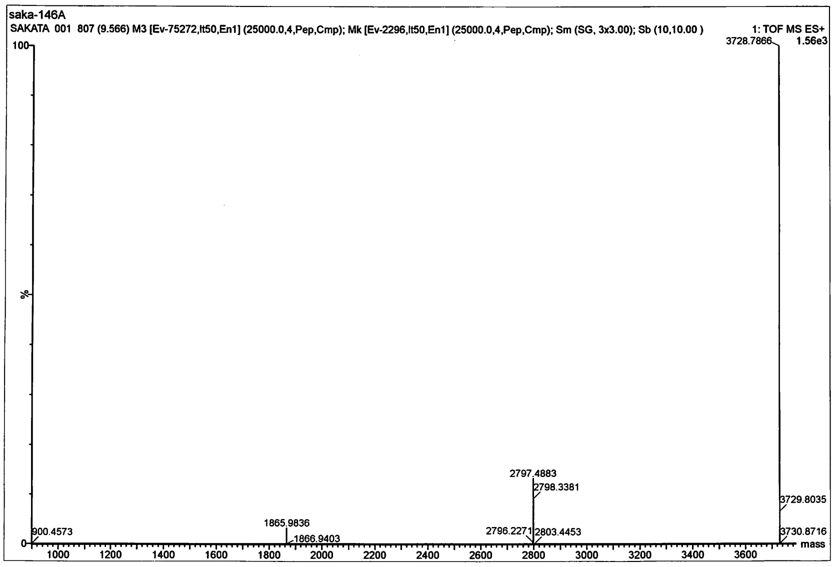

Im_3-NH(CH_2)$_4$-ON [**ON 3** (n = 4)]

Elemental Composition Report

Single Mass Analysis
Tolerance = 100.0 mDa / DBE: min = -1.5, max = 300.0
Element prediction: Off
Number of isotope peaks used for i-FIT = 3

Monoisotopic Mass, Even Electron Ions
1 formula(e) evaluated with 1 results within limits (up to 50 closest results for each mass)
Elements Used:
C: 124-124 H: 1-3000 N: 51-51 O: 67-67 P: 10-10

saka-167
SAKATA 003 403 (4.782) M3 [Ev-246599,It50,En1] (25000.0,4,Pep,Cmp); Sm (SG, 3x3.00); Sb (10,10.00); Cm (365:412) 1: TOF MS ES+ 6.99e+002

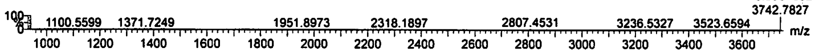

Mass	Calc. Mass	mDa	PPM	DBE	i-FIT	Norm	Conf(%)	Formula
Minimum:				-1.5				
Maximum:		100.0	10.0	300.0				
3742.7827	3742.7900	-7.3	-2.0	76.5	26.4	n/a	n/a	C124 H158 N51 O67 P10

Im_3-NH(CH_2)$_4$-ON [**ON 3** (n = 4)]

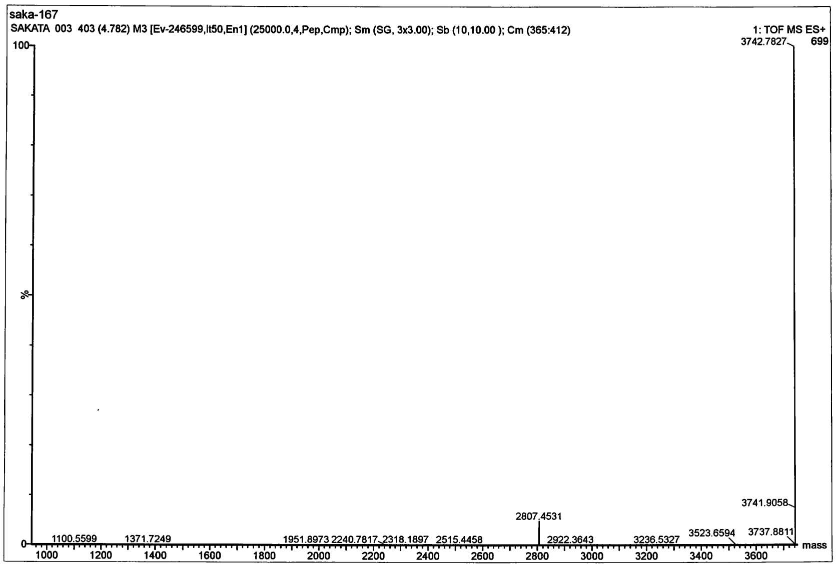

Im$_3$-NH(CH$_2$)$_5$-ON [**ON 3** (n = 5)]

Elemental Composition Report

Single Mass Analysis
Tolerance = 100.0 mDa / DBE: min = -1.5, max = 300.0
Element prediction: Off
Number of isotope peaks used for i-FIT = 3

Monoisotopic Mass, Even Electron Ions
1 formula(e) evaluated with 1 results within limits (up to 50 closest results for each mass)
Elements Used:
C: 125-125 H: 1-3000 N: 51-51 O: 67-67 P: 10-10

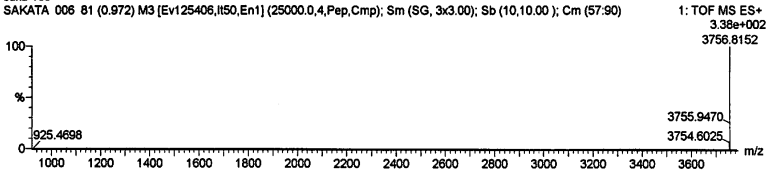

Mass	Calc. Mass	mDa	PPM	DBE	i-FIT	Norm	Conf(%)	Formula
Minimum:				-1.5				
Maximum:		100.0	10.0	300.0				
3756.8152	3756.8057	9.5	2.5	76.5	29.1	n/a	n/a	C125 H160 N51 O67 P10

Im$_3$-NH(CH$_2$)$_5$-ON [**ON 3** (n = 5)]

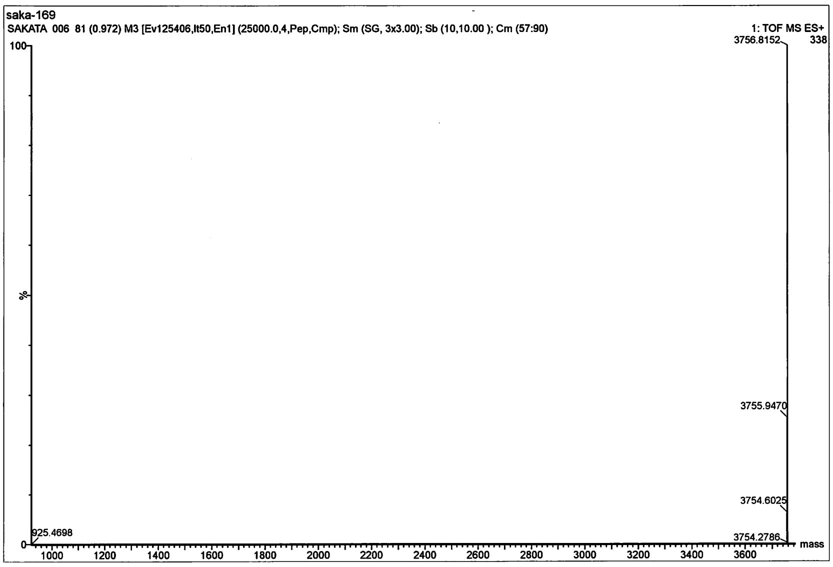

Py_3-γ-Im_3-NH$(CH_2)_2$-ON [**ON 4** (n = 2)]

Elemental Composition Report

Single Mass Analysis
Tolerance = 20.0 PPM / DBE: min = -1.5, max = 300.0
Element prediction: Off
Number of isotope peaks used for i-FIT = 3

Monoisotopic Mass, Even Electron Ions
28 formula(e) evaluated with 1 results within limits (up to 50 closest results for each mass)
Elements Used:
C: 1-300 H: 1-1000 N: 58-58 O: 71-71 P: 10-10
2P13-11C2
M-10469 175 (3.652) M3 [Ev-1727827,It50,En1] (25000.0,2,Pep,Cmp); Cm (174:214)

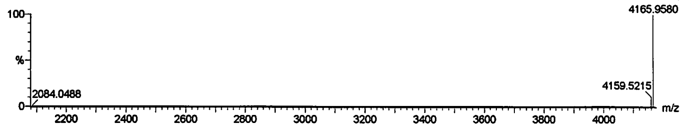

Minimum:				-1.5				
Maximum:		20.0	20.0	300.0				
Mass	Calc. Mass	mDa	PPM	DBE	i-FIT	Norm	Conf(%)	Formula
4165.9580	4165.9555	2.5	0.6	89.5	45.5	n/a	n/a	C144 H179 N58 O71 P10

Py_3-γ-Im_3-NH$(CH_2)_2$-ON [**ON 4** (n = 2)]

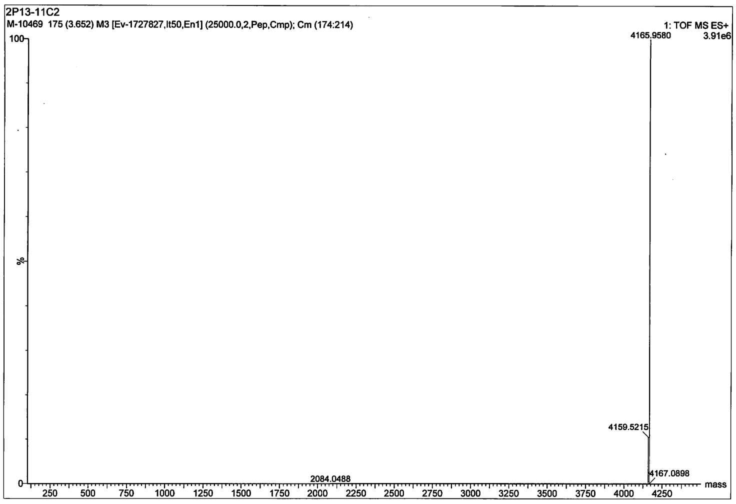

Py_3-γ-Im_3-NH$(CH_2)_3$-ON [**ON 4** (n = 3)]

Elemental Composition Report

Single Mass Analysis
Tolerance = 5.0 PPM / DBE: min = -1.5, max = 300.0
Element prediction: Off
Number of isotope peaks used for i-FIT = 3

Monoisotopic Mass, Even Electron Ions
29 formula(e) evaluated with 1 results within limits (up to 20 closest results for each mass)
Elements Used:
C: 1-300 H: 1-1000 N: 58-58 O: 71-71 P: 10-10
yu2-16 1b 3.22e+004
M-8218 154 (1.832) M3 [Ev-804306,It50,En1] (0.030,200.00,0.100,1000.00,2,Cmp); Sm (SG, 9x3.00); Sb (1,40.00); Cm (148:278)

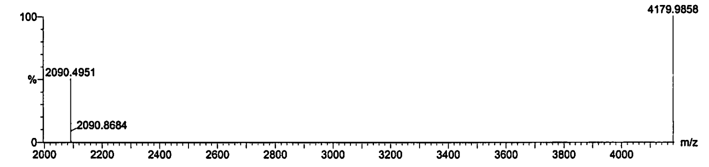

	Mass	Calc. Mass	mDa	PPM	DBE	i-FIT	Formula
Minimum:					-1.5		
Maximum:			100.0	5.0	300.0		
	4179.9858	4179.9712	14.6	3.5	89.5	n/a	C145 H181 N58 O71 P10

Py_3-γ-Im_3-NH$(CH_2)_3$-ON [**ON 4** (n = 3)]

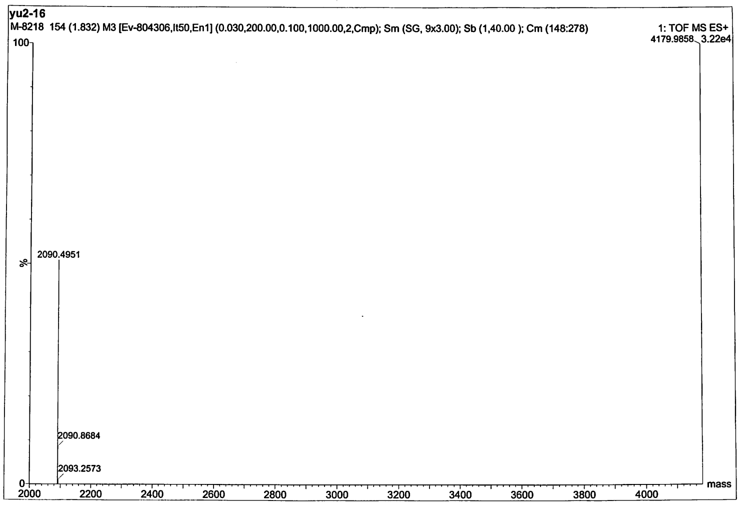

Py$_3$-γ-Im$_3$-NH(CH$_2$)$_4$-ON [**ON 4** (n = 4)]

Elemental Composition Report

Single Mass Analysis
Tolerance = 5.0 PPM / DBE: min = 0.5, max = 500.0
Element prediction: Off
Number of isotope peaks used for i-FIT = 4

Monoisotopic Mass, Even Electron Ions
11 formula(e) evaluated with 1 results within limits (up to 20 closest results for each mass)
Elements Used:
C: 146-146 H: 1-1000 N: 58-58 O: 71-71 P: 0-10

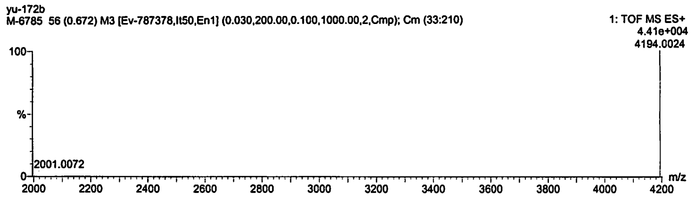

Minimum:				0.5			
Maximum:		100.0	5.0	500.0			
Mass	Calc. Mass	mDa	PPM	DBE	i-FIT	Formula	
4194.0024	4193.9868	15.6	3.7	89.5	n/a	C146 H183 N58 O71 P10	

Py$_3$-γ-Im$_3$-NH(CH$_2$)$_4$-ON [**ON 4** (n = 4)]

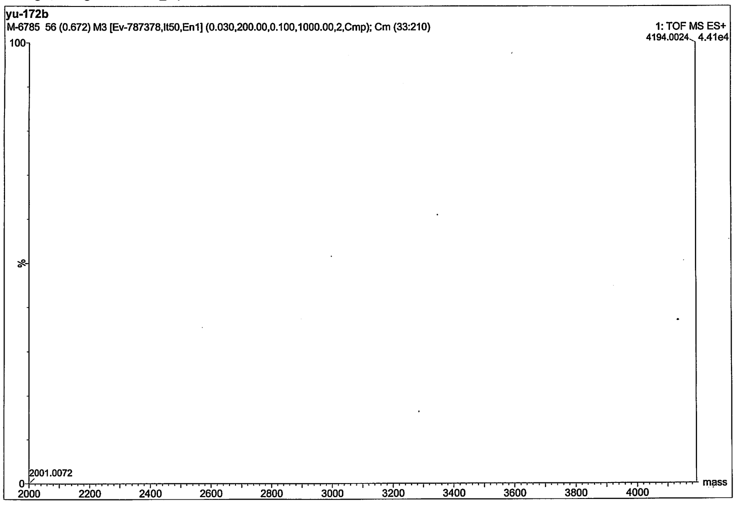

Figure S5: UV melting curves of modified dsDNA (4.3 μM) in 10 mM sodium phosphate buffer (pH 7.0) containing 10 mM NaCl and 0.1 mM Na_2EDTA.

The melting temperature (T_m) was obtained using a TMSPC-8 with T_m analysis software.

Modified dsDNA: 5'-d(CGGAATTTGGC)-3'/complementary DNA

ON 1 [G = Py_4-$NH(CH_2)_4$-G], **ON 2** [G = Py_3-$NH(CH_2)_4$-G]

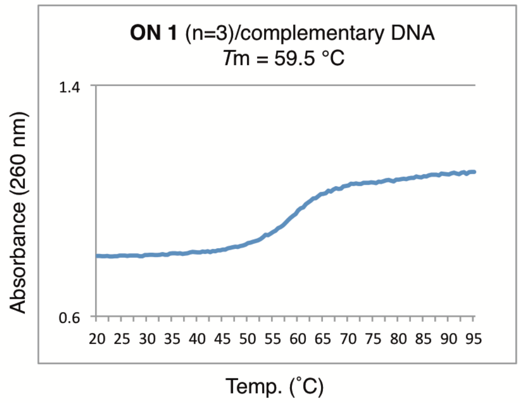

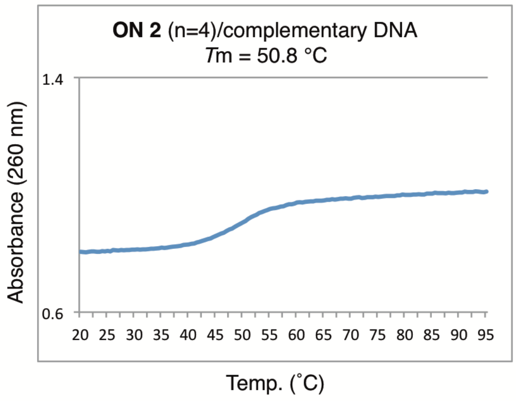

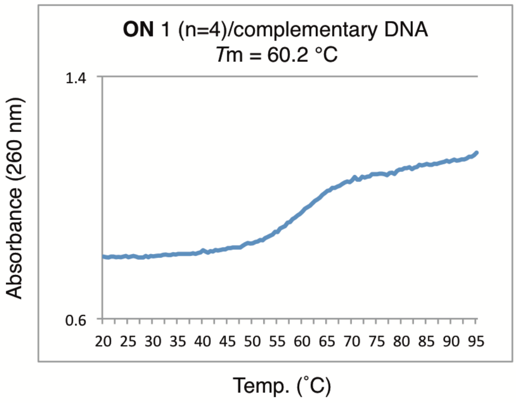

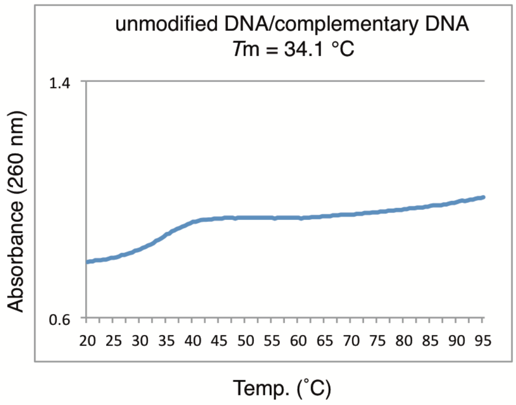

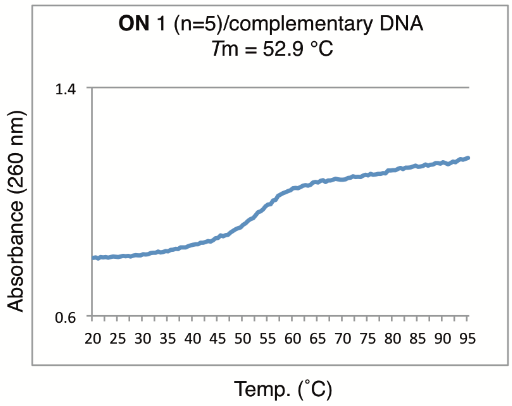

5’-d(CGGAATTTGGC)-3’/mismatch DNA

5’−d(CGGAATTTGGC)−3’
3’−d(GCCTTcAACCG)−5’

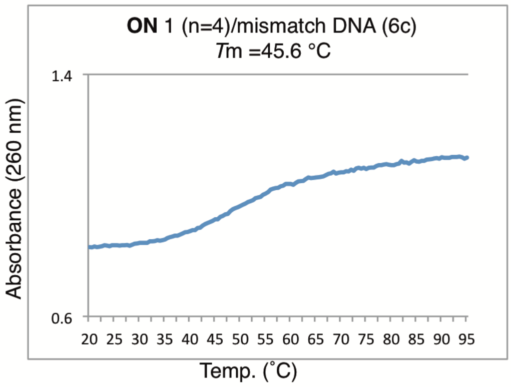

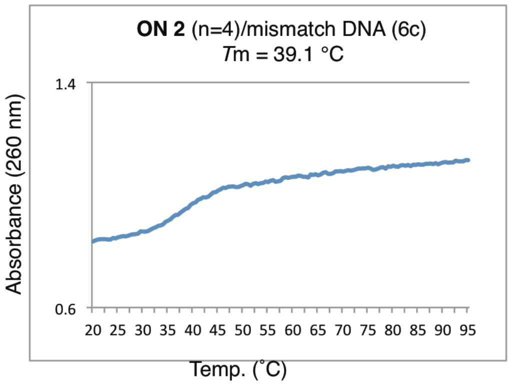

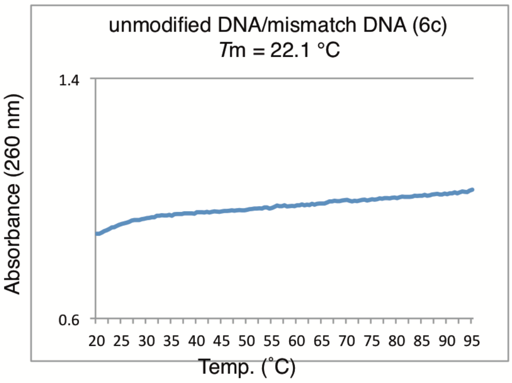

5’−d(CGGAATTTGGC)−3’
3’−d(GCaTTcAACCG)−5’

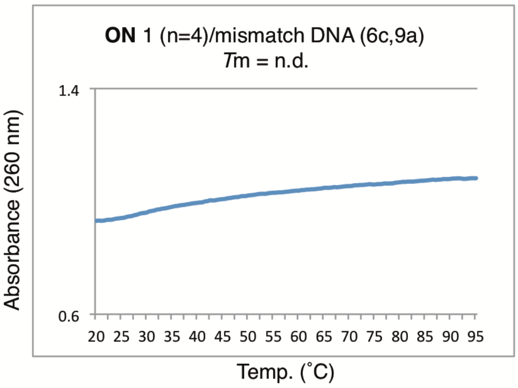

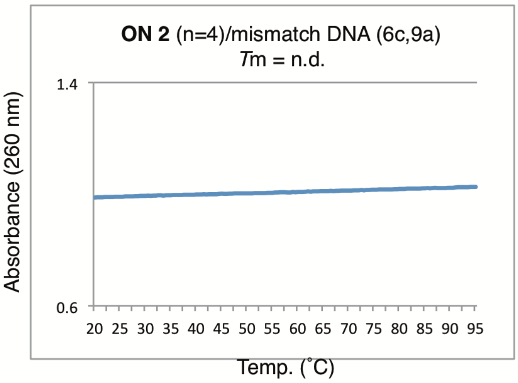

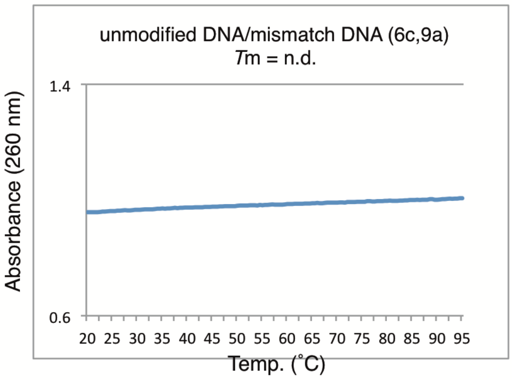

Modified dsDNA: 5’-d(CGGACCCTGGC)-3’/complementary DNA

ON 3 [G = Im_3-$NH(CH_2)_4$-G], **ON 4** [G = Py_3-γ-Im_3-$NH(C_2)_2$-G]

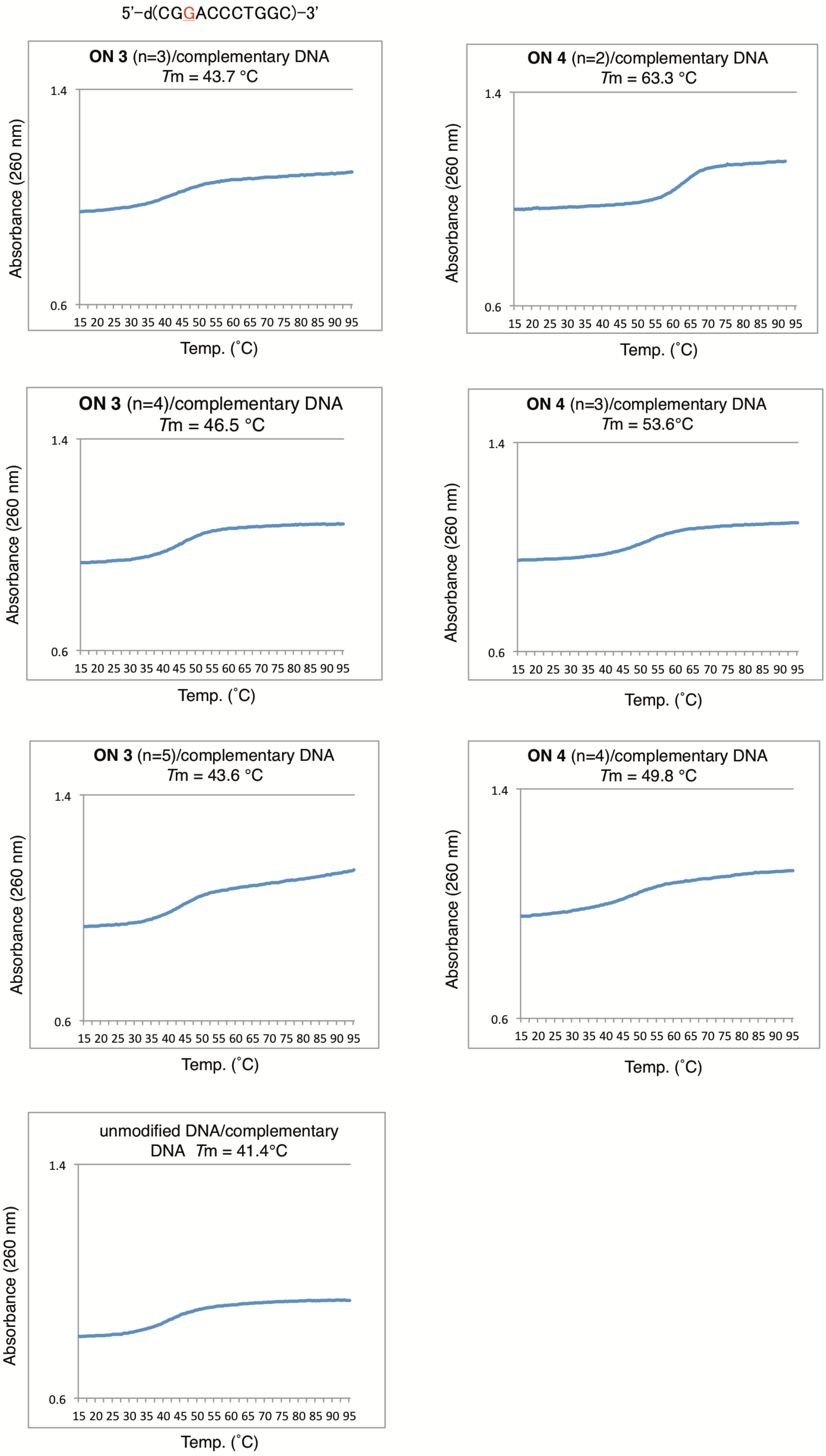

5’-d(CGGACCCTGGC)-3’/mismatch DNA

5’-d(CGGACCCTGGC)-3’
3’-d(GCCTGaGACCG)-5’

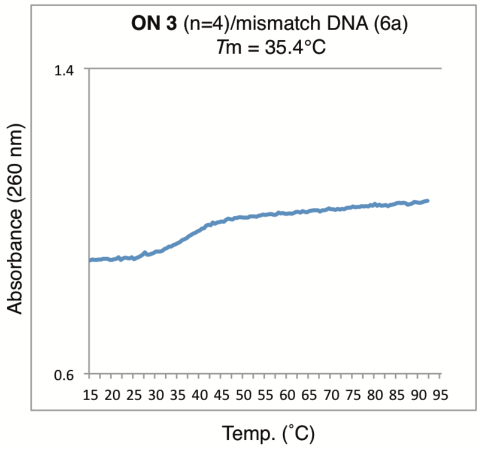

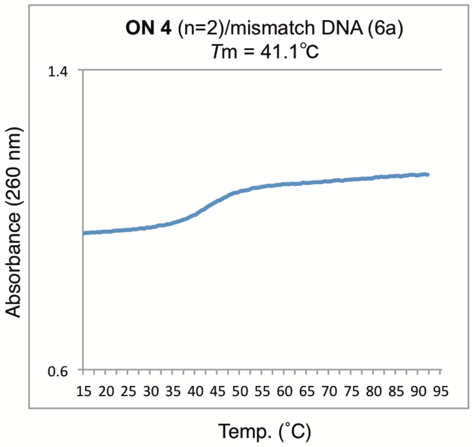

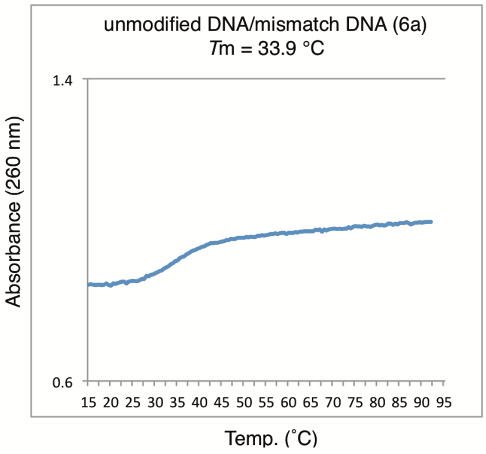

5’-d(CGGACCCTGGC)-3’
3’-d(GCCTGGGACtG)-5’

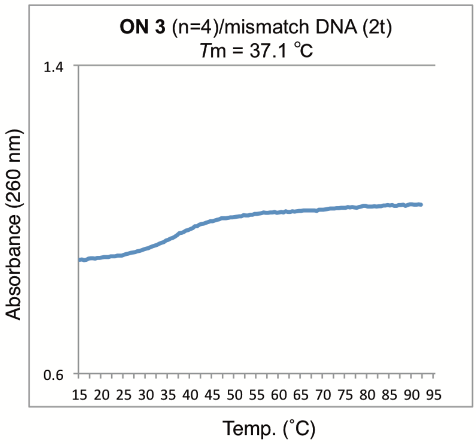

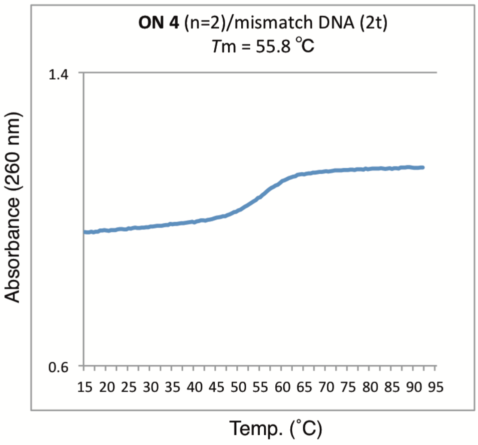

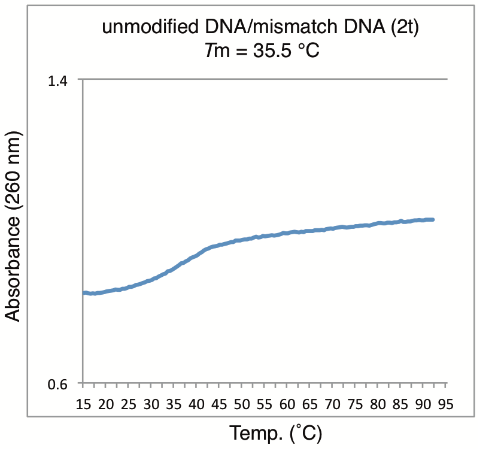

Figure S6: CD spectra of modified dsDNAs (5.8 μM) in 10 mM sodium phosphate buffer (pH 7.0) containing 10 mM NaCl and 0.1 mM Na_2EDTA.

(A) unmodified dsDNA
(B) modified dsDNA [**ON 1** (n = 4)/complementary DNA]

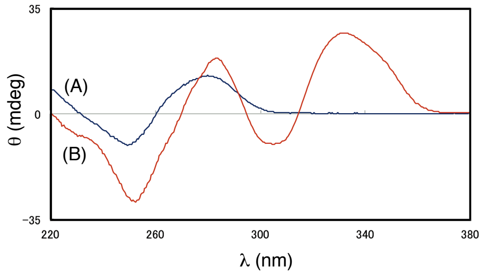

(A) unmodified dsDNA
(C) modified dsDNA [**ON 2** (n = 4)/complementary DNA]

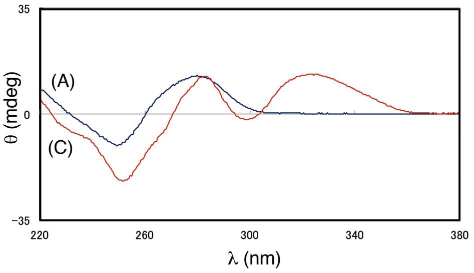

CD spectra of unmodified and modified dsDNAs.

modified DNA : 5'-d(CG**G**AATTTGGC)-3'
ON 1 [**G** = Py_4-$NH(CH_2)_n$-G], **ON 2** [**G** = Py_3-$NH(CH_2)_n$-G]

(D) unmodified dsDNA
(E) modified dsDNA [**ON 3** (n = 4)/complementary DNA]

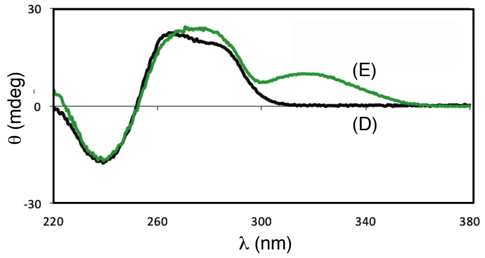

(F) modified dsDNA [**ON 4** (n = 2)/complementary DNA]

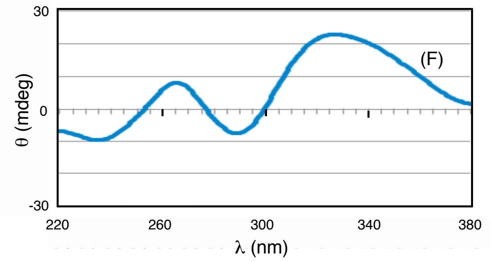

CD spectra of unmodified and modified dsDNAs.

modified DNA : 5'-d(CG**G**ACCCTGGC)-3'
ON 3 [**G** = Im_3-$NH(CH_2)_n$-G], **ON 4** [**G** = Py_3-γ-Im_3-$NH(CH_2)_n$-G]
